# Identification of Novel Associations and Localization of Signals in Idiopathic Inflammatory Myopathies Using Genome‐Wide Imputation

**DOI:** 10.1002/art.42434

**Published:** 2023-03-20

**Authors:** Simon Rothwell, Christopher I. Amos, Frederick W. Miller, Lisa G. Rider, Ingrid E. Lundberg, Peter K. Gregersen, Jiri Vencovsky, Neil McHugh, Vidya Limaye, Albert Selva‐O'Callaghan, Michael G. Hanna, Pedro M. Machado, Lauren M. Pachman, Ann M. Reed, Øyvind Molberg, Olivier Benveniste, Pernille Mathiesen, Timothy Radstake, Andrea Doria, Jan L. De Bleecker, Boel De Paepe, Britta Maurer, William E. Ollier, Leonid Padyukov, Terrance P. O'Hanlon, Annette Lee, Lucy R. Wedderburn, Hector Chinoy, Janine A. Lamb, the Myositis Genetics Consortium

**Affiliations:** ^1^ Centre for Genetics and Genomics Versus Arthritis, Centre for Musculoskeletal Research, Faculty of Biology, Medicine and Health University of Manchester Manchester UK; ^2^ Baylor College of Medicine Houston Texas; ^3^ Environmental Autoimmunity Group National Institute of Environmental Health Sciences, NIH Bethesda Maryland; ^4^ Division of Rheumatology, Department of Medicine, Solna, Karolinska Institutet Karolinska University Hospital Stockholm Sweden; ^5^ The Robert S. Boas Center for Genomics and Human Genetics The Feinstein Institute Manhasset New York; ^6^ Institute of Rheumatology and Department of Rheumatology, First Medical Faculty Charles University Prague Czech Republic; ^7^ Department of Pharmacy and Pharmacology University of Bath Bath UK; ^8^ Rheumatology Unit, Royal Adelaide Hospital and Discipline of Medicine Adelaide University Adelaide Australia; ^9^ Internal Medicine Department, Vall d'Hebron General Hospital, Universitat Autonoma de Barcelona Barcelona Spain; ^10^ Department of Neuromuscular Diseases, UCL Queen Square Institute of Neurology University College London London UK; ^11^ Department of Neuromuscular Diseases, UCL Queen Square Institute of Neurology, and Centre for Rheumatology, UCL Division of Medicine University College London London UK; ^12^ Ann & Robert H. Lurie Children's Hospital of Chicago Northwestern University Feinberg School of Medicine Chicago Illinois; ^13^ Department of Pediatrics Duke University Durham North Carolina; ^14^ Department of Rheumatology Oslo University Hospital Oslo Norway; ^15^ Department of Internal Medicine and Clinical Immunology, Pitié‐Salpêtrière Hospital Paris France; ^16^ Paediatric Department, Slagelse Hospital and Paediatric Rheumatology Unit, Rigshospitalet Copenhagen Denmark; ^17^ Department of Rheumatology and Clinical Immunology University Medical Center Utrecht the Netherlands; ^18^ Rheumatology Unit, Department of Medicine University of Padova Padova Italy; ^19^ Department of Neurology Ghent University Ghent Belgium; ^20^ Department of Rheumatology and Immunology University Hospital Bern Switzerland; ^21^ Manchester Metropolitan University, School of Healthcare Sciences Manchester UK; ^22^ NIHR Biomedical Research Centre at Great Ormond Street Hospital, and Arthritis Research UK Centre for Adolescent Rheumatology, UCL Great Ormond Street Institute of Child Health University College London London UK; ^23^ National Institute for Health Research Manchester Biomedical Research Centre, Manchester University NHS Foundation Trust, The University of Manchester, Manchester, UK, and Department of Rheumatology, Salford Royal Hospital, Northern Care Alliance NHS Foundation Trust, Manchester Academic Health Science Centre, Salford, UK, and Centre for Musculoskeletal Research, Faculty of Biology, Medicine and Health, The University of Manchester Manchester UK; ^24^ Epidemiology and Public Health Group, Division of Population Health, Health Services Research & Primary Care, Faculty of Biology, Medicine and Health University of Manchester Manchester UK

## Abstract

**Objective:**

The idiopathic inflammatory myopathies (IIMs) are heterogeneous diseases thought to be initiated by immune activation in genetically predisposed individuals. We imputed variants from the ImmunoChip array using a large reference panel to fine‐map associations and identify novel associations in IIM.

**Methods:**

We analyzed 2,565 Caucasian IIM patient samples collected through the Myositis Genetics Consortium (MYOGEN) and 10,260 ethnically matched control samples. We imputed 1,648,116 variants from the ImmunoChip array using the Haplotype Reference Consortium panel and conducted association analysis on IIM and clinical and serologic subgroups.

**Results:**

The HLA locus was consistently the most significantly associated region. Four non‐HLA regions reached genome‐wide significance, *SDK2* and *LINC00924* (both novel) and *STAT4* in the whole IIM cohort, with evidence of independent variants in *STAT4*, and *NAB1* in the polymyositis (PM) subgroup. We also found suggestive evidence of association with loci previously associated with other autoimmune rheumatic diseases (*TEC* and *LTBR*). We identified more significant associations than those previously reported in IIM for *STAT4* and *DGKQ* in the total cohort, for *NAB1* and *FAM167A*‐*BLK* loci in PM, and for *CCR5* in inclusion body myositis. We found enrichment of variants among DNase I hypersensitivity sites and histone marks associated with active transcription within blood cells.

**Conclusion:**

We found novel and strong associations in IIM and PM and localized signals to single genes and immune cell types.

## INTRODUCTION

The idiopathic inflammatory myopathies (IIMs) are a heterogeneous group of rare autoimmune diseases primarily characterized by muscle weakness with extramuscular manifestations. The strongest genetic risk for IIM resides in the HLA region, although non‐HLA associations have also been reported. To date, the largest genetic association studies have been conducted in Caucasian populations through the Myositis Genetics Consortium (MYOGEN) (1) and subsequent meta‐analyses (2). However, a genome‐wide association study (GWAS) in clinically amyopathic dermatomyositis in the Japanese population (3) and candidate gene studies in Japanese and Chinese populations have also identified significant genetic risk factors for IIM (4, 5, 6).

We previously published a genetic association study on 90,536 genetic variants from ImmunoChip, a targeted array containing coverage of 186 established autoimmune susceptibility loci (1). In this follow‐up study we re‐analyzed the IIM ImmunoChip data set after imputation of 1,648,116 variants to identify novel associations and to facilitate fine‐mapping of risk regions reported in IIM and clinical and serologic subgroups.

## PATIENTS AND METHODS

### Samples

Caucasian IIM patient samples were collected through MYOGEN (1). IIM samples were included if patients fulfilled probable or definite Bohan and Peter classification criteria (7) for polymyositis (PM), juvenile PM, dermatomyositis (DM), or juvenile DM, and Griggs, European Neuromuscular Centre, or Medical Research Council criteria for inclusion body myositis (IBM) (8). ImmunoChip control data from 12 countries were provided by 4 disease consortia. Anti–Jo‐1 (anti–histidyl–transfer RNA) autoantibodies were detected using either immunoprecipitation or line blot using methods described previously (9).

### Genotyping and imputation

Analysis was conducted on the existing Illumina ImmunoChip array data consisting of 2,565 IIM patients (1). Clinical subgroup analysis was conducted on PM (n = 903), DM (n = 817), juvenile DM (n = 508), and IBM (n = 252) patients, and patients with anti–Jo‐1 autoantibodies (n = 311). Healthy control samples were collected from the same pool of controls but matched with patients 4:1 based on principal components analysis coordinates (9). Variants were imputed with the Michigan Imputation Server using the Haplotype Reference Consortium (HRC) panel version r1.1 2016. The HRC consists of 64,940 haplotypes from individuals of predominantly European ancestry. Poorly imputed genotypes (r^2^ < 0.5), single‐nucleotide polymorphisms (SNPs) deviating from Hardy‐Weinberg equilibrium in controls (*P* < 0.001), and variants with a low minor allele frequency of <0.01 were removed. After stringent SNP quality control, we analyzed 1,648,116 variants, of which 120,734 were directly genotyped SNPs. Population stratification was assessed by the genomic inflation factor (λ) scaled to 1,000 patients and 1,000 controls. Including the top 3 principal components as covariates was sufficient to control for population differences (λ for 1,000 samples = 1.04).

### Statistical analysis

Association analysis was conducted on gene dosages using SNPTest version 2.5.2. Linkage disequilibrium between SNPs was calculated using Plink version 1.90. The first 3 principal components were included as covariates and used in a logistic regression analysis using an additive model. Forward stepwise logistic regression was used to test for independent effects conditional on the variant of interest. Genome‐wide significance was defined as a *P* value less than 5 × 10^−8^. Suggestive significance was defined as a *P* value less than 2.25 × 10^−5^, based on Bonferroni correction for the number of independent haplotype blocks on the original genotyping array. Regions were defined as novel if there was no genome‐wide or suggestive evidence of association in previous genetic analyses. Regional association plots were generated using LocusZoom.js (10).

### Functional analysis

We reported the functional effect of the most strongly associated SNP in the locus from the dbSNP database's predicted functional effect. GARFIELD (GWAS Analysis of Regulatory and Functional Information Enrichment with Linkage Disequilibrium correction) analysis was used to characterize the cellular and regulatory contribution of the associated variants (11). The “gwas‐credible‐sets” package implemented in LocusZoom.js was used to calculate 95% credible SNP sets (10), which were annotated with functional information from public databases, including Genotype‐Tissue Expression (GTEx) and splicing quantitative trait locus (QTL), and regulatory information from the UCSC Genome Browser.

## RESULTS

**Figure 1:**
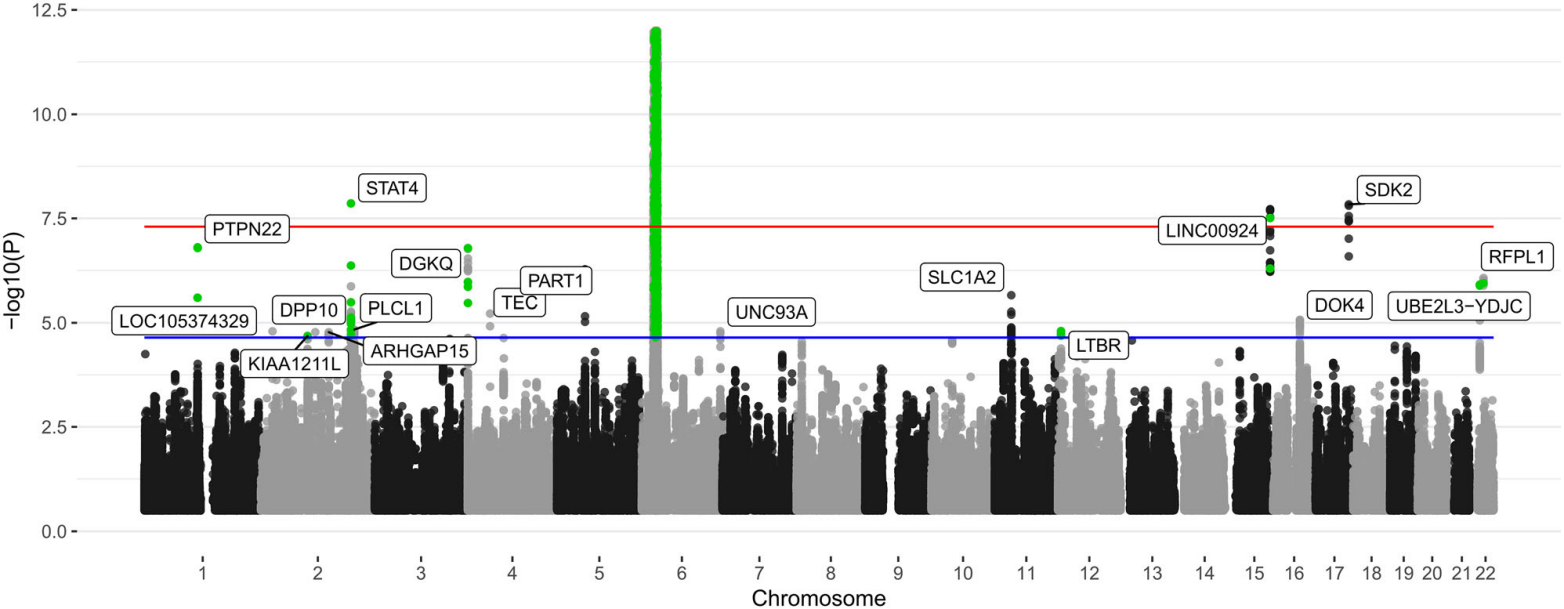
Manhattan plot of the total idiopathic inflammatory myopathy (n = 2,565) association analysis. The red line represents genome‐wide level of significance (*P* < 5 × 10^−8^); the blue line represents suggestive significance calculated from prior coverage from the ImmunoChip array (*P* < 2.25 × 10^−5^). Single‐nucleotide polymorphisms reaching *P* < 2.25 × 10^−5^ that were directly genotyped are colored green to differentiate from imputed variants. For visualization purposes, the y‐axis has a cutoff in the HLA region (chromosome 6, 25–35 Mb) of *P* < 1 × 10^−1^.

Regions of interest associated with IIM and clinical subgroups are presented in Table 1 and Figure 1. SNPs are reported if they reached genome‐wide significance, or reached suggestive significance and the locus has previous statistical evidence of association in an autoimmune rheumatic disease in the National Human Genome Research Institute–European Bioinformatics Institute GWAS Catalog. All associations reaching a suggestive significance threshold of *P* < 2.25 × 10^−5^ are included in the Supplementary Data, available on the *Arthritis & Rheumatology* website at https://onlinelibrary.wiley.com/doi/10.1002/art.42434. A Manhattan plot of the combined IIM analysis is shown in Figure 1. Manhattan plots for subgroup analyses and regional association plots for non‐HLA associations are included in Supplementary Figures 1–19, https://onlinelibrary.wiley.com/doi/10.1002/art.42434.

**Table 1:** Loci associated with IIM reaching genome‐wide significance or suggestive significance compared to matched healthy controls*

Gene region	Subgroup	SNP	Chromosome	Position	Minor Allele	MAF in Patients	MAF in Controls	*P*	OR (95% CI)	Function†	Overlap
*HLA–DRA*	IIM	rs9268813	6	32424594	C	0.24	0.11	6.69 × 10^−120^	2.49 (2.30–2.69)	–	Multiple
*STAT4* ‡	IIM	rs4853540	2	191917317	T	0.19	0.22	1.38 × 10^−8^	0.81 (0.75–0.87)	Intronic	RA (ref. 14), SLE (ref. 15), SSc (ref. 16)
*SDK2*	IIM	rs7209879	17	71532097	T	0.37	0.34	1.46 × 10^−8^	1.15 (1.08–1.23)	Intronic	–
*LINC00924*	IIM	rs8040452	15	96197257	T	0.37	0.42	1.9 × 10^−8^	0.84 (0.79–0.89)	Intergenic	–
*PHTF1‐PTPN22*	PM	rs6679677	1	114303808	A	0.12	0.1	1.57 × 10^−7^	1.3 (1.19–1.43)	Exonic	RA (ref. 14), SLE (ref. 15)
*DGKQ*	IIM	rs6599390	4	956047	A	0.3	0.34	1.64 × 10^−7^	0.84 (0.78–0.89)	Intronic	Seropositive RD (ref. 2), SSc (ref. 16)
*UBE2L3‐YDJC*	IIM	rs11089637	22	21979096	C	0.19	0.16	1.23 × 10^−6^	1.23 (1.13–1.33)	Intergenic	RA (ref. 14), JIA (ref. 13), SLE (ref. 15)
*TEC*	IIM	rs80105690	4	48155618	T	0.09	0.07	6 × 10^−6^	1.27 (1.14–1.42)	Intronic	RA (ref. 14)
*PLCL1*	IIM	rs1518359	2	198847383	T	0.52	0.49	1.45 × 10^−5^	1.14 (1.07–1.21)	Intronic	SLE (ref. 17)
*LTBR*	IIM	rs11064180	12	6523249	T	0.38	0.41	1.58 × 10^−5^	0.87 (0.81–0.92)	Intergenic	JIA (ref. 13), AS (ref. 12)
*HLA–DQB1*	PM	rs3129716	6	32657436	C	0.29	0.13	1.54 × 10^−54^	2.65 (2.35–3.00)	–	Multiple
*NAB1*	PM	rs6733720	2	191516020	G	0.22	0.17	1.96 × 10^−8^	1.41 (1.24–1.60)	Intronic	Seropositive RD (ref. 2), SSc (ref. 16)
*PTPN22*	PM	rs2476601	1	114377568	A	0.15	0.1	1.25 × 10^−6^	1.46 (1.26–1.70)	Exonic	RA (ref. 14), SLE (ref. 15)
*FAM167A‐BLK*	PM	rs17799348	8	11333521	T	0.33	0.39	1.72 × 10^−6^	0.77 (0.69–0.85)	Intergenic	Seropositive RD (ref. 2), SSc (ref. 16), RA (ref. 14), SLE (ref. 15)
*HLA–B*	DM	rs7748141	6	31288877	C	0.23	0.12	2.70 × 10^−28^	2.17 (1.89–2.49)	–	Multiple
*HLA–DRA*	JDM	rs12204922	6	32451613	C	0.3	0.18	7.99 × 10^−16^	1.91 (1.63–2.23)	–	Multiple
*HLA–DQB1*	IBM	rs4713570	6	32626040	T	0.57	0.24	3.9 × 10^−45^	4.15 (3.39–5.09)	–	Multiple
*CCR5*	IBM	rs41490645	3	46410137	C	0.08	0.16	5.96 × 10^−7^	0.45 (0.32–0.63)	Upstream Variant	JIA (ref. 18)
*HLA–DRA*	Jo‐1	rs9268813	6	32424594	C	0.39	0.11	5.01 × 10^−65^	5.15 (4.21–6.32)	–	Multiple

* Genome‐wide significance was defined as *P* < 5 × 10^−8^; suggestive significance was defined as *P* < 2.25 × 10^−5^ with previous statistical evidence of association in an autoimmune rheumatic disease (RD) in the National Human Genome Research Institute–European Bioinformatics Institute Genome‐Wide Association Study Catalog. IIM = idiopathic inflammatory myopathy; SNP = single‐nucleotide polymorphisms; MAF = minor allele frequency; OR = odds ratio; 95% CI = 95% confidence interval; RA = rheumatoid arthritis; SLE = systemic lupus erythematosus; SSc = systemic sclerosis; PM = polymyositis; JIA = juvenile idiopathic arthritis; AS = ankylosing spondylitis; DM = dermatomyositis; JDM = juvenile dermatomyositis; IBM = inclusion body myositis.

† Function obtained from the dbSNP database's predicted functional effect.

‡ Locus with evidence of independent effects.

For IIM as a whole and all clinical subgroups, the HLA region was the most significant genetic risk factor. Three non‐HLA regions, *STAT4* (*P* = 1.38 × 10^−8^, odds ratio [OR] 0.81 [95% confidence interval (95% CI) 0.75–0.87]), *SDK2* (*P =* 1.46 × 10^−8^, OR 1.15 [95% CI 1.08–1.23]), and *LINC00924* (*P* = 1.9 × 10^−8^, OR 0.84 [95% CI 0.79–0.89]), reached genome‐wide significance in the total IIM cohort, and 1 non‐HLA region, *NAB1* (*P* = 1.96 × 10^−8^, OR 1.41 [95% CI 1.24–1.60]), reached genome‐wide significance in the PM subgroup. This is the first time these loci have been found to reach genome‐wide significance in IIM. After conditioning on the SNP with the lowest *P* value in these regions, there were no additional independent effects reaching genome‐wide significance. However, an independent intronic variant in *STAT4* remained suggestively significant in IIM (rs6752770, *P* = 6.1 × 10^−6^, OR 1.11 [95% CI 1.04–1.19]) (Supplementary Figure 7, https://onlinelibrary.wiley.com/doi/10.1002/art.42434).

The 95% credible SNP sets for the 4 regions reaching genome‐wide significance are included in the Supplementary Data (https://onlinelibrary.wiley.com/doi/10.1002/art.42434), along with predicted deleteriousness and functionality using Combined Annotation Dependent Depletion (CADD) and RegulomeDB, respectively. For the *STAT4* and *NAB1* regions, a substantial proportion of the 95% posterior probability for credible SNPs could be attributed to a single SNP; rs4853540 (*STAT4*) maps to an enhancer for *STAT1*, and rs6733720 (*NAB1*) is a significant splicing QTL for *NAB1* in 26 tissues (*P* < 1.6 × 10^−6^) and a significant expression QTL for several genes, including *NAB1* and *GLS* (glutaminase), in multiple tissues (see Supplementary Data).

The *LINC00924* locus is a novel association in autoimmune disease, reaching genome‐wide significance. Most variants in these regions were imputed, explaining the lack of association in our prior IIM ImmunoChip study, where the original SNP may have been removed during quality control (1). Notably, we also found suggestive evidence of association with *TEC* and *LTBR*, which have previously been associated with autoimmune rheumatic diseases (12, 13, 14). Associations with the *STAT4* and *DGKQ* loci were more significant in the current study than in the prior IIM ImmunoChip study, as were associations previously reported in IIM clinical subgroups, such as *NAB1* and *FAM167A‐BLK* loci in PM and *CCR5* in IBM (Table 1).

We used GARFIELD to assess whether IIM‐associated variants are enriched in regulatory elements of specific cell types (11). We found enrichment of variants among DNase I hypersensitivity sites and histone marks associated with active transcription within blood cells (Supplementary Figure 20 and Supplementary Data, https://onlinelibrary.wiley.com/doi/10.1002/art.42434). Specifically, IIM variants were most enriched within DNase I hypersensitivity hotspots of primary CD19+ B cells (*P* = 1.1 × 10^−16^) and CD3+ T cells (*P* = 3 × 10^−15^).

## DISCUSSION

By using imputation to identify and fine‐map genetic associations in IIM, we found 3 new genome‐wide associations in the combined IIM cohort, *STAT4*, *SDK2*, and *LINC00924*. Conditional analysis revealed evidence of independent associations in the *STAT4* region. For the whole IIM group and clinical subgroups, the HLA region was the most significant genetic risk factor. The strongest genetic risk in this region was for the anti–Jo‐1 subgroup despite a sample size of only 331 patients. In the PM subgroup, we found 1 novel genome‐wide association with *NAB1*. This is the first time this data set has been used for genome‐wide imputation, and the first time this data set has been stratified by adult‐ and juvenile‐onset myositis subgroups and by individuals with anti–Jo‐1 autoantibodies.

We defined an associated region by proximity to the nearest gene. For example, in the *LINC00924* region, the strongest association was intergenic, lying approximately 150 kb from this long intergenic noncoding RNA, which has been associated with a number of traits such as coronary heart disease and ischemic stroke. However, it may be that these associations are influencing a different gene or regulatory element lying further away from the most associated SNP. It is worth noting that the lead variant in the *LINC00924* locus was removed during quality control in our prior ImmunoChip analysis. In other instances, such as in the *SDK2* region, the most associated variants lie intronic of the gene. *SDK2* is a member of the immunoglobulin superfamily, although the specific function is not yet known. In both instances, there is no previous evidence of genetic association with rheumatic diseases. A limitation of this study is the lack of a replication cohort due to the rarity of IIM, although patient recruitment is ongoing within the MYOGEN consortium.

For some regions, we found suggestive novel associations that have prior evidence of association with autoimmune disease, such as *TEC*, a tyrosine kinase involved in T cell signalling and activation, and *LTBR*, a signalling receptor expressed on myeloid cells. In other instances, we strengthened previously observed associations with IIM (*STAT4* and *DGKQ*). In addition, it is interesting to note that we observed a suggestive association with *PLCL1* (*P* = 1.45 × 10^−5^, OR 1.14 [95% CI 1.07–1.21]) in the combined IIM analysis. Variants in *PLCL1*, a gene encoding the intracellular signalling molecule phospholipase C–like 1, were previously reported in the MYOGEN DM GWAS, although it was not identified in the subsequent ImmunoChip analysis. We have identified more significant associations than were previously reported in IIM clinical subgroups, such as *NAB1* and *FAM167A‐BLK* loci in PM and *CCR5* in IBM, which also have prior evidence of association with rheumatic disease. Although this study comprised the same cohort as previous studies, this analysis can be viewed as a fine‐mapping experiment; imputation from a large reference panel allows better coverage to localize further signals. Indeed, we found that many associations could be localized to single genes or credible SNPs with high posterior probability, likely due to the coverage of coding genes on the ImmunoChip array resulting in high‐quality imputation. We note that the ImmunoChip is a targeted array and therefore does not have genome‐wide imputation coverage.

Functional analysis of variants reaching suggestive significance in IIM was conducted using GARFIELD. This method uses functional annotation from primary tissues and cell lines from the ENCODE and Roadmap Epigenomics projects. As expected from data generated using the ImmunoChip array, associated variants in IIM were enriched in regulatory elements of blood cells. In particular, the strongest relative enrichment was seen in regions of open chromatin in CD19+ B cells and CD3+ T cells. The role of T and B cells is well known in IIM through muscle immunohistopathology and the presence of autoantibodies, and our findings suggest their contribution to disease pathology may be genetically encoded.

Increasing the number of patient samples in an analysis should statistically strengthen suggestive associations from previous studies. A recent genome‐wide meta‐analysis of 4 seropositive rheumatic diseases revealed several novel loci, for example, *NAB1*, *DGKQ*, and *YDJC*, where IIM contributed to the association. Using additional IIM patients to those included in the meta‐analysis, we were able to identify these associations in IIM only, and in some cases, such as *NAB1* in PM, attribute these associations to specific clinical subgroups of IIM. The lead variant rs6733720 in *NAB1* is a significant splicing QTL for *NAB1* in 26 tissues and an expression QTL for several genes, including *NAB1* and *GLS*, in different tissues. These *NAB1* variants are also in moderate linkage disequilibrium (r^2^ = 0.66), with a variant previously identified in systemic sclerosis and rheumatoid arthritis, which acts as an expression QTL for *NAB1* expression in lymphoblastoid cell lines (2).

Although we found a number of interesting associations in the PM subgroup, one limitation of these findings is the potential heterogeneity within this subgroup. The Bohan and Peter criteria do not differentiate between PM and immune‐mediated necrotizing myopathy. In addition, we included patients classified as having PM that have autoantibodies against transfer RNA synthetases, although antisynthetase syndrome is increasingly recognised as a separate entity. Some previous genetic studies in IIM combined adult‐ and juvenile‐onset DM for analysis. This study stratified DM by age of onset to investigate non‐HLA associations. However, there do not seem to be strong signals that differentiate these clinical subgroups. Our previous work has shown that stratifying IIM cohorts by autoantibody status may increase power to detect genetic associations within the HLA region (9). Therefore, we also analyzed a subgroup of patients with anti–Jo‐1 autoantibodies; however, we did not find any significant associations outside the HLA region. Although the anti–Jo‐1 subgroup is thought to be more clinically homogeneous, there may have been a lack of power to detect associations with only 331 patients in the analysis. For this reason, we did not investigate genetic associations with other less common autoantibodies.

To our knowledge, this is the largest genetic association study investigating non‐HLA genes in patients with anti–Jo‐1 autoantibodies. A recent study targeted a number of SNPs in the *IL1B* locus in a Mexican cohort of 154 antisynthetase‐positive IIM patients and found an association with a synonymous SNP in *IL1B* (19). We could not replicate this association in our Caucasian cohort (rs1143634, *P* = 0.1). In the prior ImmunoChip analysis, *PTPN22* was the only non‐HLA region to reach genome‐wide significance; however, in this analysis, *PTPN22* did not reach genome‐wide significance. This disparity may be due to the more accurate matching of patients to controls, as it is known that there is a wide variation of allele frequency among different European populations of the *PTPN22* R620W risk polymorphism (20).

In summary, we used imputation to identify and fine‐map genetic associations in IIM. We found 4 new genome‐wide associations in IIM and PM, and we found associations reaching suggestive significance that have previously been associated with autoimmune rheumatic disease. These risk variants are functionally enriched in relevant immune cells, expanding our knowledge of the genetic architecture of IIM.

## AUTHOR CONTRIBUTIONS

All authors were involved in drafting the article or revising it critically for important intellectual content, and all authors approved the final version to be published. Dr. Rothwell had full access to all of the data in the study and takes responsibility for the integrity of the data and the accuracy of the data analysis.

### Study conception and design

Rothwell, Amos, Miller, Rider, Lundberg, Wedderburn, Chinoy, Lamb.

### Acquisition of data

Rothwell, Miller, Rider, Lundberg, Gregersen, Vencovsky, McHugh, Limaye, Selva‐O'Callaghan, Hanna, Machado, Pachman, Reed, Molberg, Benveniste, Mathiesen, Radstake, Doria, De Bleecker, De Paepe, Maurer, Ollier, Padyukov, O'Hanlon, Lee, Wedderburn, Chinoy, Lamb.

### Analysis and interpretation of data

Rothwell, Amos, Miller, Rider, Lundberg, McHugh, Ollier, Padyukov, Wedderburn, Chinoy, Lamb.

## Supporting information


Disclosure Form

**Appendix S1:** Supplementary Material

**Data S1:** Supplementary Data

**Figure S1:** Supplementary Figure
