# Supplementary material for: Identification of Novel Associations and Localization of Signals in Idiopathic Inflammatory Myopathies Using Genome‐Wide Imputation
Source: Arthritis Rheumatol. 2023 Mar 20;75(6):1021–7. doi: 10.1002/art.42434 (PMC10238560; doi:10.1002/art.42434)
Supplement: Supplementary file 2 — Appendix S1: Supplementary Material [file ART-75-1021-s002.docx]

**Supplementary Material**

Myositis Genetics Consortium (MYOGEN) members who contributed to the present study

Study investigators of the Myositis Genetics Consortium, in addition to the authors of this article, are as follows: Robert G. Cooper, Katalin Danko (Deceased), Christopher Denton (Royal Free Hospital, London, UK), Herman Mann (Institute of Rheumatology, Prague), David Hilton-Jones (John Radcliffe Hospital, Oxford, UK), Patrick Kiely (St. George's Hospital, London, UK), Paul H. Plotz, Mark Gourley (National Institute of Arthritis and Musculoskeletal and Skin Diseases, National Institutes of Health, Bethesda, MD), Kelly Rouster-Stevens (Emory University School of Medicine, Atlanta, GA), Adam M Huber (Dalhousie University, Halifax, Nova Scotia, Canada), Galina Marder (North Shore University Hospital, Great Neck, NY), Mazen Dimachkie and Richard J. Barohn (University of Kansas Medical Center, Kansas City, KS).

Shared Immunochip Control Cohorts

We thank the Rheumatoid Arthritis Consortium International (RACI) for Netherlands, Spanish, Swedish, UK and US Immunochip control genotypes. This study makes use of data generated by the Wellcome Trust Case-Control Consortium. A full list of the investigators who contributed to the generation of the data is available from [www.wtccc.org.uk](file:///C:\Documents%20and%20Settings\mdehssrc\Local%20Settings\Temporary%20Internet%20Files\Content.Outlook\L1ASXN82\www.wtccc.org.uk). Funding for the project was provided by the Wellcome Trust under award 076113 and 085475. Swedish control data was provided from EIRA study, Professor Lars Alfredsson, Department of Environmental Medicine, Karolinska Institutet, Stockholm, Sweden. Control data from the Netherlands was provided from the department of Rheumatology, Leiden University Medical Center, Leiden, The Netherlands.

Polish control data was provided by the Celiac Disease Consortium and Hungarian control data collected with the help of the Hungarian Research Fund (OTKA) grant K101788 was provided by Prof Ilma Korponay-Szabo, Celiac Disease Centre, Heim Pál Children's Hospital, Budapest and University of Debrecen, Debrecen Hungary.

We acknowledge the International MS Genetics Consortium for providing access to control sample data from Belgium, France, Norway, Italy and Germany. The collection and genotyping of these samples was made possible by: the Norwegian MS society and the Norwegian Bone Marrow Registry (Norwegian samples); the French Biological Resource Center for MS Genetics, Genethon and INSERM (French samples); and a FISM (Italian Foundation for Multiple Sclerosis) grant (“Progetto Speciale Immunochip”) for Italian samples.

Italian samples were collected by Prof. Sandra D'Alfonso (Interdisciplinary Research Center of Autoimmune Diseases IRCAD, University of Eastern Piedmont, Novara, Italy; PROGEMUS Consortium) and Dr. Martinelli Boneschi (Laboratory of Genetics of Complex Neurological Disorders, Division of Neuroscience & INSPE, San Raffaele Scientific Institute, Milan, Italy; PROGRESSO Consortium); funding was provided by a FISM (Italian Foundation for Multiple Sclerosis) grant (“Progetto Speciale Immunochip). The KORA study was initiated and financed by the Helmholtz Zentrum München – German Research Center for Environmental Health, which is funded by the German Federal Ministry of Education and Research (BMBF) and by the State of Bavaria. Furthermore, KORA research was supported within the Munich Center of Health Sciences (MC-Health), Ludwig-Maximilians-Universität, as part of LMUinnovativ.

UK Adult Onset Myositis Immunogenetic Collaboration (UKMYONET)

Members of the UK Adult Onset Myositis Immunogenetic Collaboration who recruited and enrolled subjects are as follows: Drs. Yasmeen Ahmed (Llandudno General Hospital), Raymond Armstrong (Southampton General Hospital), Robert Bernstein (Manchester Royal Infirmary), Carol Black (Royal Free Hospital, London), Simon Bowman (University Hospital, Birmingham), Ian Bruce (Manchester Royal Infirmary), Robin Butler (Robert Jones & Agnes Hunt Orthopaedic Hospital, Oswestry), John Carty (Lincoln County Hospital), Chandra Chattopadhyay (Wrightington Hospital), Easwaradhas Chelliah (Wrightington Hospital), Fiona Clarke (James Cook University Hospital, Middlesborough), Peter Dawes (Staffordshire Rheumatology Centre, Stoke on Trent), Joseph Devlin (Pinderfields General Hospital, Wakefield), Christopher Edwards (Southampton General Hospital), Paul Emery (Academic Unit of Musculoskeletal Disease, Leeds), John Fordham (South Cleveland Hospital, Middlesborough), Alexander Fraser (Academic Unit of Musculoskeletal Disease, Leeds), Hill Gaston (Addenbrooke's Hospital, Cambridge), Patrick Gordon (King's College Hospital, London), Bridget Griffiths (Freeman Hospital, Newcastle), Harsha Gunawardena (Frenchay Hospital, Bristol), Frances Hall (Addenbrooke's Hospital, Cambridge), Beverley Harrison (North Manchester General Hospital), Elaine Hay (Staffordshire Rheumatology Centre, Stoke on Trent), Lesley Horden (Dewsbury District General Hospital), John Isaacs (Freeman Hospital, Newcastle), Adrian Jones (Nottingham University Hospital), Sanjeet Kamath (Staffordshire Rheumatology Centre, Stoke on Trent), Thomas Kennedy (Royal Liverpool Hospital), George Kitas (Dudley Group Hospitals Trust, Birmingham), Peter Klimiuk (Royal Oldham Hospital), Sally Knights (Yeovil District Hospital, Somerset), John Lambert (Doncaster Royal Infirmary), Peter Lanyon (Queen's Medical Centre, Nottingham), Ramasharan Laxminarayan (Queen's Hospital, Burton Upon Trent), Bryan Lecky (Walton Neuroscience Centre, Liverpool), Raashid Luqmani (Nuffield Orthopaedic Centre, Oxford), Jeffrey Marks (Steeping Hill Hospital, Stockport), Michael Martin (St. James University Hospital, Leeds), Dennis McGonagle (Academic Unit of Musculoskeletal Disease, Leeds), Neil McHugh (Royal National Hospital for Rheumatic Diseases, Bath), Francis McKenna (Trafford General Hospital, Manchester), John McLaren (Cameron Hospital, Fife), Michael McMahon (Dumfries & Galloway Royal Infirmary, Dumfries), Euan McRorie (Western General Hospital, Edinburgh), Peter Merry (Norfolk & Norwich University Hospital, Norwich), Sarah Miles (Dewsbury & District General Hospital, Dewsbury), James Miller (Royal Victoria Hospital, Newcastle), Anne Nicholls (West Suffolk Hospital, Bury St. Edmunds), Jennifer Nixon (Countess of Chester Hospital, Chester), Voon Ong (Royal Free Hospital, London), Katherine Over (Countess of Chester Hospital, Chester), John Packham (Staffordshire Rheumatology Centre, Stoke on Trent), Nicolo Pipitone (King's College Hospital, London), Michael Plant (South Cleveland Hospital, Middlesborough), Gillian Pountain (Hinchingbrooke Hospital, Huntington), Thomas Pullar (Ninewells Hospital, Dundee), Mark Roberts (Salford Royal Foundation Trust), Paul Sanders (Wythenshawe Hospital, Manchester), David Scott (King's College Hospital, London), David Scott (Norfolk & Norwich University Hospital, Norwich), Michael Shadforth (Staffordshire Rheumatology Centre, Stoke on Trent), Thomas Sheeran (Cannock Chase Hospital, Cannock, Staffordshire), Arul Srinivasan (Broomfield Hospital, Chelmsford), David Swinson (Wrightington Hospital), Lee-Suan Teh (Royal Blackburn Hospital, Blackburn), Michael Webley (Stoke Manderville Hospital, Aylesbury), Brian Williams (University Hospital of Wales, Cardiff), and Jonathan Winer (Queen Elizabeth Hospital, Birmingham).

Juvenile Dermatomyositis Cohort Biomarker Study and Repository (JDCBS)

The Juvenile Dermatomyositis Research Group would like to thank all of the patients and their families who contributed to the Juvenile Dermatomyositis Cohort & Biomarker Study & Repository. We thank all local research coordinators and principal investigators who have made this research possible. The JDRG members were as follows: Dr Kate Armon, and Ms Louise Coke (Norfolk and Norwich University Hospitals);Dr Liza McCann, Mr Ian Roberts, Dr Eileen Baildam, Ms Louise Hanna, Ms Olivia Lloyd, Susan Wadeson and Ms Michelle Andrews (The Royal Liverpool Children’s Hospital, Alder Hey, Liverpool); Dr Phil Riley, Ms Ann McGovern, and Ms Verna Cuthbert (Royal Manchester Children’s Hospital, Manchester); Dr Clive Ryder, Ms Janis Scott, Ms Beverley Thomas, Professor Taunton Southwood, Dr Eslam Al-Abadi and Ms Ruth Howman (Birmingham Children’s Hospital, Birmingham); Dr Sue Wyatt, Mrs Gillian Jackson, Dr Mark Wood, Dr Tania Amin, Dr Vanessa VanRooyen, Ms Deborah Burton, Ms Louise Turner, Ms Heather Rostron, and Ms Sarah Hanson (Leeds General Infirmary, Leeds); Dr Joyce Davidson, Dr Janet Gardner-Medwin, Dr Neil Martin, Ms Sue Ferguson, Ms Liz Waxman and Mr Michael Browne, Ms Roisin Boyle, and Ms Emily Blyth (The Royal Hospital for Sick Children, Yorkhill, Glasgow); Dr Mark Friswell, Professor Helen Foster, Ms Alison Swift, Dr Sharmila Jandial, Ms Vicky Stevenson, Ms Debbie Wade, Dr Ethan Sen, Dr Eve Smith, Ms Lisa Qiao, Mr Stuart Watson and Ms Claire Duong (Great North Children’s Hospital, Newcastle); Dr Helen Venning, Dr Rangaraj Satyapal, Mrs Elizabeth Stretton, Ms Mary Jordan, Dr Ellen Mosley, Ms Anna Frost, Ms Lindsay Crate, Dr Kishore Warrier, and Ms Stefanie Stafford, (Queens Medical Centre, Nottingham); Professor Lucy Wedderburn, Dr Clarissa Pilkington, Dr Nathan Hasson, Dr Muthana Al-Obadi, Dr Giulia Varnier, Dr Sandrine Lacassagne, Ms Sue Maillard, Mrs Lauren Stone, Ms Elizabeth Halkon, Ms Virginia Brown, Ms Audrey Juggins, Dr Sally Smith, Ms Sian Lunt, Ms Elli Enayat, Ms Hemlata Varsani, Ms Laura Kassoumeri, Miss Laura Beard, Ms Katie Arnold, Mrs Yvonne Glackin, Ms Stephanie Simou, Dr Beverley Almeida, Dr Kiran Nistala, Dr Raquel Marques, Dr Claire Deakin, Dr Parichat Khaosut, Ms Stefanie Dowle, Dr Charalampia Papadopoulou, Dr Shireena Yasin, Dr Christina Boros, Dr Meredyth Wilkinson, Dr Chris Piper, Ms Cerise Johnson-Moore, Ms Lucy Marshall, Ms Kathryn O’Brien, Ms Emily Robinson, Mr Dominic Igbelina, Dr Polly Livermore, Dr Socrates Varakliotis, and Ms Rosie Hamilton (Great Ormond Street Hospital, London); Dr Kevin Murray (Princess Margaret Hospital, Perth, Western Australia); Dr Coziana Ciurtin, Dr John Ioannou, Mrs Caitlin Clifford, Ms Linda Suffield and Ms Laura Hennelly (University College London Hospital, London); Ms Helen Lee, Ms Sam Leach, Ms Helen Smith, Dr Anne-Marie McMahon, Ms Heather Chisem, and Ms Jeanette Hall (Sheffield’s Children’s Hospital, Sheffield); Dr Nick Wilkinson, Ms Emma Inness, Ms Eunice Kendall, Mr David Mayers, Ms Ruth Etherton, Ms Danielle Miller and Dr Kathryn Bailey (Oxford University Hospitals, Oxford); Dr Jacqui Clinch, Ms Natalie Fineman, Ms Helen Pluess-Hall, Ms Suzanne Sketchley, and Ms Melanie Marsh (Bristol Royal Hospital for Children, Bristol); Dr Joyce Davidson, Margaret Connon and Ms Lindsay Vallance (Royal Aberdeen Children’s Hospital); Dr Kirsty Haslam, Ms Charlene Bass-Woodcock, Ms Trudy Booth, and Ms Louise Akeroyd (Bradford Teaching Hospitals); Dr Alice Leahy, Amy Collier, Rebecca Cutts, Emma Macleod, Dr Hans De Graaf, Dr Brian Davidson, Sarah Hartfree, Ms Elizabeth Fofana and Ms Lorena Caruana (University Hospital Southampton) and all the Children, Young people and their families who have contributed to this research.

US Childhood Myositis Heterogeneity Study Group

The following members of the US Childhood Myositis Heterogeneity Study Group contributed to this study: Drs. Barbara S. Adams (University of Michigan, Ann Arbor, MI), Catherine A. Bingham (Hershey Medical Center, Hershey, PA), Gail D. Cawkwell (All Children's Hospital, St. Petersburg, FL), Terri H. Finkel (Children's Hospital of Philadelphia, Philadelphia, PA), Steven W. George (Ellicott City, MD), Harry L. Gewanter (Richmond, VA), Ellen A. Goldmuntz (Children's National Medical Center, Washington, DC), Donald P. Goldsmith (St. Christopher's Hospital for Children, Philadelphia, PA), Michael Henrickson (Children's Hospital, Madera, CA), Lisa Imundo (Columbia University, New York, NY), Ildy M. Katona (Uniformed Services University, Bethesda, MD), Carol B. Lindsley (University of Kansas, Kansas City), Chester P. Oddis (University of Pittsburgh, Pittsburgh, PA), Judyann C. Olson (Medical College of Wisconsin, Milwaukee), David Sherry (Children's Hospital of Philadelphia, Philadelphia, PA), Scott A. Vogelgesang (Walter Reed Army Medical Center, Washington, DC), Carol A. Wallace (Children's Medical Center, Seattle, WA), Patience H. White (George Washington University, Washington, DC), and Lawrence S. Zemel (Connecticut Children's Hospital, Hartford).
