# Supplementary material for: Identification of Novel Associations and Localization of Signals in Idiopathic Inflammatory Myopathies Using Genome‐Wide Imputation
Source: Arthritis Rheumatol. 2023 Mar 20;75(6):1021–7. doi: 10.1002/art.42434 (PMC10238560; doi:10.1002/art.42434)
Supplement: Supplementary file 3 — Data S1: Supplementary Data [file ART-75-1021-s004.pdf]

Associations in IIM and asociated clinical subgroups reaching a suggestive significance threshold of p<2.25x10-5

Subgroup	SNP	Chr.	Position	Minor Allele	MAF Cases	MAF Controls	P-value	OR (95% CI)	Nearest Gene
IIM	rs6679677	1	114303808	A	0.12	0.10	1.57E-07	1.30 (1.19-1.43)	*PHTF1*
IIM	rs12623207	2	24693470	G	0.25	0.22	1.60E-05	1.13 (1.05-1.21)	*NCOA1*
IIM	rs10189330	2	99389870	T	0.50	0.47	2.07E-05	1.16 (1.09-1.23)	*MGAT4A*
IIM	rs4289207	2	115870105	T	0.45	0.42	1.68E-05	1.13 (1.06-1.20)	*DPP10*
IIM	rs10210556	2	144575729	T	0.42	0.45	1.68E-05	0.90 (0.85-0.96)	*GTDC1*
IIM	rs4853540	2	191917317	T	0.19	0.22	1.38E-08	0.81 (0.75-0.87)	*STAT4*
IIM	rs1518359	2	198847383	T	0.52	0.49	1.45E-05	1.14 (1.07-1.21)	*PLCL1*
IIM	rs6599390	4	956047	A	0.30	0.34	1.64E-07	0.84 (0.78-0.89)	*DGKQ*
IIM	rs80105690	4	48155618	T	0.09	0.07	6.00E-06	1.27 (1.14-1.42)	*TEC*
IIM	rs456009	5	59796734	A	0.37	0.41	5.29E-07	0.87 (0.82-0.93)	*PDE4D*
IIM	rs9268813	6	32424594	C	0.24	0.11	6.69E-120	2.49 (2.30-2.69)	*HLA-DRA*
IIM	rs1555031	6	167708818	A	0.08	0.09	1.60E-05	0.79 (0.71-0.89)	*UNC93A*
IIM	rs3794087	11	35329615	T	0.20	0.22	2.22E-06	0.86 (0.80-0.93)	*SLC1A2*
IIM	rs11064180	12	6523249	T	0.38	0.41	1.58E-05	0.87 (0.81-0.92)	*LTBR*
IIM	rs8040452	15	96197257	T	0.37	0.42	1.90E-08	0.84 (0.79-0.89)	*LINC00924*
IIM	rs623685	16	57515188	C	0.33	0.31	8.46E-06	1.13 (1.06-1.21)	*DOK4*
IIM	rs7209879	17	71532097	T	0.37	0.34	1.46E-08	1.15 (1.08-1.23)	*SDK2*
IIM	rs11089637	22	21979096	C	0.19	0.16	1.23E-06	1.23 (1.13-1.33)	*YDJC*
IIM	rs6006145	22	29825404	C	0.02	0.01	8.53E-07	2.05 (1.58-2.66)	*AP1B1*
PM	rs2476601	1	114377568	A	0.15	0.10	1.25E-06	1.46 (1.26-1.70)	*PTPN22*
PM	rs114798451	1	163323881	C	0.01	0.02	1.79E-05	0.47 (0.27-0.82)	*NUF2*
PM	rs6733720	2	191516020	G	0.22	0.17	1.96E-08	1.41 (1.24-1.60)	*NAB1*
PM	rs3129716	6	32657436	C	0.29	0.13	1.54E-54	2.65 (2.35-3.00)	*HLA-DQB1*
PM	rs1369496	7	12173173	G	0.18	0.22	2.23E-06	0.78 (0.69-0.90)	*TMEM106B*
PM	rs17799348	8	11333521	T	0.33	0.39	1.72E-06	0.77 (0.69-0.85)	*FAM167A*
PM	rs2241208	12	109962795	G	0.41	0.47	2.42E-06	0.78 (0.70-0.87)	*UBE3B*
PM	rs12590856	14	56654346	A	0.09	0.13	1.18E-05	0.69 (0.58-0.82)	*PELI2*
PM	rs7211759	17	71529176	A	0.43	0.38	4.97E-06	1.25 (1.12-1.39)	*SDK2*
DM	rs1428981	5	4648027	C	0.41	0.45	1.90E-05	0.85 (0.76-0.94)	*ADAMTS16*
DM	rs7748141	6	31288877	C	0.23	0.12	2.70E-28	2.17 (1.89-2.49)	*HLA-B*
DM	rs62042594	16	58419664	T	0.14	0.18	1.21E-06	0.77 (0.66-0.90)	*GINS3*
DM	rs564724656	22	21986860	G	0.02	0.01	2.19E-05	2.41 (1.55-3.73)	*CCDC116*
JDM	rs76771408	2	182166237	A	0.03	0.05	1.94E-05	0.50 (0.33-0.75)	*ITGA4*
JDM	rs79096446	5	149828636	G	0.01	0.02	1.88E-05	0.28 (0.12-0.63)	*RPS14*
JDM	rs12204922	6	32451613	C	0.30	0.18	7.99E-16	1.91 (1.63-2.23)	*HLA-DRA*
JDM	rs142888721	12	57264127	A	0.04	0.02	1.73E-05	2.05 (1.38-3.04)	*SDR9C7*
JDM	rs437742	13	93744376	C	0.43	0.50	1.90E-05	0.77 (0.67-0.88)	*GPC6*
JDM	rs113703584	19	936297	A	0.09	0.13	8.91E-06	0.67 (0.54-0.85)	*ARID3A*
IBM	rs203797	1	167819529	G	0.16	0.10	1.47E-05	1.61 (1.21-2.12)	*ADCY10*
IBM	rs11117933	1	217957394	A	0.49	0.39	1.04E-05	1.48 (1.22-1.80)	*SPATA17*
IBM	rs78158108	2	21296537	C	0.11	0.06	1.44E-05	1.87 (1.35-2.60)	*APOB*
IBM	rs7568633	2	121322038	C	0.34	0.42	3.30E-06	0.71 (0.58-0.87)	*LINC01101*
IBM	rs41490645	3	46410137	C	0.08	0.16	5.96E-07	0.45 (0.32-0.63)	*CCR5*
IBM	rs9864529	3	188105056	G	0.57	0.46	1.02E-05	1.55 (1.27-1.88)	*LPP*
IBM	rs13172971	5	72581788	G	0.39	0.29	9.82E-06	1.55 (1.27-1.90)	*TMEM174*
IBM	rs9367842	6	14561898	C	0.11	0.06	1.25E-05	2.10 (1.50-2.94)	*CD83*
IBM	rs4713570	6	32626040	T	0.57	0.24	3.90E-45	4.15 (3.39-5.09)	*HLA_DQB1*
IBM	rs12442533	15	45529631	C	0.24	0.31	7.55E-06	0.71 (0.56-0.88)	*SLC28A2*
IBM	rs550730	18	12504244	A	0.12	0.07	1.61E-05	1.80 (1.31-2.48)	*SPIRE1*
Jo-1	rs115636515	1	242929551	A	0.03	0.07	4.05E-06	0.36 (0.22-0.60)	*PLD5*
Jo-1	rs76095831	2	44987177	A	0.09	0.05	1.90E-05	1.93 (1.40-2.67)	*CAMKMT*
Jo-1	rs9268813	6	32424594	C	0.39	0.11	5.01E-65	5.15 (4.21-6.32)	*HLA-DRA*
Jo-1	rs259961	20	57825970	A	0.43	0.35	2.14E-05	1.40 (1.17-1.67)	*ZNF831*

Functional annotation for SNPs with posterior probability >0.1

Subgroup	Gene	RSID	Chrom	Pos	Posterior F	GTEx significant eQTL (v8)#	GTEx sQTL (v8)	ENCODE cCREs	GeneHancer (v4.8)	JASPAR CORE 2022 TFBS ($p < 10^{-4}$, score 0-1000)
Total IIM	STAT4	rs4853540	2	1.92E+08	0.912505	-	No sQTL data	-	Double Elite Enhancer for STAT1	Irf1 (432), IRF7 (466)
Total IIM	SDK2	rs7209879	17	71532097	0.253758	-	No sQTL data	-	-	PRDM9 (760), SPIC (427), Spi1 (430), ZSCAN31 (496), ETV5::FIGLA (427), TFAP4::ETV1 (438)
Total IIM	SDK2	rs55902840	17	71532073	0.236168	-	No sQTL data	-	-	RREB1 (474), EGR4 (579), KLF9 (435), GLI3 (592), ZNF76 (459), ZNF143
Total IIM	SDK2	rs12952756	17	71530601	0.135693	SDK2 in whole blood ($p=3.7x10^{-6}$)	No sQTL data	-	-	Wt1 (438), PPARA::RXRA (568)
Total IIM	SDK2	rs1872085	17	71528080	0.11115	-	No sQTL data	-	-	-
Total IIM	SDK2	rs7211759	17	71529176	0.105469	-	No sQTL data	-	-	-
Total IIM	SDK2	rs62070887	17	71529440	0.101763	-	No sQTL data	dELS,CTCF-bound	-	ZNF454 (482), PLAG1 (423), PLAGl1 (452), PLAGL2 (426)
Total IIM	LINC00924	rs8040452	15	96197257	0.137032	RP11-61011.1 in tibial nerve (p=4.8x1	No sQTL data	-	-	PRDM9 (402), ELF3 (454)
Total IIM	LINC00924	rs11858381	15	96196885	0.133266	RP11-61011.1 in tibial nerve (p=4.8x1	No sQTL data	dELS	-	ZNF341 (461), ZNF189 (472), ZBTB12 (427), Stat5a::Stat5b (464), Stat5a (510), STAT1 (430), STAT3 (436), Stat4 (470)
Total IIM	LINC00924	rs2397822	15	96196117	0.132339	RP11-61011.1 in tibial nerve (p=4.8x1	No sQTL data	-	-	Spi1 (421), SPIB (409)
Total IIM	LINC00924	rs8028739	15	96195106	0.124277	RP11-61011.1 in tibial nerve (p=4.8x1	No sQTL data	-	-	TBXT (400)
PM	NAB1	rs6733720	2	1.92E+08	0.285436	RP11-284E5.1 in tibial artery (p=3.4x1	NAB1 in 26 tissues, r	-	Promoter/enhancer	Nkx3-2 (407), Nkx3-1 (427), ZNF418 (411)

#Significance determined by GTEx browser using a Q-value threshold and at least 70 samples per tissue.

ENCODE cCREs: Registry of candidate cis-Regulatory Elements combined from all cell types

dELS: distal enhancer-like signature

95% credible SNP sets for the STAT4 region, with predicted deleteriousness and functionality using CADD and regulomeDB

Subgroup	Gene Locus	rsID	Chrom	Pos	Minor Allele	Cases MAF	ontrols MA	OR	OR Lower	OR Upper	P-value	Credible SNP Set	Posterior Probability	Func	CADD	RDB
Total IIM	STAT4	rs4853540	2	1.92E+08	T	0.185	0.220	0.806	0.746	0.871	1.38E-08	TRUE	0.913	intronic	3.174	5
Total IIM	STAT4	rs7601754	2	1.92E+08	G	0.158	0.187	0.814	0.749	0.884	4.31E-07	TRUE	0.033	intronic	1.589	5
Total IIM	STAT4	rs10931480	2	1.92E+08	G	0.160	0.189	0.820	0.755	0.890	1.36E-06	TRUE	0.011	intronic	3.789	7

95% credible SNP sets for the SDK2 region, with predicted deleteriousness and functionality using CADD and regulomeDB

Subgroup	Gene Locus	rsID	Chrom	Pos	Minor Allele	Cases MAF	ontrols MA	OR	OR Lower	OR Upper	P-value	edible SNP	erior Proba	Func	CADD	RDB
Total IIM	SDK2	rs7209879	17	71532097	T	0.370	0.338	1.152	1.081	1.227	1.46E-08	TRUE	0.254	intronic	3.479	5
Total IIM	SDK2	rs5590284	17	71532073	T	0.377	0.345	1.149	1.078	1.224	1.57E-08	TRUE	0.236	intronic	3.383	5
Total IIM	SDK2	rs1295275	17	71530601	C	0.474	0.432	1.184	1.114	1.259	2.78E-08	TRUE	0.136	intronic	0.337	5
Total IIM	SDK2	rs1872085	17	71528080	G	0.471	0.428	1.189	1.118	1.264	3.42E-08	TRUE	0.111	intronic	0.35	NA
Total IIM	SDK2	rs7211759	17	71529176	A	0.418	0.380	1.174	1.103	1.249	3.60E-08	TRUE	0.105	intronic	1.533	5
Total IIM	SDK2	rs6207088	17	71529440	T	0.420	0.382	1.172	1.102	1.248	3.74E-08	TRUE	0.102	intronic	0.155	5
Total IIM	SDK2	rs748875	17	71519830	A	0.471	0.432	1.171	1.102	1.246	9.72E-08	TRUE	0.040	intronic	7.242	NA

95% credible SNP sets for the LINC00924 region, with predicted deleteriousness and functionality using CADD and regulomeDB

Subgroup	Gene Locus	rsID	Chrom	Pos	Minor Allele	Cases MAF	Controls MAF	OR	OR Lower	OR Upper	P-value	Credible SNP Set	Posterior Probability	Func	CADD	RDB
Total IIM	LINC00924	rs8040452	15	96197257	T	0.375	0.418	0.836	0.785	0.890	1.90E-08	TRUE	0.137	intergenic	2.658	3a
Total IIM	LINC00924	rs11858381	15	96196885	T	0.375	0.418	0.836	0.785	0.891	1.95E-08	TRUE	0.133	intergenic	1.142	3a
Total IIM	LINC00924	rs2397822	15	96196117	G	0.375	0.418	0.837	0.786	0.891	1.96E-08	TRUE	0.132	intergenic	3.506	3a
Total IIM	LINC00924	rs8028739	15	96195106	T	0.375	0.418	0.838	0.786	0.892	2.10E-08	TRUE	0.124	intergenic	3.079	6
Total IIM	LINC00924	rs8026730	15	96193326	G	0.376	0.418	0.840	0.789	0.894	2.95E-08	TRUE	0.089	intergenic	1.98	7
Total IIM	LINC00924	rs1372640	15	96196477	T	0.430	0.469	0.856	0.805	0.911	3.08E-08	TRUE	0.086	intergenic	3.727	3a
Total IIM	LINC00924	rs6496131	15	96198858	G	0.375	0.418	0.837	0.786	0.892	3.10E-08	TRUE	0.085	intergenic	17.02	6
Total IIM	LINC00924	rs1867046	15	96190454	A	0.307	0.341	0.857	0.803	0.916	6.14E-08	TRUE	0.044	intergenic	18.59	7
Total IIM	LINC00924	rs7172984	15	96195993	G	0.312	0.347	0.855	0.800	0.913	6.81E-08	TRUE	0.040	intergenic	2.072	4
Total IIM	LINC00924	rs1442418	15	96190655	T	0.314	0.348	0.858	0.803	0.916	8.38E-08	TRUE	0.032	intergenic	16.89	6
Total IIM	LINC00924	rs1837919	15	96191535	T	0.285	0.316	0.866	0.809	0.926	1.85E-07	TRUE	0.015	intergenic	6.112	6
Total IIM	LINC00924	rs7164937	15	96216000	A	0.383	0.420	0.856	0.804	0.911	3.60E-07	TRUE	0.008	intergenic	6.224	6
Total IIM	LINC00924	rs4984601	15	96207662	C	0.382	0.420	0.853	0.801	0.909	3.65E-07	TRUE	0.008	intergenic	0.213	5
Total IIM	LINC00924	rs7180933	15	96200523	C	0.381	0.420	0.852	0.800	0.907	3.75E-07	TRUE	0.008	intergenic	15.15	7
Total IIM	LINC00924	rs4984600	15	96205095	A	0.317	0.349	0.865	0.810	0.923	4.52E-07	TRUE	0.006	intergenic	0.753	5
Total IIM	LINC00924	rs2397824	15	96202617	T	0.315	0.347	0.864	0.809	0.922	4.66E-07	TRUE	0.006	intergenic	0.123	5

Variants in high LD (r2>0.8) with the most associated variant in the PHTF1-PTPN22 region in IIM

Subgroup	Gene Locus	rsID	Chrom	Pos	Minor Allele	Cases MAF	Controls MAF	OR	OR Lower	OR Upper	P-value	R2
IIM	PHTF1-PTPN22	rs6679677	1	1.14E+08	A	0.124	0.098	1.303	1.185	1.433	1.57E-07	1
IIM	PHTF1-PTPN22	rs2476601	1	1.14E+08	A	0.124	0.098	1.303	1.185	1.432	1.63E-07	0.995

Variants in high LD (r2>0.8) with the most associated variant in the PLCL1 region in IIM

Subgroup	Gene Locus	rsID	Chrom	Pos	Minor Allele	Cases MAF	Controls MAF	OR	OR Lower	OR Upper	P-value	R2
Total IIM	PLCL1	rs1518359	2	198847383	T	0.519	0.486	1.140	1.073	1.212	1.45E-05	1
Total IIM	PLCL1	rs10177758	2	198858917	G	0.521	0.488	1.141	1.073	1.213	1.48E-05	1.000
Total IIM	PLCL1	rs1866664	2	198860428	C	0.521	0.488	1.141	1.073	1.213	1.50E-05	0.999
Total IIM	PLCL1	rs10931797	2	198840793	T	0.519	0.487	1.139	1.072	1.211	1.59E-05	0.999
Total IIM	PLCL1	rs7587043	2	198841729	C	0.517	0.485	1.139	1.071	1.211	1.60E-05	0.999
Total IIM	PLCL1	rs7587598	2	198842244	G	0.517	0.485	1.139	1.071	1.210	1.62E-05	0.999
Total IIM	PLCL1	rs7558450	2	198837766	T	0.519	0.487	1.139	1.071	1.211	1.62E-05	1.000
Total IIM	PLCL1	rs10931796	2	198840661	G	0.519	0.487	1.139	1.071	1.211	1.63E-05	0.999
Total IIM	PLCL1	rs10204166	2	198835264	G	0.520	0.488	1.139	1.071	1.211	1.64E-05	0.997
Total IIM	PLCL1	rs10169453	2	198835212	C	0.520	0.488	1.139	1.071	1.211	1.65E-05	0.997
Total IIM	PLCL1	rs11675025	2	198838315	A	0.519	0.487	1.139	1.071	1.211	1.65E-05	0.999
Total IIM	PLCL1	rs7571869	2	198841615	C	0.519	0.486	1.139	1.071	1.211	1.66E-05	1.000
Total IIM	PLCL1	rs55696134	2	198821569	G	0.520	0.488	1.138	1.070	1.210	1.72E-05	0.997
Total IIM	PLCL1	rs13394871	2	198846337	A	0.519	0.487	1.139	1.071	1.211	1.73E-05	0.999
Total IIM	PLCL1	rs1607376	2	198827914	G	0.520	0.488	1.138	1.070	1.210	1.73E-05	0.997
Total IIM	PLCL1	rs1850633	2	198829582	G	0.520	0.488	1.138	1.071	1.210	1.73E-05	0.997
Total IIM	PLCL1	rs4410288	2	198819824	T	0.520	0.488	1.138	1.070	1.209	1.74E-05	0.997
Total IIM	PLCL1	rs1518360	2	198815303	T	0.519	0.487	1.137	1.070	1.209	1.74E-05	0.997
Total IIM	PLCL1	rs10189897	2	198813410	G	0.519	0.487	1.137	1.069	1.209	1.80E-05	0.997
Total IIM	PLCL1	rs1914690	2	198809368	A	0.519	0.487	1.137	1.069	1.209	1.82E-05	0.997
Total IIM	PLCL1	rs1878732	2	198809545	A	0.519	0.487	1.137	1.069	1.209	1.82E-05	0.997
Total IIM	PLCL1	rs1518366	2	198809196	A	0.519	0.487	1.137	1.069	1.208	1.82E-05	0.997
Total IIM	PLCL1	rs10497809	2	198825668	A	0.518	0.486	1.137	1.069	1.209	1.82E-05	0.996
Total IIM	PLCL1	rs1518364	2	198809975	A	0.519	0.487	1.137	1.069	1.209	1.82E-05	0.997
Total IIM	PLCL1	rs1105078	2	198807885	T	0.519	0.487	1.137	1.069	1.208	1.83E-05	0.996
Total IIM	PLCL1	rs1607373	2	198800532	A	0.519	0.487	1.136	1.069	1.208	1.85E-05	0.996
Total IIM	PLCL1	rs6434946	2	198798497	G	0.518	0.487	1.136	1.069	1.208	1.87E-05	0.996
Total IIM	PLCL1	rs1401090	2	198808578	A	0.519	0.487	1.136	1.069	1.208	1.88E-05	0.996
Total IIM	PLCL1	rs10207433	2	198805930	T	0.519	0.487	1.136	1.069	1.208	1.89E-05	0.996
Total IIM	PLCL1	rs4850817	2	198795204	A	0.516	0.485	1.134	1.067	1.206	1.95E-05	0.994
Total IIM	PLCL1	rs6434945	2	198797960	C	0.519	0.487	1.136	1.068	1.207	1.95E-05	0.996
Total IIM	PLCL1	rs11679040	2	198806829	C	0.517	0.485	1.136	1.068	1.207	2.00E-05	0.995
Total IIM	PLCL1	rs2880389	2	198801707	C	0.517	0.485	1.135	1.068	1.207	2.02E-05	0.995
Total IIM	PLCL1	rs11692344	2	198795863	G	0.519	0.487	1.135	1.068	1.207	2.05E-05	0.995
Total IIM	PLCL1	rs1025549	2	198881183	G	0.460	0.493	0.876	0.824	0.931	2.92E-05	0.934
Total IIM	PLCL1	rs34692727	2	198883513	C	0.460	0.493	0.876	0.824	0.931	2.94E-05	0.934
Total IIM	PLCL1	rs11889326	2	198899275	A	0.459	0.492	0.875	0.823	0.931	2.95E-05	0.934

Total IIM	PLCL1	rs11683222	2	198884028	C	0.460	0.493	0.876	0.824	0.931	2.97E-05	0.934
Total IIM	PLCL1	rs2139049	2	198887660	G	0.459	0.492	0.876	0.824	0.931	2.99E-05	0.934
Total IIM	PLCL1	rs1595823	2	198891799	C	0.459	0.492	0.875	0.823	0.931	3.02E-05	0.934
Total IIM	PLCL1	rs2196171	2	198889807	T	0.459	0.492	0.876	0.823	0.931	3.02E-05	0.934
Total IIM	PLCL1	rs2196172	2	198889893	T	0.459	0.492	0.876	0.823	0.931	3.03E-05	0.934
Total IIM	PLCL1	rs1978888	2	198889245	T	0.459	0.492	0.876	0.823	0.931	3.03E-05	0.934
Total IIM	PLCL1	rs11681663	2	198882952	G	0.460	0.493	0.876	0.824	0.931	3.04E-05	0.934
Total IIM	PLCL1	rs10166845	2	198896071	A	0.459	0.492	0.875	0.823	0.931	3.04E-05	0.934
Total IIM	PLCL1	rs10196612	2	198894139	T	0.459	0.492	0.875	0.823	0.931	3.04E-05	0.934
Total IIM	PLCL1	rs1541953	2	198898344	A	0.461	0.494	0.876	0.824	0.931	3.08E-05	0.934
Total IIM	PLCL1	rs10184227	2	198894305	C	0.459	0.492	0.875	0.823	0.931	3.09E-05	0.934
Total IIM	PLCL1	rs1595824	2	198874006	T	0.525	0.492	1.141	1.073	1.213	3.09E-05	0.989
Total IIM	PLCL1	rs6738825	2	198896895	A	0.458	0.492	0.875	0.823	0.931	3.10E-05	0.934
Total IIM	PLCL1	rs9712275	2	198907143	C	0.444	0.476	0.880	0.827	0.936	3.15E-05	0.916
Total IIM	PLCL1	rs938929	2	198780860	A	0.516	0.485	1.134	1.066	1.205	3.23E-05	0.963
Total IIM	PLCL1	rs4850816	2	198789765	C	0.517	0.485	1.133	1.066	1.205	3.33E-05	0.975
Total IIM	PLCL1	rs12995110	2	198789013	T	0.517	0.485	1.133	1.066	1.205	3.35E-05	0.974
Total IIM	PLCL1	rs1401093	2	198779839	C	0.518	0.487	1.134	1.066	1.205	3.35E-05	0.960
Total IIM	PLCL1	rs13011918	2	198779083	T	0.517	0.486	1.134	1.066	1.205	3.39E-05	0.957
Total IIM	PLCL1	rs1401094	2	198779849	C	0.518	0.487	1.133	1.066	1.205	3.39E-05	0.960
Total IIM	PLCL1	rs10198606	2	198769739	G	0.517	0.486	1.133	1.066	1.205	3.46E-05	0.944
Total IIM	PLCL1	rs1369512	2	198767132	A	0.515	0.484	1.133	1.066	1.204	3.54E-05	0.941
Total IIM	PLCL1	rs6718039	2	198761642	G	0.519	0.488	1.132	1.065	1.204	3.55E-05	0.938
Total IIM	PLCL1	rs6735214	2	198760507	T	0.519	0.488	1.132	1.065	1.203	3.73E-05	0.938
Total IIM	PLCL1	rs9973400	2	198941578	T	0.444	0.474	0.887	0.834	0.943	3.77E-05	0.909
Total IIM	PLCL1	rs1369511	2	198746115	C	0.516	0.485	1.132	1.065	1.204	3.77E-05	0.936
Total IIM	PLCL1	rs10048735	2	198742525	T	0.516	0.485	1.132	1.065	1.204	3.79E-05	0.936
Total IIM	PLCL1	rs9288281	2	198744366	A	0.516	0.485	1.132	1.065	1.204	3.80E-05	0.936
Total IIM	PLCL1	rs10192466	2	198738019	A	0.516	0.485	1.132	1.065	1.204	3.81E-05	0.935
Total IIM	PLCL1	rs6753450	2	198735031	T	0.516	0.485	1.132	1.065	1.204	3.83E-05	0.935
Total IIM	PLCL1	rs1866666	2	198940607	T	0.448	0.478	0.887	0.834	0.943	3.89E-05	0.909
Total IIM	PLCL1	rs1064213	2	198950240	A	0.522	0.494	1.118	1.052	1.189	3.90E-05	0.883
Total IIM	PLCL1	rs2060489	2	198752286	T	0.515	0.484	1.132	1.065	1.203	3.91E-05	0.937
Total IIM	PLCL1	rs770666	2	198611897	A	0.511	0.480	1.133	1.066	1.205	3.92E-05	0.855
Total IIM	PLCL1	rs700647	2	198612852	T	0.511	0.480	1.133	1.066	1.205	3.93E-05	0.855
Total IIM	PLCL1	rs11690149	2	198933804	C	0.448	0.478	0.886	0.833	0.942	3.93E-05	0.910
Total IIM	PLCL1	rs700654	2	198643421	A	0.511	0.480	1.134	1.066	1.205	3.94E-05	0.882
Total IIM	PLCL1	rs35062652	2	198927927	C	0.448	0.479	0.886	0.833	0.942	3.95E-05	0.910
Total IIM	PLCL1	rs7572733	2	198929806	C	0.448	0.478	0.886	0.833	0.942	3.95E-05	0.910

Total IIM	PLCL1	rs1065953	2	198614612	T	0.511	0.480	1.133	1.066	1.205	3.96E-05	0.856
Total IIM	PLCL1	rs11690205	2	198933948	C	0.448	0.478	0.886	0.833	0.942	3.97E-05	0.910
Total IIM	PLCL1	rs1065955	2	198615531	C	0.511	0.480	1.133	1.066	1.205	3.97E-05	0.857
Total IIM	PLCL1	rs771018	2	198650259	A	0.511	0.479	1.133	1.066	1.205	3.98E-05	0.886
Total IIM	PLCL1	rs700649	2	198622752	A	0.511	0.480	1.133	1.066	1.205	3.99E-05	0.862
Total IIM	PLCL1	rs1065954	2	198615017	T	0.511	0.480	1.133	1.066	1.205	3.99E-05	0.857
Total IIM	PLCL1	rs700653	2	198637433	G	0.511	0.480	1.134	1.066	1.205	3.99E-05	0.879
Total IIM	PLCL1	rs6734096	2	198756459	G	0.516	0.485	1.132	1.065	1.203	4.00E-05	0.939
Total IIM	PLCL1	rs1065957	2	198616891	G	0.511	0.480	1.133	1.066	1.205	4.00E-05	0.858
Total IIM	PLCL1	rs1065956	2	198616049	A	0.511	0.480	1.133	1.066	1.205	4.00E-05	0.858
Total IIM	PLCL1	rs700648	2	198622636	T	0.511	0.480	1.133	1.066	1.205	4.00E-05	0.862
Total IIM	PLCL1	rs770667	2	198613308	T	0.511	0.480	1.133	1.066	1.205	4.00E-05	0.855
Total IIM	PLCL1	rs700650	2	198624285	A	0.511	0.480	1.133	1.066	1.205	4.03E-05	0.864
Total IIM	PLCL1	rs771013	2	198625237	A	0.511	0.480	1.134	1.066	1.205	4.03E-05	0.866
Total IIM	PLCL1	rs6742782	2	198755021	C	0.517	0.486	1.132	1.065	1.203	4.04E-05	0.939
Total IIM	PLCL1	rs700641	2	198599233	T	0.511	0.480	1.133	1.065	1.204	4.04E-05	0.851
Total IIM	PLCL1	rs700693	2	198730015	G	0.516	0.485	1.132	1.065	1.203	4.04E-05	0.934
Total IIM	PLCL1	rs1065949	2	198627055	A	0.511	0.480	1.133	1.066	1.205	4.09E-05	0.870
Total IIM	PLCL1	rs700691	2	198727852	G	0.516	0.485	1.132	1.064	1.203	4.10E-05	0.934
Total IIM	PLCL1	rs1318867	2	198880481	C	0.472	0.504	0.878	0.826	0.933	4.10E-05	0.984
Total IIM	PLCL1	rs700689	2	198726649	G	0.516	0.485	1.132	1.064	1.203	4.12E-05	0.934
Total IIM	PLCL1	rs976179	2	198880378	A	0.472	0.504	0.878	0.826	0.933	4.16E-05	0.984
Total IIM	PLCL1	rs700655	2	198643631	A	0.509	0.478	1.133	1.066	1.204	4.18E-05	0.880
Total IIM	PLCL1	rs10497813	2	198914072	G	0.453	0.484	0.883	0.830	0.938	4.35E-05	0.920
Total IIM	PLCL1	rs6734561	2	198725076	T	0.516	0.485	1.131	1.064	1.203	4.40E-05	0.933
Total IIM	PLCL1	rs6732340	2	198912952	C	0.453	0.484	0.882	0.830	0.938	4.44E-05	0.920
Total IIM	PLCL1	rs6434942	2	198756688	A	0.522	0.491	1.130	1.063	1.201	4.46E-05	0.936
Total IIM	PLCL1	rs700679	2	198706916	T	0.516	0.485	1.131	1.064	1.202	4.49E-05	0.929
Total IIM	PLCL1	rs2117339	2	198915734	C	0.453	0.484	0.883	0.830	0.939	4.49E-05	0.920
Total IIM	PLCL1	rs67748055	2	198911426	G	0.452	0.483	0.882	0.829	0.938	4.51E-05	0.921
Total IIM	PLCL1	rs1036333	2	198943787	G	0.472	0.500	0.893	0.840	0.949	4.54E-05	0.886
Total IIM	PLCL1	rs1583792	2	198900288	C	0.450	0.482	0.880	0.828	0.936	4.56E-05	0.921
Total IIM	PLCL1	rs67031482	2	198911166	C	0.452	0.483	0.882	0.829	0.938	4.56E-05	0.921
Total IIM	PLCL1	rs2164068	2	198943852	T	0.472	0.500	0.893	0.840	0.949	4.56E-05	0.886
Total IIM	PLCL1	rs745899	2	198908040	A	0.451	0.483	0.882	0.829	0.937	4.56E-05	0.921
Total IIM	PLCL1	rs700665	2	198673379	C	0.515	0.484	1.131	1.064	1.203	4.60E-05	0.917
Total IIM	PLCL1	rs962210	2	198902230	A	0.450	0.482	0.881	0.828	0.936	4.60E-05	0.921
Total IIM	PLCL1	rs700640	2	198590886	T	0.510	0.479	1.131	1.064	1.203	4.60E-05	0.848
Total IIM	PLCL1	rs2196174	2	198905172	A	0.451	0.482	0.881	0.828	0.937	4.60E-05	0.921

Total IIM	PLCL1	rs6716898	2	198944271	G	0.472	0.500	0.893	0.840	0.950	4.61E-05	0.887
Total IIM	PLCL1	rs2196175	2	198905073	T	0.451	0.482	0.881	0.828	0.937	4.61E-05	0.921
Total IIM	PLCL1	rs1440089	2	198944947	C	0.472	0.500	0.893	0.840	0.950	4.70E-05	0.887
Total IIM	PLCL1	rs696814	2	198687899	G	0.515	0.484	1.131	1.063	1.202	4.81E-05	0.925
Total IIM	PLCL1	rs771010	2	198691006	G	0.515	0.485	1.131	1.063	1.202	4.82E-05	0.926
Total IIM	PLCL1	rs1065952	2	198598585	T	0.514	0.483	1.131	1.064	1.202	4.83E-05	0.850
Total IIM	PLCL1	rs700669	2	198684467	A	0.515	0.484	1.131	1.063	1.202	4.84E-05	0.925
Total IIM	PLCL1	rs12472359	2	198587865	T	0.510	0.479	1.131	1.064	1.202	4.87E-05	0.845
Total IIM	PLCL1	rs11684176	2	198954774	T	0.516	0.489	1.115	1.049	1.185	4.89E-05	0.872
Total IIM	PLCL1	rs12329164	2	198905224	C	0.451	0.483	0.881	0.829	0.937	4.89E-05	0.921
Total IIM	PLCL1	rs1371664	2	198904465	T	0.451	0.483	0.881	0.829	0.937	4.90E-05	0.921
Total IIM	PLCL1	rs10497811	2	198902909	A	0.451	0.483	0.881	0.829	0.937	4.96E-05	0.921
Total IIM	PLCL1	rs4549082	2	198944654	T	0.472	0.500	0.894	0.841	0.950	5.04E-05	0.888
Total IIM	PLCL1	rs1579695	2	198900363	G	0.451	0.483	0.881	0.829	0.937	5.08E-05	0.921
Total IIM	PLCL1	rs770658	2	198669871	C	0.515	0.484	1.130	1.063	1.202	5.10E-05	0.912
Total IIM	PLCL1	rs4417706	2	198944655	A	0.472	0.500	0.894	0.841	0.950	5.15E-05	0.888
Total IIM	PLCL1	rs771015	2	198668294	A	0.515	0.484	1.130	1.063	1.202	5.25E-05	0.910
Total IIM	PLCL1	rs7587251	2	198930197	T	0.446	0.475	0.888	0.835	0.944	5.26E-05	0.906
Total IIM	PLCL1	rs700659	2	198659923	C	0.515	0.484	1.130	1.063	1.202	5.37E-05	0.902
Total IIM	PLCL1	rs771017	2	198660941	A	0.513	0.483	1.130	1.063	1.201	5.42E-05	0.903
Total IIM	PLCL1	rs700657	2	198654324	C	0.513	0.483	1.130	1.062	1.201	5.78E-05	0.896
Total IIM	PLCL1	rs771016	2	198665520	T	0.515	0.484	1.129	1.062	1.201	5.80E-05	0.908
Total IIM	PLCL1	rs4850437	2	198569051	T	0.503	0.473	1.127	1.060	1.198	7.86E-05	0.820
Total IIM	PLCL1	rs10460393	2	198548306	T	0.506	0.476	1.127	1.060	1.198	7.92E-05	0.814
Total IIM	PLCL1	rs12622674	2	198551812	G	0.506	0.476	1.127	1.060	1.198	7.94E-05	0.819
Total IIM	PLCL1	rs6743671	2	198563642	T	0.506	0.476	1.127	1.060	1.198	7.98E-05	0.828
Total IIM	PLCL1	rs2341510	2	198558679	T	0.506	0.476	1.127	1.060	1.198	7.99E-05	0.823
Total IIM	PLCL1	rs7340470	2	198545462	G	0.506	0.476	1.127	1.060	1.198	7.99E-05	0.810
Total IIM	PLCL1	rs13034353	2	198565763	A	0.506	0.476	1.127	1.060	1.198	8.03E-05	0.830
Total IIM	PLCL1	rs57862683	2	198747537	A	0.464	0.493	0.891	0.838	0.947	8.64E-05	0.885
Total IIM	PLCL1	rs4850808	2	198576142	T	0.507	0.477	1.126	1.059	1.197	8.86E-05	0.832
Total IIM	PLCL1	rs10497807	2	198585087	G	0.508	0.478	1.125	1.059	1.196	9.60E-05	0.835
Total IIM	PLCL1	rs4850438	2	198583267	A	0.508	0.478	1.125	1.058	1.196	9.94E-05	0.834
Total IIM	PLCL1	rs13029495	2	198582601	C	0.508	0.479	1.125	1.058	1.196	1.03E-04	0.834

Variants in high LD (r2>0.8) with the most associated variant in the LTBR region in IIM

Subgroup	Gene Locus	rsID	Chrom	Pos	Minor Allel	Cases MAF	Controls MA	OR	OR Lower	OR Upper	P-value	R2
Total IIM	LTBR	rs11064180	12	6523249	T	0.377	0.411	0.867	0.814	0.923	1.58E-05	1
Total IIM	LTBR	rs7307010	12	6525518	G	0.377	0.411	0.867	0.814	0.923	1.68E-05	0.997
Total IIM	LTBR	rs12812284	12	6526617	T	0.377	0.411	0.868	0.815	0.924	1.86E-05	0.996
Total IIM	LTBR	rs2886086	12	6526672	G	0.378	0.412	0.869	0.816	0.925	2.02E-05	0.993
Total IIM	LTBR	rs2886087	12	6526744	G	0.383	0.417	0.868	0.815	0.924	1.82E-05	0.979
Total IIM	LTBR	rs2364487	12	6526934	A	0.377	0.411	0.868	0.815	0.924	1.98E-05	0.996
Total IIM	LTBR	rs11064182	12	6527237	A	0.377	0.411	0.868	0.815	0.925	2.04E-05	0.996
Total IIM	LTBR	rs7961280	12	6528069	A	0.377	0.411	0.868	0.815	0.924	1.92E-05	0.996
Total IIM	LTBR	rs10849453	12	6528252	C	0.365	0.396	0.876	0.822	0.933	7.95E-05	0.939
Total IIM	LTBR	rs11064185	12	6531223	C	0.350	0.381	0.875	0.821	0.933	7.29E-05	0.871
Total IIM	LTBR	rs7295359	12	6531606	G	0.349	0.380	0.874	0.820	0.932	6.85E-05	0.875
Total IIM	LTBR	rs8181686	12	6531951	C	0.350	0.381	0.876	0.821	0.934	8.39E-05	0.871
Total IIM	LTBR	rs11064186	12	6532015	G	0.349	0.380	0.875	0.821	0.933	7.97E-05	0.875
Total IIM	LTBR	rs4764589	12	6532282	A	0.349	0.380	0.875	0.821	0.933	8.07E-05	0.875
Total IIM	LTBR	rs4764590	12	6532350	G	0.349	0.380	0.875	0.821	0.933	8.13E-05	0.875
Total IIM	LTBR	rs2364491	12	6535887	T	0.337	0.365	0.883	0.828	0.942	0.000243	0.826
Total IIM	LTBR	rs2886089	12	6535981	C	0.348	0.379	0.874	0.820	0.932	6.15E-05	0.873
Total IIM	LTBR	rs7976678	12	6537544	A	0.347	0.376	0.880	0.825	0.938	0.000126	0.866

Variants in high LD (r2>0.8) with the most associated variant in the DGKQ region in IIM

Subgroup	Gene Locus	rsID	Chrom	Pos	Minor Allele	Cases MAF	Controls MAF	OR	OR Lower	OR Upper	P-value	R2
Total IIM	DGKQ	rs6599390	4	956047	A	0.302	0.340	0.838	0.784	0.895	1.63724E-07	1
Total IIM	DGKQ	rs28671147	4	955084	A	0.302	0.341	0.838	0.784	0.895	1.7323E-07	0.999
Total IIM	DGKQ	rs3822020	4	985727	A	0.320	0.358	0.844	0.790	0.901	2.9159E-07	0.877
Total IIM	DGKQ	rs3796622	4	983060	T	0.321	0.358	0.844	0.791	0.901	3.69684E-07	0.886
Total IIM	DGKQ	rs4690163	4	975137	T	0.321	0.358	0.845	0.792	0.902	4.78474E-07	0.896
Total IIM	DGKQ	rs11936407	4	987276	T	0.322	0.359	0.847	0.793	0.904	5.01318E-07	0.865
Total IIM	DGKQ	rs6814642	4	978388	A	0.320	0.358	0.846	0.793	0.903	5.42962E-07	0.894
Total IIM	DGKQ	rs4690219	4	977270	G	0.321	0.359	0.846	0.793	0.903	5.91222E-07	0.893
Total IIM	DGKQ	rs3806756	4	987391	C	0.307	0.342	0.854	0.799	0.912	1.12597E-06	0.850

Variants in high LD (r2>0.8) with the most associated variant in the TEC region in IIM

Subgroup	Gene Locus	rsID	Chrom	Pos	Minor Allele	Cases MAF	Controls MAF	OR	OR Lower	OR Upper	P-value	R2
Total IIM	TEC	rs80105690	4	48155618	T	0.085	0.068	1.271	1.136	1.422	6E-06	1
Total IIM	TEC	rs138972385	4	48144333	G	0.086	0.069	1.258	1.125	1.407	1.21E-05	0.983

Variants in high LD (r2>0.8) with the most associated variant in the NAB1 region in PM

Subgroup	Gene Locus	rsID	Chrom	Pos	Minor Allel	Cases MAF	ontrols MA	OR	OR Lower	OR Upper	P-value	Credible SNP Set	terior Probabil	Func	CADD	RDB
PM	NAB1	rs6733720	2	191516020	G	0.220	0.167	1.405	1.237	1.596	1.96E-08	TRUE	0.285	intronic	2.09	5
PM	NAB1	rs74925618	2	191377783	C	0.094	0.066	1.482	1.234	1.780	7.79E-08	TRUE	0.075	intronic	5.997	7
PM	NAB1	rs1558471	2	191506773	C	0.180	0.131	1.450	1.263	1.664	1.32E-07	TRUE	0.045	intergenic	8.88	5
PM	NAB1	rs4853724	2	191510532	C	0.179	0.131	1.453	1.266	1.669	1.35E-07	TRUE	0.044	upstream	0.699	7
PM	NAB1	rs12467273	2	191519135	A	0.198	0.149	1.415	1.239	1.615	1.56E-07	TRUE	0.038	intronic	1.6	5
PM	NAB1	rs55999263	2	191531868	T	0.179	0.129	1.464	1.275	1.682	1.77E-07	TRUE	0.034	intronic	5.822	4
PM	NAB1	rs4853726	2	191533300	C	0.179	0.129	1.465	1.275	1.683	1.81E-07	TRUE	0.033	intronic	9.282	5
PM	NAB1	rs4853725	2	191533171	A	0.179	0.129	1.465	1.275	1.682	1.82E-07	TRUE	0.033	intronic	7.465	4
PM	NAB1	rs16832798	2	191534372	C	0.179	0.129	1.466	1.276	1.684	1.82E-07	TRUE	0.033	intronic	2.505	7
PM	NAB1	rs2286896	2	191535576	C	0.179	0.129	1.467	1.277	1.685	1.83E-07	TRUE	0.033	intronic	6.48	NA
PM	NAB1	rs2286895	2	191535698	A	0.179	0.129	1.466	1.277	1.684	1.85E-07	TRUE	0.032	intronic	0.242	NA
PM	NAB1	rs33999198	2	191518355	A	0.178	0.130	1.455	1.267	1.671	1.93E-07	TRUE	0.031	intronic	2.343	7
PM	NAB1	rs716254	2	191528101	A	0.179	0.130	1.460	1.271	1.678	1.96E-07	TRUE	0.031	intronic	5.807	5
PM	NAB1	rs4853514	2	191517188	C	0.178	0.130	1.453	1.265	1.669	1.98E-07	TRUE	0.030	intronic	2.868	7
PM	NAB1	rs1860849	2	191518860	C	0.178	0.130	1.455	1.267	1.672	1.99E-07	TRUE	0.030	intronic	0.795	1d
PM	NAB1	rs12612666	2	191516966	A	0.178	0.130	1.452	1.264	1.668	1.99E-07	TRUE	0.030	intronic	5.565	5
PM	NAB1	rs10931468	2	191538562	A	0.174	0.127	1.453	1.263	1.671	4.68E-07	TRUE	0.013	intronic	7.018	4
PM	NAB1	rs78415886	2	191434281	G	0.084	0.058	1.497	1.234	1.815	5.09E-07	TRUE	0.012	intergenic	0.288	5
PM	NAB1	rs12612719	2	191538239	C	0.175	0.127	1.450	1.261	1.668	5.12E-07	TRUE	0.012	intronic	7.49	7
PM	NAB1	rs12622264	2	191538296	A	0.175	0.127	1.450	1.261	1.668	5.17E-07	TRUE	0.012	intronic	6.237	5
PM	NAB1	rs60518431	2	191541839	C	0.174	0.127	1.448	1.259	1.665	5.40E-07	TRUE	0.012	cRNA_exon	0.456	6
PM	NAB1	rs3771317	2	191543962	C	0.174	0.127	1.446	1.257	1.663	5.60E-07	TRUE	0.011	RNA_intror	7.508	NA
PM	NAB1	rs111360682	2	191476950	T	0.085	0.058	1.509	1.245	1.828	5.87E-07	TRUE	0.011	intergenic	0.856	5
PM	NAB1	rs4853515	2	191552575	C	0.174	0.128	1.442	1.254	1.658	6.12E-07	TRUE	0.010	RNA_intror	1.981	5
PM	NAB1	rs16832834	2	191554447	A	0.174	0.128	1.440	1.252	1.656	6.50E-07	TRUE	0.010	RNA_intror	1.926	7
PM	NAB1	rs16832836	2	191558030	G	0.174	0.128	1.438	1.251	1.654	6.58E-07	TRUE	0.010	RNA_intror	0.778	7

Variants in high LD (r2>0.8) with the most associated variant in the PTPN22 region in PM

Subgroup	Gene Locus	rsID	Chrom	Pos	Minor Allele	Cases MAF	Controls MAF	OR	OR Lower	OR Upper	P-value	R2
PM	PTPN22	rs2476601	1	114377568	A	0.146	0.105	1.465	1.260	1.703	1.25E-06	1
PM	PTPN22	rs6679677	1	114303808	A	0.146	0.105	1.461	1.256	1.699	1.52E-06	0.995

Variants in high LD (r2>0.8) with the most associated variant in the CCR5 region in IBM

Subgroup	Gene Locus	rsID	Chrom	Pos	Minor Allele	Cases MAF	Controls MAF	OR	OR Lower	OR Upper	P-value	R2
IBM	CCR5	rs41490645	3	46410137	C	0.079	0.161	0.448	0.318	0.633	5.96E-07	1
IBM	CCR5	rs112088397	3	46389462	T	0.079	0.161	0.450	0.319	0.635	7.02E-07	0.995
IBM	CCR5	rs3092960	3	46400062	A	0.079	0.161	0.450	0.319	0.635	7.02E-07	0.995
IBM	CCR5	rs113647051	3	46366857	C	0.079	0.160	0.452	0.320	0.637	8.01E-07	0.986
IBM	CCR5	rs113507038	3	46357822	T	0.079	0.160	0.452	0.320	0.638	8.04E-07	0.986
IBM	CCR5	rs112384491	3	46360359	G	0.079	0.160	0.452	0.320	0.638	8.04E-07	0.986
IBM	CCR5	rs111958022	3	46459956	G	0.083	0.165	0.459	0.328	0.644	8.45E-07	0.923
IBM	CCR5	rs80054040	3	46340285	A	0.079	0.160	0.454	0.321	0.640	9.30E-07	0.983
IBM	CCR5	rs79629761	3	46340630	T	0.079	0.160	0.454	0.321	0.640	9.30E-07	0.983
IBM	CCR5	rs75352297	3	46345151	C	0.079	0.160	0.454	0.321	0.640	9.30E-07	0.983
IBM	CCR5	rs78372062	3	46323679	A	0.079	0.159	0.455	0.323	0.642	9.31E-07	0.962
IBM	CCR5	rs112870257	3	46459398	A	0.083	0.164	0.461	0.329	0.646	9.66E-07	0.925
IBM	CCR5	rs113888916	3	46459411	T	0.083	0.164	0.461	0.329	0.646	9.66E-07	0.925
IBM	CCR5	rs6808835	3	46449864	T	0.083	0.165	0.461	0.329	0.646	9.75E-07	0.925
IBM	CCR5	rs1140865	3	46450884	T	0.083	0.165	0.461	0.329	0.646	9.75E-07	0.925
IBM	CCR5	rs2157061	3	46451502	A	0.083	0.165	0.461	0.329	0.646	9.75E-07	0.925
IBM	CCR5	rs2157062	3	46451647	T	0.083	0.165	0.461	0.329	0.646	9.75E-07	0.925
IBM	CCR5	rs6762266	3	46452863	C	0.083	0.165	0.461	0.329	0.646	9.75E-07	0.925
IBM	CCR5	rs113774564	3	46454682	A	0.083	0.165	0.461	0.329	0.646	9.75E-07	0.926
IBM	CCR5	rs113069082	3	46454688	A	0.083	0.165	0.461	0.329	0.646	9.75E-07	0.926
IBM	CCR5	rs111514151	3	46455754	C	0.083	0.165	0.461	0.329	0.646	9.75E-07	0.926
IBM	CCR5	rs113942981	3	46457329	T	0.083	0.165	0.461	0.329	0.646	9.75E-07	0.925
IBM	CCR5	rs6791599	3	46458942	A	0.083	0.165	0.461	0.329	0.646	9.75E-07	0.925
IBM	CCR5	rs111959715	3	46459050	C	0.083	0.165	0.461	0.329	0.646	9.75E-07	0.925
IBM	CCR5	rs113710186	3	46459914	C	0.083	0.165	0.461	0.329	0.646	9.75E-07	0.925
IBM	CCR5	rs113064717	3	46460013	C	0.083	0.165	0.461	0.329	0.646	9.75E-07	0.925
IBM	CCR5	rs6791789	3	46459121	A	0.083	0.165	0.461	0.329	0.646	1.04E-06	0.931
IBM	CCR5	rs111890592	3	46317354	T	0.080	0.159	0.458	0.325	0.645	1.06E-06	0.962
IBM	CCR5	rs78729754	3	46339185	A	0.079	0.159	0.455	0.323	0.642	1.07E-06	0.978
IBM	CCR5	rs6782522	3	46458505	A	0.083	0.164	0.463	0.330	0.648	1.11E-06	0.931
IBM	CCR5	rs6798291	3	46443931	C	0.083	0.164	0.463	0.330	0.648	1.12E-06	0.932
IBM	CCR5	rs112903945	3	46445343	C	0.083	0.164	0.463	0.330	0.648	1.12E-06	0.932
IBM	CCR5	rs11574428	3	46446721	A	0.083	0.164	0.463	0.330	0.648	1.12E-06	0.932
IBM	CCR5	rs11574429	3	46446810	C	0.083	0.164	0.463	0.330	0.648	1.12E-06	0.932
IBM	CCR5	rs11574434	3	46447915	A	0.083	0.164	0.463	0.330	0.648	1.12E-06	0.932

IBM	CCR5	rs6441977	3	46450072	A	0.083	0.164	0.463	0.330	0.648	1.12E-06	0.931
IBM	CCR5	rs6770674	3	46452873	A	0.083	0.164	0.463	0.330	0.648	1.12E-06	0.931
IBM	CCR5	rs112384806	3	46453487	T	0.083	0.164	0.463	0.330	0.648	1.12E-06	0.931
IBM	CCR5	rs113490452	3	46458228	A	0.083	0.164	0.463	0.330	0.648	1.12E-06	0.931
IBM	CCR5	rs113365357	3	46459291	A	0.083	0.164	0.463	0.330	0.648	1.12E-06	0.931
IBM	CCR5	rs111410234	3	46460675	T	0.083	0.164	0.463	0.330	0.648	1.12E-06	0.931
IBM	CCR5	rs3136535	3	46409113	A	0.081	0.161	0.461	0.328	0.648	1.22E-06	0.992
IBM	CCR5	rs112712359	3	46461783	A	0.083	0.164	0.464	0.332	0.651	1.28E-06	0.933
IBM	CCR5	rs762790	3	46402688	G	0.081	0.161	0.462	0.329	0.650	1.39E-06	0.990
IBM	CCR5	rs57093591	3	46350716	C	0.081	0.161	0.462	0.329	0.650	1.48E-06	0.977
IBM	CCR5	rs2856758	3	46411661	G	0.085	0.166	0.470	0.336	0.656	1.52E-06	0.946
IBM	CCR5	rs74433128	3	46279933	G	0.081	0.160	0.466	0.331	0.655	1.61E-06	0.958
IBM	CCR5	rs76733709	3	46281461	A	0.081	0.160	0.466	0.331	0.655	1.61E-06	0.958
IBM	CCR5	rs113676185	3	46286046	T	0.081	0.160	0.466	0.331	0.655	1.61E-06	0.958
IBM	CCR5	rs113255842	3	46435161	A	0.087	0.168	0.475	0.341	0.661	1.63E-06	0.906
IBM	CCR5	rs113485180	3	46293070	C	0.081	0.160	0.467	0.332	0.656	1.68E-06	0.960
IBM	CCR5	rs112982903	3	46433247	G	0.087	0.168	0.475	0.342	0.662	1.69E-06	0.905
IBM	CCR5	rs111961837	3	46433146	T	0.087	0.168	0.475	0.342	0.662	1.69E-06	0.905
IBM	CCR5	rs113229741	3	46433987	C	0.087	0.167	0.476	0.342	0.662	1.71E-06	0.905
IBM	CCR5	rs112368251	3	46433967	A	0.087	0.167	0.476	0.342	0.662	1.71E-06	0.905
IBM	CCR5	rs916094	3	46444835	C	0.085	0.165	0.471	0.338	0.658	1.76E-06	0.917
IBM	CCR5	rs75994669	3	46300863	G	0.081	0.159	0.468	0.333	0.658	1.85E-06	0.961
IBM	CCR5	rs79539493	3	46302938	A	0.081	0.159	0.468	0.333	0.658	1.85E-06	0.961
IBM	CCR5	rs11575821	3	46422355	A	0.087	0.167	0.477	0.342	0.663	1.86E-06	0.909
IBM	CCR5	rs916093	3	46444794	C	0.085	0.165	0.473	0.339	0.661	2.01E-06	0.921
IBM	CCR5	rs34445878	3	46425711	T	0.087	0.167	0.478	0.344	0.666	2.12E-06	0.909
IBM	CCR5	rs111669359	3	46427616	C	0.087	0.167	0.478	0.344	0.666	2.12E-06	0.908
IBM	CCR5	rs7652037	3	46423063	C	0.087	0.167	0.478	0.344	0.666	2.12E-06	0.909
IBM	CCR5	rs34291293	3	46425647	A	0.087	0.167	0.478	0.344	0.666	2.13E-06	0.909
IBM	CCR5	rs4452362	3	46470654	T	0.087	0.166	0.482	0.346	0.670	2.19E-06	0.854
IBM	CCR5	rs112479910	3	46471119	C	0.087	0.166	0.482	0.346	0.670	2.20E-06	0.854
IBM	CCR5	rs113595266	3	46471525	A	0.087	0.166	0.482	0.346	0.670	2.20E-06	0.854
IBM	CCR5	rs111713092	3	46431751	T	0.087	0.166	0.479	0.344	0.667	2.26E-06	0.908
IBM	CCR5	rs6787972	3	46427835	A	0.085	0.164	0.477	0.341	0.666	2.48E-06	0.931
IBM	CCR5	rs113263161	3	46425718	A	0.085	0.163	0.478	0.343	0.668	2.86E-06	0.933
IBM	CCR5	rs73833033	3	46277002	C	0.083	0.160	0.476	0.340	0.668	2.90E-06	0.946
IBM	CCR5	rs60020651	3	46287651	C	0.083	0.160	0.476	0.340	0.668	2.90E-06	0.949

IBM	CCR5	rs7645306	3	46474670	A	0.089	0.167	0.488	0.352	0.678	2.93E-06	0.842
IBM	CCR5	rs77648833	3	46474955	G	0.089	0.167	0.488	0.352	0.678	2.93E-06	0.842
IBM	CCR5	rs75820382	3	46475122	T	0.089	0.167	0.488	0.352	0.678	2.94E-06	0.843
IBM	CCR5	rs56063910	3	46477826	T	0.089	0.167	0.488	0.352	0.678	2.94E-06	0.843
IBM	CCR5	rs75094754	3	46477830	T	0.089	0.167	0.488	0.352	0.678	2.94E-06	0.843
IBM	CCR5	rs113934302	3	46432856	A	0.089	0.168	0.486	0.350	0.674	3.00E-06	0.894
IBM	CCR5	rs75630441	3	46429717	A	0.089	0.167	0.488	0.352	0.677	3.58E-06	0.899
IBM	CCR5	rs35388950	3	46429679	T	0.089	0.167	0.488	0.352	0.677	3.59E-06	0.898
IBM	CCR5	rs112359020	3	46429391	G	0.089	0.167	0.488	0.352	0.677	3.62E-06	0.899
IBM	CCR5	rs111960587	3	46482049	G	0.089	0.166	0.492	0.354	0.683	3.71E-06	0.849
IBM	CCR5	rs34971514	3	46427354	T	0.089	0.167	0.488	0.352	0.678	3.72E-06	0.899
IBM	CCR5	rs113634435	3	46431695	G	0.089	0.167	0.489	0.353	0.679	3.97E-06	0.898
IBM	CCR5	rs4453882	3	46472651	C	0.091	0.168	0.497	0.359	0.688	4.47E-06	0.841
IBM	CCR5	rs73833032	3	46276490	C	0.085	0.160	0.489	0.350	0.683	5.52E-06	0.946
IBM	CCR5	rs79893749	3	46253650	T	0.081	0.154	0.486	0.345	0.683	7.44E-06	0.868
IBM	CCR5	rs112160248	3	46260225	C	0.082	0.154	0.491	0.349	0.690	8.97E-06	0.868
IBM	CCR5	rs58697594	3	46275570	A	0.089	0.162	0.508	0.366	0.706	1.29E-05	0.909

GARFIELD analysis showing enrichment of IIM associations within regulatory and functional annotations of blood cells for assocation p-value thresholds 1x10-5 and 1x10-8

PThresh	OR	Pvalue	Beta	SE	CI95_lower	CI95_upper	NAnnotThesh	NAnnot	NThresh	N	linkID	Annotation	Celltype	Tissue	Type	Category
1.00E-05	5.370828	1.10E-16	1.680982	0.202687721	1.283714122	2.07824999	64	6640	132	46896	296	UW.CD19_Primary_Cells.ChromatinAc	CD19_Primary_Cells	blood	hotspots	Hotspots
1.00E-08	7.133216	2.96E-15	1.964762	0.248931752	1.476855935	2.452668404	44	5874	85	46896	300	UW.CD3_Primary_Cells.ChromatinAcc	CD3_Primary_Cells	blood	hotspots	Hotspots
1.00E-08	6.325792	9.23E-15	1.844635	0.238034542	1.37808752	2.311182926	42	5345	85	46896	939	H3K79me2_gm12878	GM12878	blood	H3K79me2	Histone_Modifications
1.00E-08	6.350246	9.27E-14	1.848494	0.248089702	1.362237699	2.334749329	39	4719	85	46896	943	H3K9ac_gm12878	GM12878	blood	H3K9ac	Histone_Modifications
1.00E-05	4.340074	9.55E-14	1.467891	0.197112876	1.081550205	1.854232677	57	6083	132	46896	905	H3K27ac_gm12878	GM12878	blood	H3K27ac	Histone_Modifications
1.00E-08	5.936315	1.05E-13	1.781089	0.239579938	1.311511896	2.250665252	43	6083	85	46896	905	H3K27ac_gm12878	GM12878	blood	H3K27ac	Histone_Modifications
1.00E-05	4.439094	1.63E-13	1.49045	0.20207296	1.094387222	1.886513225	62	7088	132	46896	295	UW.CD19_Primary_Cells.ChromatinAc	CD19_Primary_Cells	blood	hotspots	Hotspots
1.00E-08	6.126603	2.66E-13	1.81264	0.247951009	1.326656393	2.298624349	44	6640	85	46896	296	UW.CD19_Primary_Cells.ChromatinAc	CD19_Primary_Cells	blood	hotspots	Hotspots
1.00E-05	4.501974	2.67E-13	1.504516	0.205817381	1.101113998	1.90791813	56	5874	132	46896	300	UW.CD3_Primary_Cells.ChromatinAcc	CD3_Primary_Cells	blood	hotspots	Hotspots
1.00E-05	4.368719	4.26E-13	1.47447	0.203456455	1.075695106	1.87324441	63	7279	132	46896	297	UW.CD19_Primary_Cells.ChromatinAc	CD19_Primary_Cells	blood	hotspots	Hotspots
1.00E-08	5.924301	5.80E-13	1.779063	0.246918606	1.295102183	2.263023119	43	5956	85	46896	933	H3K4me3_gm12878	GM12878	blood	H3K4me3	Histone_Modifications
1.00E-08	5.811684	1.00E-12	1.75987	0.246827392	1.276088678	2.243652053	45	7088	85	46896	295	UW.CD19_Primary_Cells.ChromatinAc	CD19_Primary_Cells	blood	hotspots	Hotspots
1.00E-05	4.239963	3.05E-12	1.444555	0.207097464	1.038643523	1.85046558	48	4719	132	46896	943	H3K9ac_gm12878	GM12878	blood	H3K9ac	Histone_Modifications
1.00E-05	3.968451	1.18E-11	1.378376	0.203221038	0.980062523	1.77668899	54	5956	132	46896	933	H3K4me3_gm12878	GM12878	blood	H3K4me3	Histone_Modifications
1.00E-05	3.859348	1.62E-11	1.350498	0.200457537	0.957601391	1.743394937	64	7729	132	46896	53	CD20-DS17541.hg19.twopass.merge1	CD20+	blood	hotspots	Hotspots
1.00E-08	5.061182	2.27E-11	1.6216	0.242486664	1.14632626	2.096873984	46	7827	85	46896	927	H3K4me2_gm12878	GM12878	blood	H3K4me2	Histone_Modifications
1.00E-08	5.093587	5.91E-11	1.627982	0.248695343	1.140539469	2.115425213	43	7279	85	46896	297	UW.CD19_Primary_Cells.ChromatinAc	CD19_Primary_Cells	blood	hotspots	Hotspots
1.00E-08	4.735028	2.25E-10	1.554988	0.245139048	1.074515072	2.035460141	44	7729	85	46896	53	CD20-DS17541.hg19.twopass.merge1	CD20+	blood	hotspots	Hotspots
1.00E-05	3.822396	2.27E-10	1.340877	0.21143531	0.926464152	1.755290569	49	5191	132	46896	307	UW.CD56_Primary_Cells.ChromatinAc	CD56_Primary_Cells	blood	hotspots	Hotspots
1.00E-05	3.465155	3.57E-10	1.242757	0.198148199	0.854386923	1.631127863	61	7827	132	46896	927	H3K4me2_gm12878	GM12878	blood	H3K4me2	Histone_Modifications
1.00E-05	3.656467	3.85E-10	1.296497	0.207109884	0.890561875	1.702432622	55	6312	132	46896	304	UW.CD4_Primary_Cells.ChromatinAcc	CD4_Primary_Cells	blood	hotspots	Hotspots
1.00E-08	4.89175	4.91E-10	1.58755	0.255152349	1.087451521	2.087648728	35	5191	85	46896	307	UW.CD56_Primary_Cells.ChromatinAc	CD56_Primary_Cells	blood	hotspots	Hotspots
1.00E-05	3.504496	1.93E-09	1.254047	0.208875434	0.844650756	1.663442457	51	5860	132	46896	301	UW.CD3_Primary_Cells.ChromatinAcc	CD3_Primary_Cells	blood	hotspots	Hotspots
1.00E-05	3.578715	2.44E-09	1.275004	0.213733735	0.856085594	1.693921833	50	5550	132	46896	299	UW.CD3_Primary_Cells.ChromatinAcc	CD3_Primary_Cells	blood	hotspots	Hotspots
1.00E-05	3.086159	3.75E-09	1.126927	0.19117564	0.752222893	1.501631403	60	8693	132	46896	923	H3K4me1_gm12878	GM12878	blood	H3K4me1	Histone_Modifications
1.00E-05	4.267183	5.32E-09	1.450954	0.248590996	0.963715648	1.938192353	31	2633	132	46896	182	GM12864-DS12431.hotspot.twopass.f	GM12864	blood	peaks	Peaks
1.00E-05	3.360852	6.91E-09	1.212194	0.209246968	0.802070334	1.622318449	50	6193	132	46896	57	GM06990-DS7748.twopass.merge150	GM06990	blood	hotspots	Hotspots
1.00E-08	5.613753	6.97E-09	1.72522	0.297878346	1.141377969	2.309061084	23	2633	85	46896	182	GM12864-DS12431.hotspot.twopass.f	GM12864	blood	peaks	Peaks
1.00E-05	3.209728	8.39E-09	1.166186	0.202447245	0.769389723	1.562982925	45	5345	132	46896	939	H3K79me2_gm12878	GM12878	blood	H3K79me2	Histone_Modifications
1.00E-05	3.207816	1.11E-08	1.16559	0.204000773	0.765748712	1.565431742	60	8177	132	46896	309	UW.CD8_Primary_Cells.ChromatinAcc	CD8_Primary_Cells	blood	hotspots	Hotspots
1.00E-08	5.51752	1.14E-08	1.707928	0.299160062	1.121574694	2.294282139	20	2075	85	46896	609	UW.CD8_Primary_Cells.ChromatinAcc	CD8_Primary_Cells	blood	peaks	Peaks
1.00E-05	3.159913	1.31E-08	1.150545	0.202392854	0.753854643	1.547234632	59	7731	132	46896	302	UW.CD3_Primary_Cells.ChromatinAcc	CD3_Primary_Cells	blood	hotspots	Hotspots
1.00E-08	3.765349	1.37E-08	1.32584	0.233518254	0.868144694	1.78353625	43	8693	85	46896	923	H3K4me1_gm12878	GM12878	blood	H3K4me1	Histone_Modifications
1.00E-08	4.810012	1.39E-08	1.570699	0.27680565	1.028160404	2.113238551	25	2898	85	46896	987	seg_TSS_gm12878	GM12878	blood	TSS	Chromatin_States
1.00E-08	4.18517	1.59E-08	1.431547	0.253319409	0.935041249	1.92805333	36	6193	85	46896	57	GM06990-DS7748.twopass.merge150	GM06990	blood	hotspots	Hotspots
1.00E-05	3.109856	1.79E-08	1.134577	0.201496007	0.73964436	1.529508708	59	7736	132	46896	576	UW.Mobilized_CD4_Primary_Cells.Ch	Mobilized_CD4_Primary_(	blood	hotspots	Hotspots
1.00E-05	4.221006	1.85E-08	1.440073	0.255972612	0.938367113	1.941779752	26	1979	132	46896	596	UW.CD19_Primary_Cells.ChromatinAc	CD19_Primary_Cells	blood	peaks	Peaks
1.00E-08	5.414165	1.86E-08	1.689019	0.300289907	1.100450497	2.277586931	21	2337	85	46896	603	UW.CD4_Primary_Cells.ChromatinAcc	CD4_Primary_Cells	blood	peaks	Peaks
1.00E-05	0.354345	1.96E-08	-1.037485	0.184760802	-1.399616332	-0.675353986	57	33935	132	46896	975	seg_REPRESSED_gm12878	GM12878	blood	REPRESSED	Chromatin_States
1.00E-05	3.254181	2.00E-08	1.179941	0.210246616	0.767857308	1.592024042	56	7693	132	46896	303	UW.CD4_Primary_Cells.ChromatinAcc	CD4_Primary_Cells	blood	hotspots	Hotspots
1.00E-05	3.251344	2.23E-08	1.179068	0.210816573	0.765867867	1.592268833	54	7385	132	46896	58	GM12864-DS12431.twopass.merge15	GM12864	blood	hotspots	Hotspots
1.00E-05	3.690784	2.32E-08	1.305839	0.233747932	0.847692975	1.763984869	32	2898	132	46896	987	seg_TSS_gm12878	GM12878	blood	TSS	Chromatin_States
1.00E-08	4.134201	2.81E-08	1.419294	0.255597844	0.918322344	1.920265892	39	7385	85	46896	58	GM12864-DS12431.twopass.merge15	GM12864	blood	hotspots	Hotspots
1.00E-08	4.49473	3.69E-08	1.502906	0.272995553	0.967834256	2.037976824	30	4988	85	46896	999	tfbs_gm12878	GM12878	blood	tfbs	TFBS
1.00E-08	3.915728	3.97E-08	1.365001	0.248542585	0.877857727	1.852144661	41	8177	85	46896	309	UW.CD8_Primary_Cells.ChromatinAcc	CD8_Primary_Cells	blood	hotspots	Hotspots
1.00E-08	5.266368	4.71E-08	1.661341	0.30417135	1.065165128	2.25751682	20	1979	85	46896	596	UW.CD19_Primary_Cells.ChromatinAc	CD19_Primary_Cells	blood	peaks	Peaks
1.00E-08	5.19203	6.67E-08	1.647125	0.305025551	1.049274732	2.244974892	19	1990	85	46896	605	UW.CD4_Primary_Cells.ChromatinAcc	CD4_Primary_Cells	blood	peaks	Peaks
1.00E-08	3.754107	8.94E-08	1.32285	0.24739556	0.837955037	1.807745631	46	10500	85	46896	60	GM12878-all.twopass.merge150.wgt1	GM12878	blood	hotspots	Hotspots
1.00E-05	3.796696	1.49E-07	1.334131	0.253963548	0.836362777	1.831899884	25	2245	132	46896	593	UW.CD14_Primary_Cells.ChromatinAc	CD14_Primary_Cells	blood	peaks	Peaks
1.00E-08	4.845552	1.84E-07	1.578061	0.302609545	0.984946503	2.171175921	19	2245	85	46896	593	UW.CD14_Primary_Cells.ChromatinAc	CD14_Primary_Cells	blood	peaks	Peaks
1.00E-05	3.853221	1.97E-07	1.348909	0.259289747	0.840701466	1.857117275	24	1990	132	46896	605	UW.CD4_Primary_Cells.ChromatinAcc	CD4_Primary_Cells	blood	peaks	Peaks
1.00E-05	3.771242	2.01E-07	1.327404	0.255340049	0.826937826	1.827870817	27	2337	132	46896	603	UW.CD4_Primary_Cells.ChromatinAcc	CD4_Primary_Cells	blood	peaks	Peaks
1.00E-08	4.716326	2.06E-07	1.55103	0.298642135	0.965691417	2.136368588	21	2737	85	46896	866	UW.Mobilized_CD34_Primary_Cells.C	Mobilized_CD34_Primary_	blood	peaks	Peaks
1.00E-05	2.985989	2.11E-07	1.093931	0.210803222	0.680756817	1.507105446	52	7204	132	46896	308	UW.CD56_Primary_Cells.ChromatinAc	CD56_Primary_Cells	blood	hotspots	Hotspots
1.00E-08	4.76835	2.69E-07	1.562	0.303650829	0.966844791	2.157156042	20	2508	85	46896	871	UW.Mobilized_CD34_Primary_Cells.C	Mobilized_CD34_Primary_	blood	peaks	Peaks
1.00E-08	4.741046	3.08E-07	1.556258	0.304059254	0.960301609	2.152213883	20	2505	85	46896	861	UW.Mobilized_CD34_Primary_Cells.C	Mobilized_CD34_Primary_	blood	peaks	Peaks

1.00E-05	3.341788	4.47E-07	1.206506	0.239008691	0.738048859	1.674962928	29	2866	132	46896	963	seg_ENHANCER_gm12878	GM12878	blood	ENHANCER	Chromatin_States
1.00E-05	3.699973	4.99E-07	1.308326	0.260270387	0.798195549	1.818455464	25	2099	132	46896	594	UW.CD19_Primary_Cells.ChromatinAc	CD19_Primary_Cells	blood	peaks	Peaks
1.00E-08	3.614291	5.32E-07	1.284896	0.256232765	0.78267957	1.787112007	36	7204	85	46896	308	UW.CD56_Primary_Cells.ChromatinAc	CD56_Primary_Cells	blood	hotspots	Hotspots
1.00E-08	4.574337	5.97E-07	1.520462	0.304566189	0.923511987	2.117411449	20	2588	85	46896	865	UW.Mobilized_CD34_Primary_Cells.C	Mobilized_CD34_Primary_	blood	peaks	Peaks
1.00E-08	3.594646	6.18E-07	1.279446	0.256631548	0.776447756	1.782443424	33	5860	85	46896	301	UW.CD3_Primary_Cells.ChromatinAcc	CD3_Primary_Cells	blood	hotspots	Hotspots
1.00E-08	4.614416	6.70E-07	1.529185	0.3076904	0.926112032	2.132258399	18	2076	85	46896	606	UW.CD56_Primary_Cells.ChromatinAc	CD56_Primary_Cells	blood	peaks	Peaks
1.00E-05	3.496863	6.72E-07	1.251866	0.251921386	0.758100232	1.745632064	27	2737	132	46896	866	UW.Mobilized_CD34_Primary_Cells.C	Mobilized_CD34_Primary_	blood	peaks	Peaks
1.00E-05	2.827008	7.89E-07	1.039219	0.210454854	0.62672739	1.451710418	47	6074	132	46896	306	UW.CD4_Primary_Cells.ChromatinAcc	CD4_Primary_Cells	blood	hotspots	Hotspots
1.00E-05	2.814616	8.60E-07	1.034826	0.210278616	0.622679813	1.446971988	52	7093	132	46896	577	UW.Mobilized_CD4_Primary_Cells.Ch	Mobilized_CD4_Primary_(	blood	hotspots	Hotspots
1.00E-08	3.518983	8.70E-07	1.258172	0.255783638	0.756836195	1.759508055	34	6312	85	46896	304	UW.CD4_Primary_Cells.ChromatinAcc	CD4_Primary_Cells	blood	hotspots	Hotspots
1.00E-05	2.88046	9.07E-07	1.05795	0.215429438	0.635708397	1.480191793	43	5161	132	46896	312	UW.CD8_Primary_Cells.ChromatinAcc	CD8_Primary_Cells	blood	hotspots	Hotspots
1.00E-08	4.051923	9.63E-07	1.399192	0.285603052	0.839409628	1.958973591	21	2866	85	46896	963	seg_ENHANCER_gm12878	GM12878	blood	ENHANCER	Chromatin_States
1.00E-05	3.072865	1.00E-06	1.12261	0.229522526	0.672746075	1.572474377	39	4988	132	46896	999	tfbs_gm12878	GM12878	blood	tfbs	TFBS
1.00E-08	3.628496	1.16E-06	1.288818	0.265076488	0.769268415	1.808368248	33	6623	85	46896	567	UW.Mobilized_CD34_Primary_Cells.C	Mobilized_CD34_Primary_	blood	hotspots	Hotspots
1.00E-08	4.508845	1.22E-06	1.506041	0.310360792	0.897733872	2.114348178	19	2099	85	46896	594	UW.CD19_Primary_Cells.ChromatinAc	CD19_Primary_Cells	blood	peaks	Peaks
1.00E-05	3.52137	1.49E-06	1.25885	0.261550369	0.746211359	1.771488805	23	2076	132	46896	606	UW.CD56_Primary_Cells.ChromatinAc	CD56_Primary_Cells	blood	peaks	Peaks
1.00E-08	3.458411	1.53E-06	1.240809	0.258100954	0.734931481	1.746687221	36	7693	85	46896	303	UW.CD4_Primary_Cells.ChromatinAcc	CD4_Primary_Cells	blood	hotspots	Hotspots
1.00E-05	3.516562	1.54E-06	1.257484	0.261673532	0.744603764	1.770364011	23	2075	132	46896	609	UW.CD8_Primary_Cells.ChromatinAcc	CD8_Primary_Cells	blood	peaks	Peaks
1.00E-08	4.715569	1.59E-06	1.55087	0.323111657	0.917570774	2.184168471	17	1742	85	46896	607	UW.CD56_Primary_Cells.ChromatinAc	CD56_Primary_Cells	blood	peaks	Peaks
1.00E-08	0.334851	1.74E-06	-1.094068	0.228828693	-1.54257272	-0.645564242	33	33935	85	46896	975	seg_REPRESSED_gm12878	GM12878	blood	REPRESSED	Chromatin_States
1.00E-08	4.16568	1.78E-06	1.42688	0.298668552	0.841489154	2.012269877	21	3008	85	46896	869	UW.Mobilized_CD34_Primary_Cells.C	Mobilized_CD34_Primary_	blood	peaks	Peaks
1.00E-05	3.426077	1.89E-06	1.231416	0.258429078	0.724894886	1.737936872	26	2508	132	46896	871	UW.Mobilized_CD34_Primary_Cells.C	Mobilized_CD34_Primary_	blood	peaks	Peaks
1.00E-05	2.724895	1.92E-06	1.00243	0.210532341	0.589786528	1.415073304	54	8056	132	46896	578	UW.Mobilized_CD56_Primary_Cells.C	Mobilized_CD56_Primary_	blood	hotspots	Hotspots
1.00E-08	3.52039	1.99E-06	1.258572	0.264735682	0.739689751	1.777453626	31	5550	85	46896	299	UW.CD3_Primary_Cells.ChromatinAcc	CD3_Primary_Cells	blood	hotspots	Hotspots
1.00E-08	4.477188	2.02E-06	1.498995	0.315505733	0.88060404	2.117386512	18	1928	85	46896	599	UW.CD3_Primary_Cells.ChromatinAcc	CD3_Primary_Cells	blood	peaks	Peaks
1.00E-08	4.383297	2.15E-06	1.477801	0.31186407	0.866547655	2.089054811	19	2200	85	46896	177	CD20-DS17541.hg19.hotspot.twopass.	CD20+	blood	peaks	Peaks
1.00E-05	3.516801	2.42E-06	1.257552	0.266725467	0.734769895	1.780333727	23	1928	132	46896	599	UW.CD3_Primary_Cells.ChromatinAcc	CD3_Primary_Cells	blood	peaks	Peaks
1.00E-05	2.802229	2.67E-06	1.030415	0.219470914	0.600252066	1.460578048	47	6901	132	46896	571	UW.Mobilized_CD34_Primary_Cells.C	Mobilized_CD34_Primary_	blood	hotspots	Hotspots
1.00E-08	4.423009	2.80E-06	1.48682	0.317357485	0.864799672	2.108841014	18	1988	85	46896	600	UW.CD3_Primary_Cells.ChromatinAcc	CD3_Primary_Cells	blood	peaks	Peaks
1.00E-05	3.417096	2.94E-06	1.228791	0.262860566	0.713584458	1.743997877	23	2127	132	46896	608	UW.CD8_Primary_Cells.ChromatinAcc	CD8_Primary_Cells	blood	peaks	Peaks
1.00E-05	2.605566	3.07E-06	0.95765	0.205235815	0.555387887	1.359912282	54	7678	132	46896	305	UW.CD4_Primary_Cells.ChromatinAcc	CD4_Primary_Cells	blood	hotspots	Hotspots
1.00E-05	2.67763	3.15E-06	0.984932	0.211336736	0.570712084	1.39915209	51	7131	132	46896	311	UW.CD8_Primary_Cells.ChromatinAcc	CD8_Primary_Cells	blood	hotspots	Hotspots
1.00E-05	2.696974	3.36E-06	0.992131	0.213486017	0.573697914	1.4105631	44	6004	132	46896	902	H2AFZ_gm12878	GM12878	blood	H2AFZ	Histone_Modifications
1.00E-05	3.434304	4.12E-06	1.233814	0.267901985	0.708726491	1.758902273	23	1988	132	46896	600	UW.CD3_Primary_Cells.ChromatinAcc	CD3_Primary_Cells	blood	peaks	Peaks
1.00E-05	2.543653	4.36E-06	0.933601	0.203241707	0.535247543	1.331955066	61	10500	132	46896	60	GM12878-all.twopass.merge150.wgt1	GM12878	blood	hotspots	Hotspots
1.00E-05	3.274664	4.70E-06	1.186215	0.259135297	0.678310012	1.694120376	25	2588	132	46896	865	UW.Mobilized_CD34_Primary_Cells.C	Mobilized_CD34_Primary_	blood	peaks	Peaks
1.00E-05	2.680008	4.81E-06	0.98582	0.215578262	0.563286287	1.408353074	42	5283	132	46896	310	UW.CD8_Primary_Cells.ChromatinAcc	CD8_Primary_Cells	blood	hotspots	Hotspots
1.00E-05	2.640751	4.94E-06	0.971063	0.212604472	0.554358721	1.387768252	51	7711	132	46896	573	UW.Mobilized_CD34_Primary_Cells.C	Mobilized_CD34_Primary_	blood	hotspots	Hotspots
1.00E-05	2.725589	5.35E-06	1.002685	0.220348337	0.570801859	1.43456734	45	6623	132	46896	567	UW.Mobilized_CD34_Primary_Cells.C	Mobilized_CD34_Primary_	blood	hotspots	Hotspots
1.00E-08	3.102529	5.61E-06	1.132217	0.249352756	0.64348605	1.620948855	37	7731	85	46896	302	UW.CD3_Primary_Cells.ChromatinAcc	CD3_Primary_Cells	blood	hotspots	Hotspots
1.00E-05	3.499649	5.84E-06	1.252663	0.276403735	0.710911252	1.794413894	21	1742	132	46896	607	UW.CD56_Primary_Cells.ChromatinAc	CD56_Primary_Cells	blood	peaks	Peaks
1.00E-05	4.024173	6.82E-06	1.392319	0.309459546	0.78577876	1.998860182	16	1021	132	46896	7	GM06990_footprints.txt	GM06990	blood	footprints	Footprints
1.00E-08	3.05282	6.82E-06	1.116066	0.248060603	0.629867069	1.602264632	37	7736	85	46896	576	UW.Mobilized_CD4_Primary_Cells.Ch	Mobilized_CD4_Primary_(	blood	hotspots	Hotspots
1.00E-08	3.789821	7.80E-06	1.332319	0.298020604	0.748198486	1.916439253	21	3354	85	46896	872	UW.Mobilized_CD34_Primary_Cells.C	Mobilized_CD34_Primary_	blood	peaks	Peaks
1.00E-08	4.191192	8.32E-06	1.432985	0.321520224	0.802805649	2.063164929	16	1856	85	46896	611	UW.CD8_Primary_Cells.ChromatinAcc	CD8_Primary_Cells	blood	peaks	Peaks
1.00E-08	4.049946	8.80E-06	1.398703	0.314683299	0.781924233	2.015482766	17	2127	85	46896	608	UW.CD8_Primary_Cells.ChromatinAcc	CD8_Primary_Cells	blood	peaks	Peaks
1.00E-08	3.167885	9.32E-06	1.153064	0.260143903	0.643182177	1.662946277	30	5283	85	46896	310	UW.CD8_Primary_Cells.ChromatinAcc	CD8_Primary_Cells	blood	hotspots	Hotspots
1.00E-08	3.143552	9.77E-06	1.145354	0.258997478	0.637718458	1.652988572	34	7394	85	46896	59	GM12865-DS12436.twopass.merge15	GM12865	blood	hotspots	Hotspots
1.00E-05	3.192881	9.83E-06	1.160924	0.262596001	0.646235517	1.675611841	25	2507	132	46896	870	UW.Mobilized_CD34_Primary_Cells.C	Mobilized_CD34_Primary_	blood	peaks	Peaks
1.00E-08	4.089038	9.94E-06	1.40831	0.318736745	0.783585668	2.033033708	17	2179	85	46896	601	UW.CD3_Primary_Cells.ChromatinAcc	CD3_Primary_Cells	blood	peaks	Peaks
1.00E-05	2.989168	1.06E-05	1.094995	0.248619189	0.607701351	1.582288574	28	3354	132	46896	872	UW.Mobilized_CD34_Primary_Cells.C	Mobilized_CD34_Primary_	blood	peaks	Peaks
1.00E-08	3.119613	1.09E-05	1.137709	0.258700881	0.630655329	1.644762781	31	6004	85	46896	902	H2AFZ_gm12878	GM12878	blood	H2AFZ	Histone_Modifications
1.00E-05	3.154296	1.10E-05	1.148765	0.26125211	0.636711176	1.660819447	24	2505	132	46896	861	UW.Mobilized_CD34_Primary_Cells.C	Mobilized_CD34_Primary_	blood	peaks	Peaks
1.00E-08	3.98425	1.15E-05	1.382349	0.315067841	0.764816175	1.99988211	19	2507	85	46896	870	UW.Mobilized_CD34_Primary_Cells.C	Mobilized_CD34_Primary_	blood	peaks	Peaks
1.00E-08	4.052849	1.16E-05	1.39942	0.319164683	0.773857326	2.024982883	16	2006	85	46896	592	UW.CD14_Primary_Cells.ChromatinAc	CD14_Primary_Cells	blood	peaks	Peaks
1.00E-08	3.191998	1.19E-05	1.160647	0.264995898	0.641255031	1.680038951	30	5948	85	46896	294	UW.CD14_Primary_Cells.ChromatinAc	CD14_Primary_Cells	blood	hotspots	Hotspots
1.00E-08	3.081627	1.23E-05	1.125458	0.257428334	0.620898193	1.63001726	32	6074	85	46896	306	UW.CD4_Primary_Cells.ChromatinAcc	CD4_Primary_Cells	blood	hotspots	Hotspots
1.00E-05	3.401734	1.25E-05	1.224285	0.280219649	0.675054772	1.773515797	19	1705	132	46896	8	GM12865_footprints.txt	GM12865	blood	footprints	Footprints

1.00E-08	3.797927	1.39E-05	1.334455	0.307090978	0.732557119	1.936353753	19	2841	85	46896	868	UW.Mobilized_CD34_Primary_Cells.C	Mobilized_CD34_Primary_	blood	peaks	Peaks
1.00E-05	3.942141	1.42E-05	1.371724	0.316027139	0.752310908	1.991137292	14	1018	132	46896	31	K562_footprints.txt	K562	blood	footprints	Footprints
1.00E-05	3.037208	1.47E-05	1.110939	0.256419423	0.60835673	1.61352087	26	2646	132	46896	874	UW.Mobilized_CD34_Primary_Cells.C	Mobilized_CD34_Primary_	blood	peaks	Peaks
1.00E-08	4.038414	1.56E-05	1.395852	0.323143362	0.762490994	2.029212972	17	1994	85	46896	876	UW.Mobilized_CD4_Primary_Cells.Ch	Mobilized_CD4_Primary_C	blood	peaks	Peaks
1.00E-05	3.23053	1.62E-05	1.172646	0.272010117	0.639506497	1.705786156	22	1994	132	46896	876	UW.Mobilized_CD4_Primary_Cells.Ch	Mobilized_CD4_Primary_C	blood	peaks	Peaks
1.00E-05	3.172167	1.74E-05	1.154415	0.268736805	0.627690808	1.681139084	22	2179	132	46896	601	UW.CD3_Primary_Cells.ChromatinAcc	CD3_Primary_Cells	blood	peaks	Peaks
1.00E-08	3.940794	1.95E-05	1.371382	0.321098786	0.742028604	2.000735846	17	2064	85	46896	878	UW.Mobilized_CD8_Primary_Cells.Ch	Mobilized_CD8_Primary_C	blood	peaks	Peaks
1.00E-08	3.896601	2.02E-05	1.360105	0.319072989	0.734721478	1.985487594	17	1991	85	46896	595	UW.CD19_Primary_Cells.ChromatinAc	CD19_Primary_Cells	blood	peaks	Peaks
1.00E-08	3.034774	2.03E-05	1.110137	0.260499134	0.59955862	1.620715227	31	6186	85	46896	293	UW.CD14_Primary_Cells.ChromatinAc	CD14_Primary_Cells	blood	hotspots	Hotspots
1.00E-05	3.156846	2.03E-05	1.149573	0.269787571	0.620789763	1.678357042	22	1991	132	46896	595	UW.CD19_Primary_Cells.ChromatinAc	CD19_Primary_Cells	blood	peaks	Peaks
1.00E-05	3.157835	2.18E-05	1.149887	0.270827733	0.619064206	1.680708921	22	2064	132	46896	878	UW.Mobilized_CD8_Primary_Cells.Ch	Mobilized_CD8_Primary_C	blood	peaks	Peaks
1.00E-05	2.445067	2.26E-05	0.894072	0.210987482	0.480537002	1.307607933	51	7569	132	46896	579	UW.Mobilized_CD8_Primary_Cells.Ch	Mobilized_CD8_Primary_C	blood	hotspots	Hotspots
1.00E-08	3.068591	2.31E-05	1.121218	0.264900829	0.60201283	1.640424081	33	7299	85	46896	569	UW.Mobilized_CD34_Primary_Cells.C	Mobilized_CD34_Primary_	blood	hotspots	Hotspots
1.00E-08	3.045478	2.46E-05	1.113658	0.263996712	0.596224452	1.631091561	29	5161	85	46896	312	UW.CD8_Primary_Cells.ChromatinAcc	CD8_Primary_Cells	blood	hotspots	Hotspots
1.00E-05	3.082584	2.56E-05	1.125768	0.267438536	0.601588608	1.649947669	24	2200	132	46896	177	CD20-DS17541.hg19.hotspot.twopass.	CD20+	blood	peaks	Peaks
1.00E-05	2.481107	2.77E-05	0.908705	0.216776673	0.483822738	1.333587296	47	6925	132	46896	568	UW.Mobilized_CD34_Primary_Cells.C	Mobilized_CD34_Primary_	blood	hotspots	Hotspots
1.00E-05	3.170422	2.84E-05	1.153865	0.275627645	0.613634519	1.694094887	20	1856	132	46896	611	UW.CD8_Primary_Cells.ChromatinAcc	CD8_Primary_Cells	blood	peaks	Peaks
1.00E-05	2.897559	2.93E-05	1.063869	0.254574201	0.564903294	1.562834161	26	3008	132	46896	869	UW.Mobilized_CD34_Primary_Cells.C	Mobilized_CD34_Primary_	blood	peaks	Peaks
1.00E-05	2.483881	3.10E-05	0.909822	0.218240416	0.481757489	1.337887118	44	6323	132	46896	564	UW.Mobilized_CD34_Primary_Cells.C	Mobilized_CD34_Primary_	blood	hotspots	Hotspots
1.00E-08	3.406526	3.17E-05	1.225693	0.294582816	0.648310699	1.803075339	20	3847	85	46896	949	H3K9me3_gm12878	GM12878	blood	H3K9me3	Histone_Modifications
1.00E-05	2.484563	3.26E-05	0.910097	0.219058846	0.480741306	1.339451983	45	6844	132	46896	565	UW.Mobilized_CD34_Primary_Cells.C	Mobilized_CD34_Primary_	blood	hotspots	Hotspots
1.00E-08	3.562784	3.37E-05	1.270542	0.306358505	0.670079514	1.871004853	19	2889	85	46896	863	UW.Mobilized_CD34_Primary_Cells.C	Mobilized_CD34_Primary_	blood	peaks	Peaks
1.00E-08	3.644707	3.51E-05	1.293276	0.312558776	0.680660675	1.905891077	18	2676	85	46896	873	UW.Mobilized_CD34_Primary_Cells.C	Mobilized_CD34_Primary_	blood	peaks	Peaks
1.00E-08	2.905928	3.73E-05	1.066753	0.258700934	0.559698925	1.573806589	33	7093	85	46896	577	UW.Mobilized_CD4_Primary_Cells.Ch	Mobilized_CD4_Primary_C	blood	hotspots	Hotspots
1.00E-05	2.430102	3.81E-05	0.887933	0.21559912	0.465358856	1.310507406	44	6186	132	46896	293	UW.CD14_Primary_Cells.ChromatinAc	CD14_Primary_Cells	blood	hotspots	Hotspots
1.00E-08	3.511783	4.16E-05	1.256124	0.30649153	0.655400351	1.856847149	20	2956	85	46896	867	UW.Mobilized_CD34_Primary_Cells.C	Mobilized_CD34_Primary_	blood	peaks	Peaks
1.00E-05	3.019702	4.24E-05	1.105158	0.269939158	0.576077427	1.634238927	23	2367	132	46896	877	UW.Mobilized_CD56_Primary_Cells.C	Mobilized_CD56_Primary_	blood	peaks	Peaks
1.00E-05	2.843382	4.77E-05	1.044994	0.256964011	0.541344869	1.548643791	26	3273	132	46896	597	UW.CD20_Primary_Cells.ChromatinAc	CD20_Primary_Cells	blood	peaks	Peaks
1.00E-08	3.439718	5.33E-05	1.235389	0.30573283	0.636153094	1.834625788	19	3023	85	46896	862	UW.Mobilized_CD34_Primary_Cells.C	Mobilized_CD34_Primary_	blood	peaks	Peaks
1.00E-08	3.317611	5.41E-05	1.199245	0.29705236	0.617022379	1.781467632	20	3228	85	46896	184	GM12878-all.fdr0.01.bed	GM12878	blood	peaks	Peaks
1.00E-05	2.999591	5.87E-05	1.098476	0.27339888	0.562614186	1.634337795	21	2006	132	46896	592	UW.CD14_Primary_Cells.ChromatinAc	CD14_Primary_Cells	blood	peaks	Peaks
1.00E-08	3.464063	5.96E-05	1.242442	0.309487027	0.635847621	1.849036767	19	2646	85	46896	874	UW.Mobilized_CD34_Primary_Cells.C	Mobilized_CD34_Primary_	blood	peaks	Peaks
1.00E-08	2.960658	6.14E-05	1.085412	0.270855857	0.554534188	1.616289149	30	6901	85	46896	571	UW.Mobilized_CD34_Primary_Cells.C	Mobilized_CD34_Primary_	blood	hotspots	Hotspots
1.00E-08	2.61319	6.54E-05	0.960572	0.240608656	0.488978593	1.432164524	32	6857	85	46896	917	H3K36me3_gm12878	GM12878	blood	H3K36me3	Histone_Modifications
1.00E-05	2.388197	6.71E-05	0.870539	0.218380453	0.44251315	1.298564524	43	6680	132	46896	574	UW.Mobilized_CD34_Primary_Cells.C	Mobilized_CD34_Primary_	blood	hotspots	Hotspots
1.00E-08	5.011916	7.12E-05	1.611818	0.405778702	0.816492003	2.407144514	8	809	85	46896	950	H3K9me3_k562	K562	blood	H3K9me3	Histone_Modifications
1.00E-05	2.764405	7.73E-05	1.016825	0.257254399	0.512606746	1.521043991	26	2956	132	46896	867	UW.Mobilized_CD34_Primary_Cells.C	Mobilized_CD34_Primary_	blood	peaks	Peaks
1.00E-05	2.998481	9.37E-05	1.098106	0.281116303	0.547117888	1.649093796	20	1909	132	46896	181	GM06990-DS7748.hotspot.twopass.fd	GM06990	blood	peaks	Peaks
1.00E-08	2.755691	0.000107	1.013668	0.261719601	0.500697799	1.526638636	33	7711	85	46896	573	UW.Mobilized_CD34_Primary_Cells.C	Mobilized_CD34_Primary_	blood	hotspots	Hotspots
1.00E-05	2.227052	0.000114	0.800679	0.207495477	0.393987619	1.207369889	48	7558	132	46896	52	CD14-DS18065.hg19.twopass.merge15	CD14+	blood	hotspots	Hotspots
1.00E-08	3.505464	0.000116	1.254323	0.325422562	0.616494794	1.892151236	17	2367	85	46896	877	UW.Mobilized_CD56_Primary_Cells.C	Mobilized_CD56_Primary_	blood	peaks	Peaks
1.00E-05	3.884399	0.000123	1.356968	0.353344175	0.664413599	2.049522764	10	809	132	46896	950	H3K9me3_k562	K562	blood	H3K9me3	Histone_Modifications
1.00E-05	2.340628	0.000124	0.850419	0.221571752	0.416138751	1.284700019	40	5948	132	46896	294	UW.CD14_Primary_Cells.ChromatinAc	CD14_Primary_Cells	blood	hotspots	Hotspots
1.00E-05	2.221945	0.000126	0.798383	0.208186029	0.390338177	1.206427409	51	8474	132	46896	54	CD34-DS16814.hg19.twopass.merge15	CD34+	blood	hotspots	Hotspots
1.00E-08	2.716421	0.000128	0.999315	0.260840122	0.488068458	1.510561736	32	7131	85	46896	311	UW.CD8_Primary_Cells.ChromatinAcc	CD8_Primary_Cells	blood	hotspots	Hotspots
1.00E-05	2.752965	0.00013	1.012678	0.264661275	0.493942256	1.531414456	23	2676	132	46896	873	UW.Mobilized_CD34_Primary_Cells.C	Mobilized_CD34_Primary_	blood	peaks	Peaks
1.00E-05	2.307683	0.000145	0.836244	0.220099999	0.404847942	1.267639938	46	7299	132	46896	569	UW.Mobilized_CD34_Primary_Cells.C	Mobilized_CD34_Primary_	blood	hotspots	Hotspots
1.00E-05	2.498936	0.000151	0.915865	0.241697952	0.442136924	1.389592895	33	5102	132	46896	901	FAIRE_k562	K562	blood	faire	FAIRE
1.00E-08	3.106416	0.000156	1.13347	0.299773619	0.545913238	1.721025824	18	2868	85	46896	262	wgEncodeOpenChromDnaseGm12892	GM12892	blood	peaks	Peaks
1.00E-05	3.033204	0.000157	1.109619	0.293570937	0.534220327	1.685018401	17	1600	132	46896	598	UW.CD3_Primary_Cells.ChromatinAcc	CD3_Primary_Cells	blood	peaks	Peaks
1.00E-08	3.435118	0.000158	1.234051	0.326642529	0.593831885	1.874270599	15	2090	85	46896	602	UW.CD4_Primary_Cells.ChromatinAcc	CD4_Primary_Cells	blood	peaks	Peaks
1.00E-05	2.260499	0.000168	0.815585	0.21674045	0.390774216	1.240396782	46	7394	132	46896	59	GM12865-DS12436.twopass.merge15	GM12865	blood	hotspots	Hotspots
1.00E-08	2.642056	0.000171	0.971557	0.258478301	0.464939855	1.478174797	33	7569	85	46896	579	UW.Mobilized_CD8_Primary_Cells.Ch	Mobilized_CD8_Primary_C	blood	hotspots	Hotspots
1.00E-08	3.533536	0.000184	1.262299	0.33749818	0.600802759	1.923795625	15	1909	85	46896	181	GM06990-DS7748.hotspot.twopass.fd	GM06990	blood	peaks	Peaks
1.00E-08	4.038668	0.000187	1.395915	0.373672978	0.663515827	2.128313901	11	1216	85	46896	969	seg_PROMOTER_FLANK_gm12878	GM12878	blood	PROMOTER_FLANK	Chromatin_States
1.00E-05	2.617721	0.000214	0.962304	0.259960826	0.452781023	1.47182746	24	2889	132	46896	863	UW.Mobilized_CD34_Primary_Cells.C	Mobilized_CD34_Primary_	blood	peaks	Peaks
1.00E-05	2.100302	0.000228	0.742081	0.201308933	0.347515793	1.13664681	42	6857	132	46896	917	H3K36me3_gm12878	GM12878	blood	H3K36me3	Histone_Modifications
1.00E-08	2.608675	0.000246	0.958843	0.261548551	0.446207437	1.471477759	33	8056	85	46896	578	UW.Mobilized_CD56_Primary_Cells.C	Mobilized_CD56_Primary_	blood	hotspots	Hotspots

1.00E-08	3.269056	0.000247	1.184501	0.323134014	0.551158735	1.817844069	16	2476	85	46896	179	CMK-DS12393.hotspot.twopass.fdr0.0	CMK	blood	peaks	Peaks
1.00E-05	3.216064	0.000254	1.168158	0.319297586	0.542334971	1.793981508	14	1216	132	46896	969	seg_PROMOTER_FLANK_gm12878	GM12878	blood	PROMOTER_FLANK	Chromatin_States
1.00E-08	3.497356	0.000256	1.252007	0.342441665	0.580821551	1.923192878	13	1705	85	46896	8	GM12865_footprints.txt	GM12865	blood	footprints	Footprints
1.00E-08	2.649252	0.000264	0.974277	0.267043801	0.450871569	1.497683269	30	6680	85	46896	574	UW.Mobilized_CD34_Primary_Cells.C	Mobilized_CD34_Primary_	blood	hotspots	Hotspots
1.00E-05	2.608954	0.000287	0.958949	0.264385236	0.440754281	1.477144406	23	2841	132	46896	868	UW.Mobilized_CD34_Primary_Cells.C	Mobilized_CD34_Primary_	blood	peaks	Peaks
1.00E-08	2.65113	0.000299	0.974986	0.269606341	0.446557694	1.503414551	30	6844	85	46896	565	UW.Mobilized_CD34_Primary_Cells.C	Mobilized_CD34_Primary_	blood	hotspots	Hotspots
1.00E-08	3.28141	0.000309	1.188273	0.329383998	0.542680463	1.833865737	16	2148	85	46896	875	UW.Mobilized_CD4_Primary_Cells.Ch	Mobilized_CD4_Primary_(	blood	peaks	Peaks
1.00E-08	3.941198	0.000319	1.371485	0.381070259	0.624586989	2.118382404	11	1021	85	46896	7	GM06990_footprints.txt	GM06990	blood	footprints	Footprints
1.00E-08	2.645187	0.000325	0.972742	0.270633545	0.442299898	1.503183395	30	6990	85	46896	566	UW.Mobilized_CD34_Primary_Cells.C	Mobilized_CD34_Primary_	blood	hotspots	Hotspots
1.00E-05	2.516932	0.000373	0.923041	0.259400694	0.414615413	1.431466132	24	3023	132	46896	862	UW.Mobilized_CD34_Primary_Cells.C	Mobilized_CD34_Primary_	blood	peaks	Peaks
1.00E-05	2.194339	0.000386	0.785881	0.221424586	0.351888741	1.219873119	42	6636	132	46896	572	UW.Mobilized_CD34_Primary_Cells.C	Mobilized_CD34_Primary_	blood	hotspots	Hotspots
1.00E-05	2.729799	0.000419	1.004228	0.284644063	0.446325498	1.562130225	18	1889	132	46896	604	UW.CD4_Primary_Cells.ChromatinAcc	CD4_Primary_Cells	blood	peaks	Peaks
1.00E-08	2.446039	0.000432	0.89447	0.254130188	0.396374653	1.392564988	33	7678	85	46896	305	UW.CD4_Primary_Cells.ChromatinAcc	CD4_Primary_Cells	blood	hotspots	Hotspots
1.00E-05	2.593031	0.000446	0.952827	0.271363549	0.420954937	1.484700047	22	2476	132	46896	179	CMK-DS12393.hotspot.twopass.fdr0.0	CMK	blood	peaks	Peaks
1.00E-08	3.186797	0.000468	1.159016	0.331314468	0.509639963	1.808392677	15	2266	85	46896	263	wgEncodeOpenChromDnaseGm18507	GM18507	blood	peaks	Peaks
1.00E-08	2.598903	0.000469	0.955089	0.273042451	0.41992624	1.490252648	29	6784	85	46896	562	UW.Mobilized_CD34_Primary_Cells.C	Mobilized_CD34_Primary_	blood	hotspots	Hotspots
1.00E-08	2.550939	0.00049	0.936462	0.268642221	0.409922774	1.463000279	29	6531	85	46896	570	UW.Mobilized_CD34_Primary_Cells.C	Mobilized_CD34_Primary_	blood	hotspots	Hotspots
1.00E-08	2.552835	0.000496	0.937204	0.269087214	0.409793496	1.464615374	29	6323	85	46896	564	UW.Mobilized_CD34_Primary_Cells.C	Mobilized_CD34_Primary_	blood	hotspots	Hotspots
1.00E-05	2.634629	0.000516	0.968742	0.278991021	0.421919895	1.515564699	21	2090	132	46896	602	UW.CD4_Primary_Cells.ChromatinAcc	CD4_Primary_Cells	blood	peaks	Peaks
1.00E-08	3.065388	0.000561	1.120174	0.32471554	0.483731738	1.756616655	16	2711	85	46896	183	GM12865-DS12436.hotspot.twopass.f	GM12865	blood	peaks	Peaks
1.00E-05	2.395122	0.000564	0.873434	0.253284599	0.376996269	1.369871897	26	3228	132	46896	184	GM12878-all.fdr0.01.bed	GM12878	blood	peaks	Peaks
1.00E-05	2.083562	0.000592	0.734079	0.213690761	0.315244957	1.152912738	47	7644	132	46896	575	UW.Mobilized_CD34_Primary_Cells.C	Mobilized_CD34_Primary_	blood	hotspots	Hotspots
1.00E-08	2.500593	0.000619	0.916528	0.267749702	0.391738565	1.441317397	30	6925	85	46896	568	UW.Mobilized_CD34_Primary_Cells.C	Mobilized_CD34_Primary_	blood	hotspots	Hotspots
1.00E-05	2.149097	0.00062	0.765048	0.223526724	0.326935202	1.203159959	42	6990	132	46896	566	UW.Mobilized_CD34_Primary_Cells.C	Mobilized_CD34_Primary_	blood	hotspots	Hotspots
1.00E-05	2.130778	0.000643	0.756487	0.221651379	0.322050325	1.19092373	41	6531	132	46896	570	UW.Mobilized_CD34_Primary_Cells.C	Mobilized_CD34_Primary_	blood	hotspots	Hotspots
1.00E-05	2.623202	0.000665	0.964396	0.283329553	0.409069891	1.51972174	18	1970	132	46896	968	seg_ENHANCER_k562	K562	blood	ENHANCER	Chromatin_States
1.00E-05	2.150278	0.000675	0.765597	0.225206497	0.324192384	1.207001851	41	6784	132	46896	562	UW.Mobilized_CD34_Primary_Cells.C	Mobilized_CD34_Primary_	blood	hotspots	Hotspots
1.00E-05	2.527078	0.000714	0.927064	0.27395621	0.390109709	1.464018053	21	2528	132	46896	864	UW.Mobilized_CD34_Primary_Cells.C	Mobilized_CD34_Primary_	blood	peaks	Peaks
1.00E-08	2.971187	0.000724	1.088962	0.32214893	0.457549797	1.720373604	16	2742	85	46896	238	NB4-DS12543.hotspot.twopass.fdr0.0	NB4	blood	peaks	Peaks
1.00E-08	3.203477	0.000771	1.164237	0.346190028	0.485704277	1.842769187	14	1834	85	46896	610	UW.CD8_Primary_Cells.ChromatinAcc	CD8_Primary_Cells	blood	peaks	Peaks
1.00E-08	3.287783	0.000823	1.190213	0.355816102	0.492813904	1.887613025	12	1600	85	46896	598	UW.CD3_Primary_Cells.ChromatinAcc	CD3_Primary_Cells	blood	peaks	Peaks
1.00E-08	2.988464	0.000848	1.094759	0.328093573	0.451696076	1.737822881	16	2945	85	46896	233	Jurkat-DS12659.hotspot.twopass.fdr0.	Jurkat	blood	peaks	Peaks
1.00E-08	2.33555	0.000899	0.848247	0.255461529	0.347542832	1.348952027	34	8474	85	46896	54	CD34-DS16814.hg19.twopass.merge1	CD34+	blood	hotspots	Hotspots
1.00E-05	2.325369	0.000905	0.843879	0.254300967	0.345448989	1.342308779	24	3847	132	46896	949	H3K9me3_gm12878	GM12878	blood	H3K9me3	Histone_Modifications
1.00E-05	2.80616	0.000929	1.031817	0.311615551	0.421050503	1.642583462	14	1243	132	46896	2	CD20+_footprints.txt	CD20+	blood	footprints	Footprints
1.00E-08	3.688427	0.00093	1.3052	0.394201782	0.532564543	2.077835527	9	1018	85	46896	31	K562_footprints.txt	K562	blood	footprints	Footprints
1.00E-05	2.616199	0.001029	0.961722	0.292997101	0.387448	1.535996637	18	1834	132	46896	610	UW.CD8_Primary_Cells.ChromatinAcc	CD8_Primary_Cells	blood	peaks	Peaks
1.00E-08	2.425091	0.001042	0.885869	0.270167417	0.356340733	1.415397006	29	6705	85	46896	563	UW.Mobilized_CD34_Primary_Cells.C	Mobilized_CD34_Primary_	blood	hotspots	Hotspots
1.00E-08	3.06956	0.001055	1.121534	0.342396107	0.450437989	1.792630729	13	1889	85	46896	604	UW.CD4_Primary_Cells.ChromatinAcc	CD4_Primary_Cells	blood	peaks	Peaks
1.00E-05	2.42554	0.001163	0.886054	0.27282279	0.351321715	1.420787051	21	2711	132	46896	183	GM12865-DS12436.hotspot.twopass.f	GM12865	blood	peaks	Peaks
1.00E-08	2.808378	0.001264	1.032607	0.320275746	0.404866612	1.660347538	17	3273	85	46896	597	UW.CD20_Primary_Cells.ChromatinA	CD20_Primary_Cells	blood	peaks	Peaks
1.00E-05	2.355938	0.001337	0.856939	0.267138643	0.333347167	1.380530649	22	2846	132	46896	205	HL60-DS11809.hotspot.twopass.fdr0.(	HL-60	blood	peaks	Peaks
1.00E-08	2.975869	0.001351	1.090536	0.340261203	0.423624297	1.757448212	13	2002	85	46896	32	NB4_footprints.txt	NB4	blood	footprints	Footprints
1.00E-05	2.139275	0.001466	0.760467	0.239036149	0.29195609	1.228977794	31	4967	132	46896	227	hTH1-all.fdr0.01.bed	Th1	blood	peaks	Peaks
1.00E-08	2.781172	0.001521	1.022872	0.322603313	0.390569858	1.655174845	16	2846	85	46896	205	HL60-DS11809.hotspot.twopass.fdr0.(	HL-60	blood	peaks	Peaks
1.00E-08	2.280028	0.001576	0.824188	0.260799092	0.313021439	1.335353881	32	8156	85	46896	138	wgEncodeOpenChromDnaseGm18507	GM18507	blood	hotspots	Hotspots
1.00E-05	2.019766	0.001607	0.702982	0.22284381	0.266207685	1.139755422	42	6705	132	46896	563	UW.Mobilized_CD34_Primary_Cells.C	Mobilized_CD34_Primary_	blood	hotspots	Hotspots
1.00E-08	2.833365	0.001707	1.041465	0.331995363	0.390754026	1.692175849	15	2528	85	46896	864	UW.Mobilized_CD34_Primary_Cells.C	Mobilized_CD34_Primary_	blood	peaks	Peaks
1.00E-08	3.042236	0.00175	1.112593	0.355490216	0.415831961	1.809353606	12	1882	85	46896	228	hTH2-DS7842.hotspot.twopass.fdr0.0	Th2	blood	peaks	Peaks
1.00E-05	2.400445	0.001881	0.875654	0.28170196	0.323518135	1.42778982	20	2266	132	46896	263	wgEncodeOpenChromDnaseGm18507	GM18507	blood	peaks	Peaks
1.00E-08	2.66129	0.001955	0.978811	0.316045193	0.359362313	1.598259469	17	2819	85	46896	264	wgEncodeOpenChromDnaseGm19238	GM19238	blood	peaks	Peaks
1.00E-08	2.319301	0.002002	0.841266	0.272256616	0.30764267	1.374888604	28	6636	85	46896	572	UW.Mobilized_CD34_Primary_Cells.C	Mobilized_CD34_Primary_	blood	hotspots	Hotspots
1.00E-05	2.515231	0.002019	0.922365	0.298753668	0.336807652	1.507922032	16	1882	132	46896	228	hTH2-DS7842.hotspot.twopass.fdr0.0	Th2	blood	peaks	Peaks
1.00E-08	2.205575	0.002064	0.790988	0.256744685	0.287768783	1.294207948	31	7558	85	46896	52	CD14-DS18065.hg19.twopass.merge1	CD14+	blood	hotspots	Hotspots
1.00E-05	2.457736	0.002554	0.899241	0.298071263	0.315020965	1.483460316	16	1848	132	46896	3	CD34+_Mobilized_footprints.txt	CD34+	blood	footprints	Footprints
1.00E-08	2.900803	0.002607	1.064988	0.353738475	0.371660158	1.75831498	12	1848	85	46896	3	CD34+_Mobilized_footprints.txt	CD34+	blood	footprints	Footprints
1.00E-05	2.335746	0.003005	0.848332	0.285904669	0.287958373	1.408704676	19	2148	132	46896	875	UW.Mobilized_CD4_Primary_Cells.Ch	Mobilized_CD4_Primary_(	blood	peaks	Peaks
1.00E-08	3.033387	0.003045	1.10968	0.374497484	0.375664618	1.843694755	10	1243	85	46896	2	CD20+_footprints.txt	CD20+	blood	footprints	Footprints

1.00E-08	3.119682	0.00356	1.137731	0.390342087	0.372660693	1.902801674	9	1446	85	46896	993	seg_WEAK_ENHANCER_gm12878	GM12878	blood	WEAK_ENHANCER	Chromatin_States
1.00E-08	2.560238	0.003941	0.9401	0.326098608	0.300946833	1.579253378	16	3040	85	46896	178	CD34-DS16814.hg19.hotspot.twopass.	CD34+	blood	peaks	Peaks
1.00E-05	1.857504	0.003991	0.619234	0.215095088	0.197647334	1.040820077	45	8156	132	46896	138	wgEncodeOpenChromDnaseGm18507	GM18507	blood	hotspots	Hotspots
1.00E-08	3.038498	0.00425	1.111363	0.388731003	0.349450454	1.873275985	9	1166	85	46896	40	Th1_footprints.txt	Th1	blood	footprints	Footprints
1.00E-05	2.168931	0.00437	0.774235	0.271650801	0.24179897	1.306670112	22	3357	132	46896	962	seg_CTCF_k562	K562	blood	CTCF	Chromatin_States
1.00E-05	2.134901	0.004459	0.75842	0.266700455	0.235687581	1.281153363	23	2819	132	46896	264	wgEncodeOpenChromDnaseGm19238	GM19238	blood	peaks	Peaks
1.00E-08	2.55301	0.004553	0.937273	0.330362823	0.289761833	1.584784099	15	3357	85	46896	962	seg_CTCF_k562	K562	blood	CTCF	Chromatin_States
1.00E-05	1.833159	0.004774	0.606041	0.214762315	0.185106587	1.026974863	41	7454	132	46896	81	HL60-DS11809.twopass.merge150.wg	HL-60	blood	hotspots	Hotspots
1.00E-05	2.074396	0.005497	0.72967	0.262812263	0.214558044	1.244782113	21	2868	132	46896	262	wgEncodeOpenChromDnaseGm12892	GM12892	blood	peaks	Peaks
1.00E-08	2.622228	0.006384	0.964024	0.353463064	0.271236835	1.656812046	11	1970	85	46896	968	seg_ENHANCER_k562	K562	blood	ENHANCER	Chromatin_States
1.00E-05	2.027868	0.009797	0.706985	0.273717396	0.170498997	1.243471188	21	3040	132	46896	178	CD34-DS16814.hg19.hotspot.twopass.	CD34+	blood	peaks	Peaks
1.00E-05	1.745023	0.009925	0.556768	0.215932583	0.133540121	0.979995845	43	8137	132	46896	132	wgEncodeOpenChromDnaseCllAlnAllR	CLL	blood	hotspots	Hotspots
1.00E-08	2.196159	0.010757	0.78671	0.308452828	0.182142286	1.39127737	20	5102	85	46896	901	FAIRE_k562	K562	blood	faire	FAIRE
1.00E-05	2.028537	0.011406	0.707315	0.279571213	0.159355271	1.255274425	19	2742	132	46896	238	NB4-DS12543.hotspot.twopass.fdr0.0	NB4	blood	peaks	Peaks
1.00E-08	2.499419	0.01171	0.916058	0.36340405	0.203786184	1.628330059	13	2243	85	46896	896	FAIRE_gm12878	GM12878	blood	faire	FAIRE
1.00E-05	2.356355	0.011942	0.857116	0.340957448	0.188839235	1.525392431	11	1166	132	46896	40	Th1_footprints.txt	Th1	blood	footprints	Footprints
1.00E-08	1.9418	0.012483	0.663615	0.265637622	0.142965582	1.184265058	29	7644	85	46896	575	UW.Mobilized_CD34_Primary_Cells.C	Mobilized_CD34_Primary_	blood	hotspots	Hotspots
1.00E-08	2.099755	0.013116	0.741821	0.299047622	0.155687295	1.327953973	20	4967	85	46896	227	hTH1-all.fdr0.01.bed	Th1	blood	peaks	Peaks
1.00E-05	1.583317	0.01321	0.459522	0.185436936	0.09606585	0.822978639	53	12300	132	46896	916	H3K27me3_k562	K562	blood	H3K27me3	Histone_Modifications
1.00E-05	1.741139	0.013673	0.554539	0.224897823	0.11373964	0.995339106	38	6421	132	46896	104	hTH2-DS7842.twopass.merge150.wgt1	Th2	blood	hotspots	Hotspots
1.00E-08	1.916032	0.014144	0.650256	0.265022521	0.130812039	1.169700321	29	8137	85	46896	132	wgEncodeOpenChromDnaseCllAlnAllR	CLL	blood	hotspots	Hotspots
1.00E-05	1.987072	0.015357	0.686662	0.283293206	0.131407275	1.241916643	20	2945	132	46896	233	Jurkat-DS12659.hotspot.twopass.fdr0.	Jurkat	blood	peaks	Peaks
1.00E-05	2.073571	0.016505	0.729272	0.304172932	0.133093231	1.325451125	18	2243	132	46896	896	FAIRE_gm12878	GM12878	blood	faire	FAIRE
1.00E-08	2.142929	0.016753	0.762174	0.318622907	0.137672891	1.386674687	17	3669	85	46896	167	Adult_Th0_AllReps.30000000.hotspot	Adult_CD4+	blood	peaks	Peaks
1.00E-05	2.04235	0.018095	0.714101	0.30211498	0.12195597	1.306246693	15	2002	132	46896	32	NB4_footprints.txt	NB4	blood	footprints	Footprints
1.00E-08	2.612066	0.018471	0.960141	0.407524927	0.161392454	1.758890169	7	1042	85	46896	951	H4K20me1_gm12878	GM12878	blood	H4K20me1	Histone_Modifications
1.00E-05	1.690772	0.01922	0.525185	0.2243196	0.085518917	0.96485175	37	7327	132	46896	113	NB4-DS12543.twopass.merge150.wgt	NB4	blood	hotspots	Hotspots
1.00E-08	1.84612	0.021059	0.613086	0.265759847	0.092196639	1.13397524	27	7454	85	46896	81	HL60-DS11809.twopass.merge150.wg	HL-60	blood	hotspots	Hotspots
1.00E-08	1.799296	0.021263	0.587396	0.255025851	0.08754497	1.087246305	34	10501	85	46896	141	wgEncodeOpenChromDnaseGm19240	GM19240	blood	hotspots	Hotspots
1.00E-08	1.78768	0.022297	0.580919	0.254200781	0.082685249	1.079152309	33	9664	85	46896	139	wgEncodeOpenChromDnaseGm19238	GM19238	blood	hotspots	Hotspots
1.00E-05	1.672985	0.02239	0.51461	0.225341575	0.072940138	0.956279111	37	6689	132	46896	55	CMK-DS12393.twopass.merge150.wgt	CMK	blood	hotspots	Hotspots
1.00E-05	1.597333	0.02428	0.468335	0.207901877	0.060847379	0.875822738	39	6996	132	46896	948	H3K9ac_k562	K562	blood	H3K9ac	Histone_Modifications
1.00E-05	2.208122	0.025945	0.792142	0.355694209	0.094981781	1.489303079	9	1042	132	46896	951	H4K20me1_gm12878	GM12878	blood	H4K20me1	Histone_Modifications
1.00E-05	1.704512	0.027434	0.533279	0.241818123	0.059315284	1.007242326	31	5966	132	46896	1004	tfbs_k562	K562	blood	tfbs	TFBS
1.00E-05	1.683514	0.027868	0.520883	0.236858578	0.056640605	0.98512623	30	5007	132	46896	938	H3K4me3_k562	K562	blood	H3K4me3	Histone_Modifications
1.00E-08	1.80881	0.031445	0.592669	0.275481083	0.052726402	1.132612248	68	35838	85	46896	980	seg_REPRESSED_k562	K562	blood	REPRESSED	Chromatin_States
1.00E-08	1.834207	0.042128	0.606612	0.298492032	0.021567733	1.191656499	21	5966	85	46896	1004	tfbs_k562	K562	blood	tfbs	TFBS
1.00E-08	2.125805	0.042582	0.754151	0.371908689	0.025209571	1.483091633	11	1890	85	46896	257	wgEncodeOpenChromDnaseCllAlnAllR	CLL	blood	peaks	Peaks
1.00E-08	1.744788	0.045791	0.556633	0.278690626	0.01039966	1.102866913	24	6421	85	46896	104	hTH2-DS7842.twopass.merge150.wgt1	Th2	blood	hotspots	Hotspots
1.00E-05	2.025905	0.045978	0.706017	0.353787822	0.01259249	1.399440753	11	1446	132	46896	993	seg_WEAK_ENHANCER_gm12878	GM12878	blood	WEAK_ENHANCER	Chromatin_States
1.00E-05	1.697537	0.048568	0.529178	0.268295879	0.003318297	1.055038144	23	3669	132	46896	167	Adult_Th0_AllReps.30000000.hotspot	Adult_CD4+	blood	peaks	Peaks
1.00E-08	1.975395	0.049304	0.680768	0.346279334	0.002060821	1.359475811	12	2216	85	46896	992	seg_TSS_k562	K562	blood	TSS	Chromatin_States
1.00E-05	1.50483	0.050677	0.40868	0.209128987	-0.001212561	0.818573068	47	9664	132	46896	139	wgEncodeOpenChromDnaseGm19238	GM19238	blood	hotspots	Hotspots
1.00E-08	1.902452	0.052838	0.643143	0.332161013	-0.007892169	1.294179001	14	3246	85	46896	266	wgEncodeOpenChromDnaseGm19240	GM19240	blood	peaks	Peaks
1.00E-05	1.734553	0.060972	0.55075	0.293937698	-0.025367954	1.126867821	16	2216	132	46896	992	seg_TSS_k562	K562	blood	TSS	Chromatin_States
1.00E-05	1.720962	0.064153	0.542883	0.293274202	-0.031934086	1.117700786	17	2455	132	46896	265	wgEncodeOpenChromDnaseGm19239	GM19239	blood	peaks	Peaks
1.00E-05	1.474395	0.064477	0.388248	0.209992645	-0.023337579	0.79983359	48	10501	132	46896	141	wgEncodeOpenChromDnaseGm19240	GM19240	blood	hotspots	Hotspots
1.00E-08	1.666817	0.067183	0.510916	0.279120326	-0.036159847	1.05799183	24	7327	85	46896	113	NB4-DS12543.twopass.merge150.wgt	NB4	blood	hotspots	Hotspots
1.00E-05	1.450102	0.071403	0.371634	0.206130621	-0.032382074	0.775649961	50	10885	132	46896	42	Adult_Th0_AllReps.30000000.twopass	Adult_CD4+	blood	hotspots	Hotspots
1.00E-05	1.755635	0.076644	0.562831	0.317893804	-0.060241138	1.185902574	14	1890	132	46896	257	wgEncodeOpenChromDnaseCllAlnAllR	CLL	blood	peaks	Peaks
1.00E-05	1.48637	0.077373	0.396337	0.224409311	-0.043505315	0.836179184	42	8802	132	46896	298	UW.CD20_Primary_Cells.ChromatinAc	CD20_Primary_Cells	blood	hotspots	Hotspots
1.00E-05	1.62072	0.081849	0.482871	0.277502774	-0.061034711	1.026776164	20	3246	132	46896	266	wgEncodeOpenChromDnaseGm19240	GM19240	blood	peaks	Peaks
1.00E-05	1.440211	0.087124	0.36479	0.213231515	-0.053143983	0.782723558	47	10046	132	46896	103	hTH1-all.twopass.merge150.wgt10.zgt	Th1	blood	hotspots	Hotspots
1.00E-05	1.487807	0.089736	0.397303	0.234149682	-0.061630201	0.856236551	27	5059	132	46896	922	H3K36me3_k562	K562	blood	H3K36me3	Histone_Modifications
1.00E-08	1.746608	0.093175	0.557676	0.332170505	-0.093378601	1.20872978	11	2780	85	46896	911	H3K27me3_gm12878	GM12878	blood	H3K27me3	Histone_Modifications
1.00E-08	1.798715	0.100386	0.587073	0.357320167	-0.113274998	1.287420056	11	2455	85	46896	265	wgEncodeOpenChromDnaseGm19239	GM19239	blood	peaks	Peaks
1.00E-08	1.597555	0.111371	0.468475	0.294256484	-0.108268149	1.045217267	17	4611	85	46896	942	H3K79me2_k562	K562	blood	H3K79me2	Histone_Modifications
1.00E-05	1.617722	0.11629	0.481019	0.306276369	-0.119282627	1.081320738	16	2865	132	46896	957	seg_CTCF_gm12878	GM12878	blood	CTCF	Chromatin_States

1.00E-05	1.542422	0.124293	0.433354	0.281948099	-0.119264089	0.985972461	19	3366	132	46896	234	K562-all.fdr0.01.bed	K562	blood	peaks	Peaks
1.00E-08	1.488521	0.13286	0.397783	0.264673774	-0.120977673	0.916543523	28	8712	85	46896	140	wgEncodeOpenChromDnaseGm1923§	GM19239	blood	hotspots	Hotspots
1.00E-08	1.532393	0.133419	0.426831	0.284410748	-0.130614508	0.984275623	24	7724	85	46896	109	Jurkat-DS12659.twopass.merge150.w;	Jurkat	blood	hotspots	Hotspots
1.00E-05	1.353608	0.13969	0.302773	0.204999765	-0.099026138	0.704572941	46	9865	132	46896	137	wgEncodeOpenChromDnaseGm12892	GM12892	blood	hotspots	Hotspots
1.00E-08	1.464255	0.147881	0.381347	0.263532597	-0.135177236	0.897870546	30	10046	85	46896	103	hTH1-all.twopass.merge150.wgt10.zgt	Th1	blood	hotspots	Hotspots
1.00E-08	1.391191	0.152957	0.33016	0.231016584	-0.122632236	0.782952773	32	12300	85	46896	916	H3K27me3_k562	K562	blood	H3K27me3	Histone_Modifications
1.00E-08	1.663006	0.153605	0.508627	0.356452441	-0.190019717	1.207273854	12	2623	85	46896	261	wgEncodeOpenChromDnaseGm12891	GM12891	blood	peaks	Peaks
1.00E-05	1.422626	0.154193	0.352504	0.247392756	-0.13238552	0.837394082	25	4681	132	46896	910	H3K27ac_k562	K562	blood	H3K27ac	Histone_Modifications
1.00E-08	1.685861	0.168212	0.522276	0.379017649	-0.220598367	1.265150819	11	2865	85	46896	957	seg_CTCF_gm12878	GM12878	blood	CTCF	Chromatin_States
1.00E-05	1.346774	0.169325	0.297712	0.216616496	-0.126856162	0.722280504	41	8712	132	46896	140	wgEncodeOpenChromDnaseGm1923§	GM19239	blood	hotspots	Hotspots
1.00E-05	1.370629	0.178159	0.31527	0.234149541	-0.143663565	0.774202638	30	5846	132	46896	932	H3K4me2_k562	K562	blood	H3K4me2	Histone_Modifications
1.00E-05	1.36598	0.180441	0.311872	0.232844964	-0.144503861	0.768248397	35	7724	132	46896	109	Jurkat-DS12659.twopass.merge150.w;	Jurkat	blood	hotspots	Hotspots
1.00E-08	1.477698	0.189221	0.390486	0.297425185	-0.192467824	0.9734389	18	5007	85	46896	938	H3K4me3_k562	K562	blood	H3K4me3	Histone_Modifications
1.00E-05	1.459049	0.206718	0.377785	0.299202312	-0.208651466	0.964221595	16	2623	132	46896	261	wgEncodeOpenChromDnaseGm12891	GM12891	blood	peaks	Peaks
1.00E-08	1.366067	0.217393	0.311936	0.252888373	-0.183725643	0.807596779	30	9865	85	46896	137	wgEncodeOpenChromDnaseGm12892	GM12892	blood	hotspots	Hotspots
1.00E-08	1.428026	0.218568	0.356293	0.289587683	-0.21129901	0.923884707	17	5059	85	46896	922	H3K36me3_k562	K562	blood	H3K36me3	Histone_Modifications
1.00E-08	1.421716	0.219145	0.351864	0.286346767	-0.209375246	0.913104081	21	6689	85	46896	55	CMK-DS12393.twopass.merge150.wgt	CMK	blood	hotspots	Hotspots
1.00E-05	1.293051	0.222049	0.257004	0.210470104	-0.155517098	0.669525708	44	9527	132	46896	136	wgEncodeOpenChromDnaseGm12891	GM12891	blood	hotspots	Hotspots
1.00E-05	1.416791	0.23007	0.348394	0.290285199	-0.220564654	0.917353327	14	2780	132	46896	911	H3K27me3_gm12878	GM12878	blood	H3K27me3	Histone_Modifications
1.00E-05	1.301841	0.239938	0.263779	0.224465989	-0.176173923	0.703732752	32	7041	132	46896	904	H2AFZ_k562	K562	blood	H2AFZ	Histone_Modifications
1.00E-05	1.313836	0.278082	0.272951	0.251651864	-0.220286545	0.766188763	23	4611	132	46896	942	H3K79me2_k562	K562	blood	H3K79me2	Histone_Modifications
1.00E-08	1.439066	0.305424	0.363994	0.35516114	-0.332121643	1.060110027	12	3366	85	46896	234	K562-all.fdr0.01.bed	K562	blood	peaks	Peaks
1.00E-05	1.232961	0.309504	0.209419	0.206067622	-0.194473782	0.613311295	98	35838	132	46896	980	seg_REPRESSED_k562	K562	blood	REPRESSED	Chromatin_States
1.00E-08	1.322378	0.323874	0.279432	0.283247195	-0.275732914	0.83459609	25	8802	85	46896	298	UW.CD20_Primary_Cells.ChromatinAc	CD20_Primary_Cells	blood	hotspots	Hotspots
1.00E-08	2.739298	0.327876	1.007702	1.029947343	-1.01099496	3.026398624	1	137	85	46896	974	seg_PROMOTER_FLANK_k562	K562	blood	PROMOTER_FLANK	Chromatin_States
1.00E-08	1.622409	0.368807	0.483912	0.538452413	-0.571454368	1.539279089	4	959	85	46896	998	seg_WEAK_ENHANCER_k562	K562	blood	WEAK_ENHANCER	Chromatin_States
1.00E-05	1.186412	0.393493	0.170934	0.200321231	-0.221695653	0.563563572	53	14540	132	46896	981	seg_TRANSCRIBED_gm12878	GM12878	blood	TRANSCRIBED	Chromatin_States
1.00E-08	0.810409	0.404984	-0.210217	0.252435994	-0.704991213	0.284557886	25	11592	85	46896	926	H3K4me1_k562	K562	blood	H3K4me1	Histone_Modifications
1.00E-08	1.235278	0.415135	0.211296	0.25929325	-0.2969191	0.719510442	30	10885	85	46896	42	Adult_Th0_AllReps.30000000.twopass	Adult_CD4+	blood	hotspots	Hotspots
1.00E-08	1.299632	0.472349	0.262081	0.364678294	-0.452688657	0.976850256	9	2861	85	46896	956	H4K20me1_k562	K562	blood	H4K20me1	Histone_Modifications
1.00E-05	1.802556	0.563861	0.589206	1.020948173	-1.411852814	2.590264025	1	137	132	46896	974	seg_PROMOTER_FLANK_k562	K562	blood	PROMOTER_FLANK	Chromatin_States
1.00E-08	1.141077	0.618828	0.131972	0.265264391	-0.387946041	0.651890371	26	9527	85	46896	136	wgEncodeOpenChromDnaseGm12891	GM12891	blood	hotspots	Hotspots
1.00E-08	1.162844	0.635339	0.150868	0.318136044	-0.472678228	0.774415066	14	4681	85	46896	910	H3K27ac_k562	K562	blood	H3K27ac	Histone_Modifications
1.00E-05	0.904231	0.648091	-0.100671	0.220567416	-0.532982694	0.331641576	32	9879	132	46896	986	seg_TRANSCRIBED_k562	K562	blood	TRANSCRIBED	Chromatin_States
1.00E-05	1.087462	0.699034	0.083847	0.21686886	-0.341216207	0.508909726	44	11448	132	46896	110	K562-all.twopass.merge150.wgt10.zgt	K562	blood	hotspots	Hotspots
1.00E-08	1.112775	0.72138	0.106857	0.29964116	-0.480440013	0.694153334	17	5846	85	46896	932	H3K4me2_k562	K562	blood	H3K4me2	Histone_Modifications
1.00E-08	1.083738	0.768033	0.080416	0.272642681	-0.453963899	0.614795411	20	6996	85	46896	948	H3K9ac_k562	K562	blood	H3K9ac	Histone_Modifications
1.00E-05	0.906411	0.770932	-0.098263	0.337492015	-0.759747074	0.563221625	11	2861	132	46896	956	H4K20me1_k562	K562	blood	H4K20me1	Histone_Modifications
1.00E-08	0.942987	0.817643	-0.058702	0.254588334	-0.557695615	0.440290656	30	14540	85	46896	981	seg_TRANSCRIBED_gm12878	GM12878	blood	TRANSCRIBED	Chromatin_States
1.00E-08	0.939662	0.817859	-0.062235	0.270234169	-0.591893617	0.467424325	21	9879	85	46896	986	seg_TRANSCRIBED_k562	K562	blood	TRANSCRIBED	Chromatin_States
1.00E-08	1.06008	0.829122	0.058345	0.270329677	-0.471501474	0.58819086	28	11448	85	46896	110	K562-all.twopass.merge150.wgt10.zgt	K562	blood	hotspots	Hotspots
1.00E-05	0.97571	0.902164	-0.02459	0.200033842	-0.416655996	0.367476667	44	11592	132	46896	926	H3K4me1_k562	K562	blood	H3K4me1	Histone_Modifications
1.00E-08	0.975237	0.931938	-0.025075	0.293593425	-0.600517882	0.550368346	17	7041	85	46896	904	H2AFZ_k562	K562	blood	H2AFZ	Histone_Modifications
1.00E-05	1.008677	0.986859	0.00864	0.524548064	-1.019474293	1.036754118	4	959	132	46896	998	seg_WEAK_ENHANCER_k562	K562	blood	WEAK_ENHANCER	Chromatin_States

PThresh - GWAS threshold used for enrichment analysis testing
OR - Odds ratio for annotation at that threshold
Pvalue – P-value of the significance of the observed enrichment
Beta - Effect size (or log odds ratio) for that annotation at that threshold
SE - Standard error of the effect size for that annotation at that threshold
CI95 - 95% CI of the effect size
NAnnotThresh - Number of (independent) annotated variants with the considered annotation passing the GWAS significance threshold PThresh (after pruning)
NAnnot - Total number of (independent) annotated variants with the given annot
Nthresh - Number of (independent) variants passing the GWAS significance threshold PThresh (after pruning)
N - Total number of LD pruned variants
linkID - ID of the annotation file
Annotation - Unique name for the annotation
Celltype - Cell type of the annotation

Tissue - Tissue of the annotation
Type - Subtype of the annotation
Category – The category of the annotation
