# Supplementary material for: Identification of Novel Associations and Localization of Signals in Idiopathic Inflammatory Myopathies Using Genome‐Wide Imputation
Source: Arthritis Rheumatol. 2023 Mar 20;75(6):1021–7. doi: 10.1002/art.42434 (PMC10238560; doi:10.1002/art.42434)
Supplement: Supplementary file 4 — Figure S1: Supplementary Figure [file ART-75-1021-s001.docx]

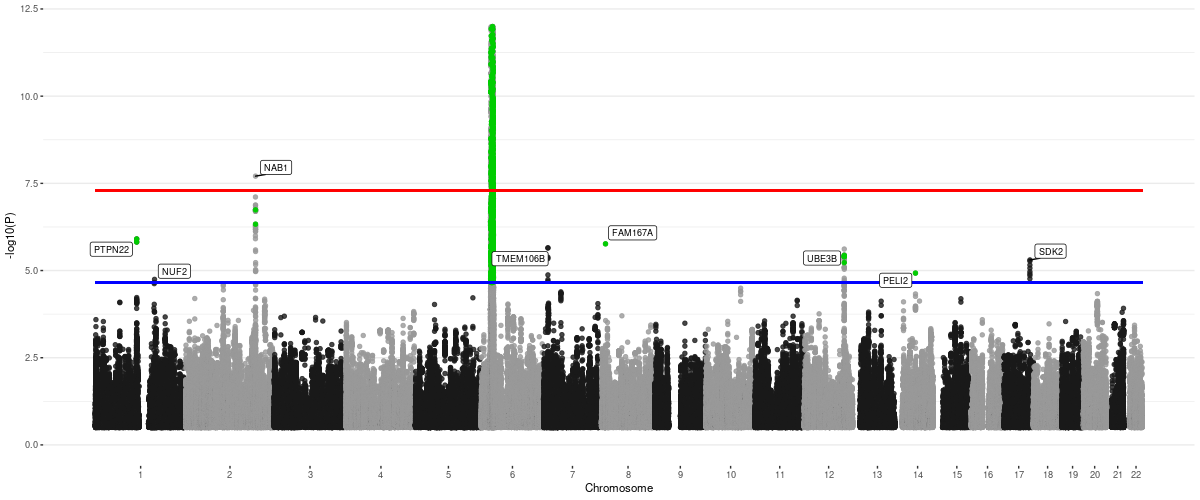


Supplementary Figure 1. Manhattan plot of the PM (n=903) association analysis. Red line represents genome‐wide level of significance (p < 5× 10^−8^); blue line represents suggestive significance (p < 2.25 × 10^−5^). SNPs reaching P < 2.25 × 10^−5^ that were directly genotyped are coloured in green to differentiate between imputed variants. For visualisation purposes the Y axis has a cut-off in the HLA region (chromosome 6 25–35Mb) of p < 1 x 10^-12^


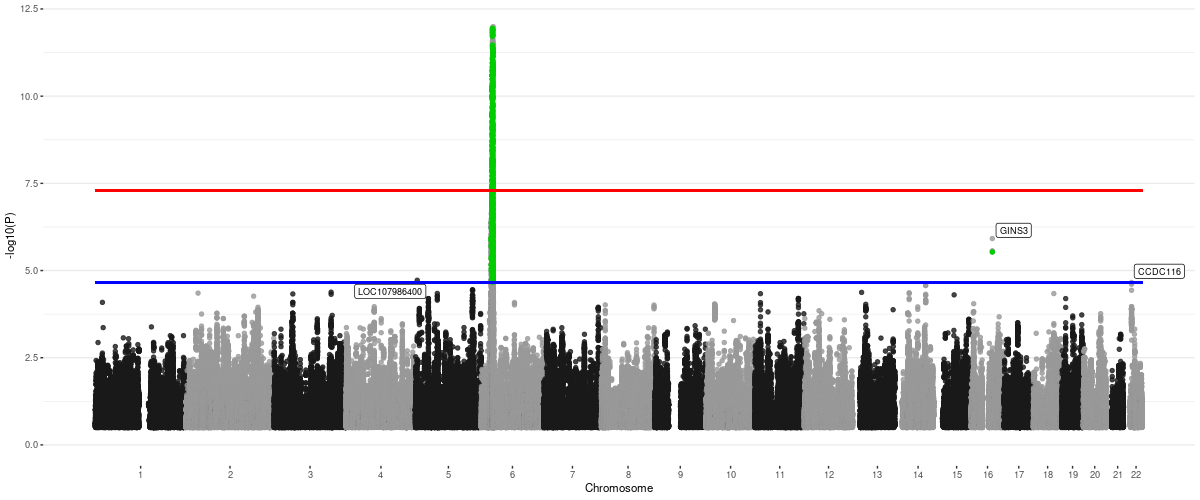


Supplementary Figure 2. Manhattan plot of the DM (n=817) association analysis. Red line represents genome‐wide level of significance (p < 5× 10^−8^); blue line represents suggestive significance (p < 2.25 × 10^−5^). SNPs reaching P < 2.25 × 10^−5^ that were directly genotyped are coloured in green to differentiate between imputed variants. For visualisation purposes the Y axis has a cut-off in the HLA region (chromosome 6 25–35Mb) of p < 1 x 10^-12^


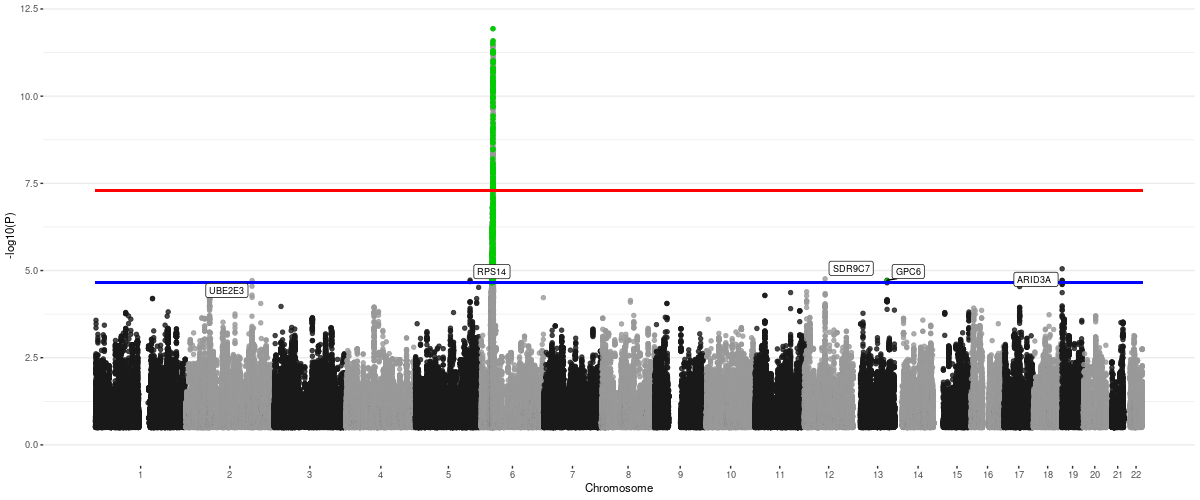


Supplementary Figure 3. Manhattan plot of the JDM (n=508) association analysis. Red line represents genome‐wide level of significance (p < 5× 10^−8^); blue line represents suggestive significance (p < 2.25 × 10^−5^). SNPs reaching P < 2.25 × 10^−5^ that were directly genotyped are coloured in green to differentiate between imputed variants. For visualisation purposes the Y axis has a cut-off in the HLA region (chromosome 6 25–35Mb) of p < 1 x 10^-12^


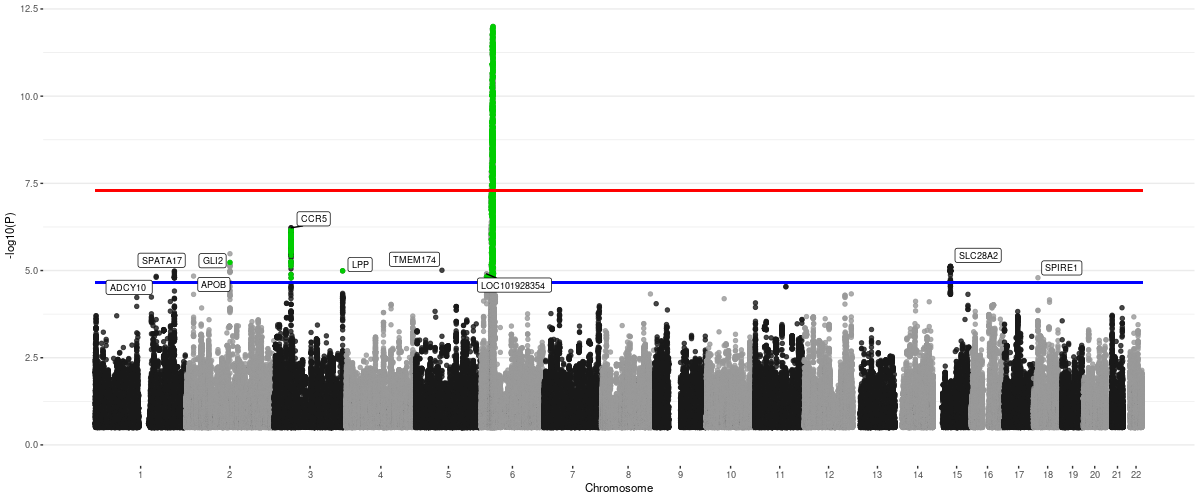


Supplementary Figure 4. Manhattan plot of the IBM (n=252) association analysis. Red line represents genome‐wide level of significance (p < 5× 10^−8^); blue line represents suggestive significance (p < 2.25 × 10^−5^). SNPs reaching P < 2.25 × 10^−5^ that were directly genotyped are coloured in green to differentiate between imputed variants. For visualisation purposes the Y axis has a cut-off in the HLA region (chromosome 6 25–35Mb) of p < 1 x 10^-12^


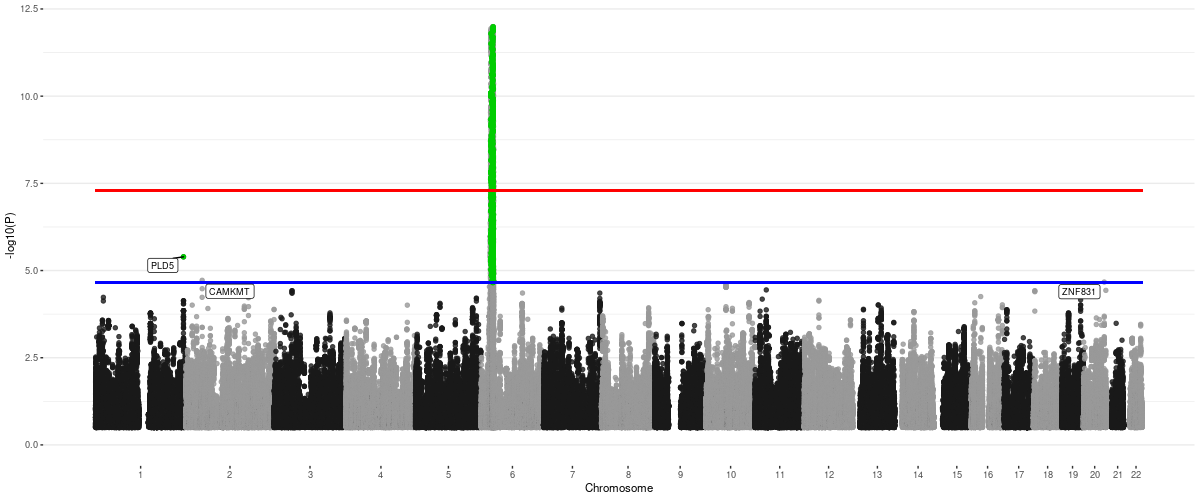


Supplementary Figure 5. Manhattan plot of the IIM anti-Jo1 positive (n=311) association analysis. Red line represents genome‐wide level of significance (p < 5× 10^−8^); blue line represents suggestive significance (p < 2.25 × 10^−5^). SNPs reaching P < 2.25 × 10^−5^ that were directly genotyped are coloured in green to differentiate between imputed variants. For visualisation purposes the Y axis has a cut-off in the HLA region (chromosome 6 25–35Mb) of p < 1 x 10^-12^


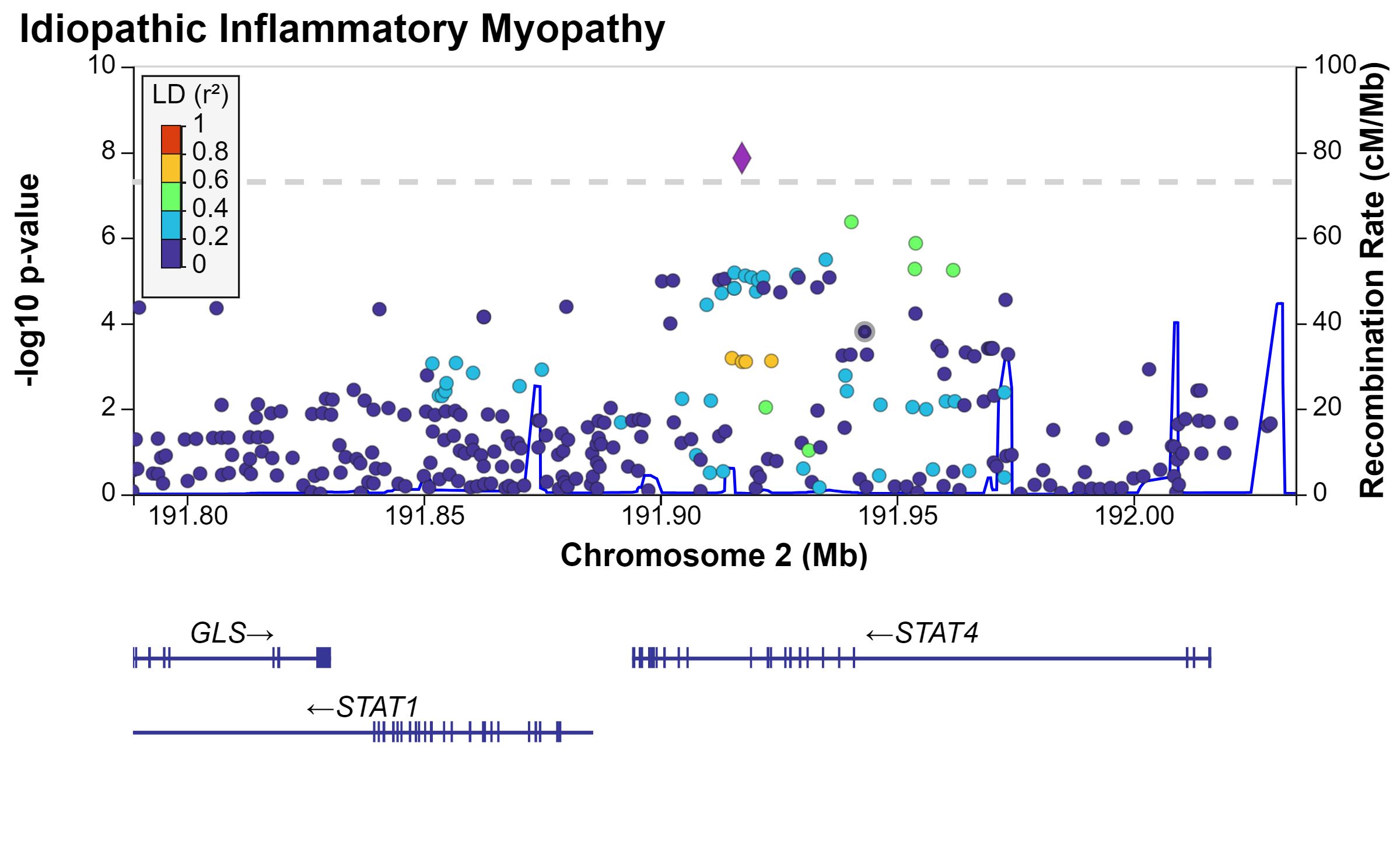


Supplementary Figure 6. Regional association plot of the STAT4 locus in IIM. The most associated SNP in the region is identified with the purple diamond (rs4853540). SNPs are colour coded by the degree of linkage disequilibrium with the index SNP.


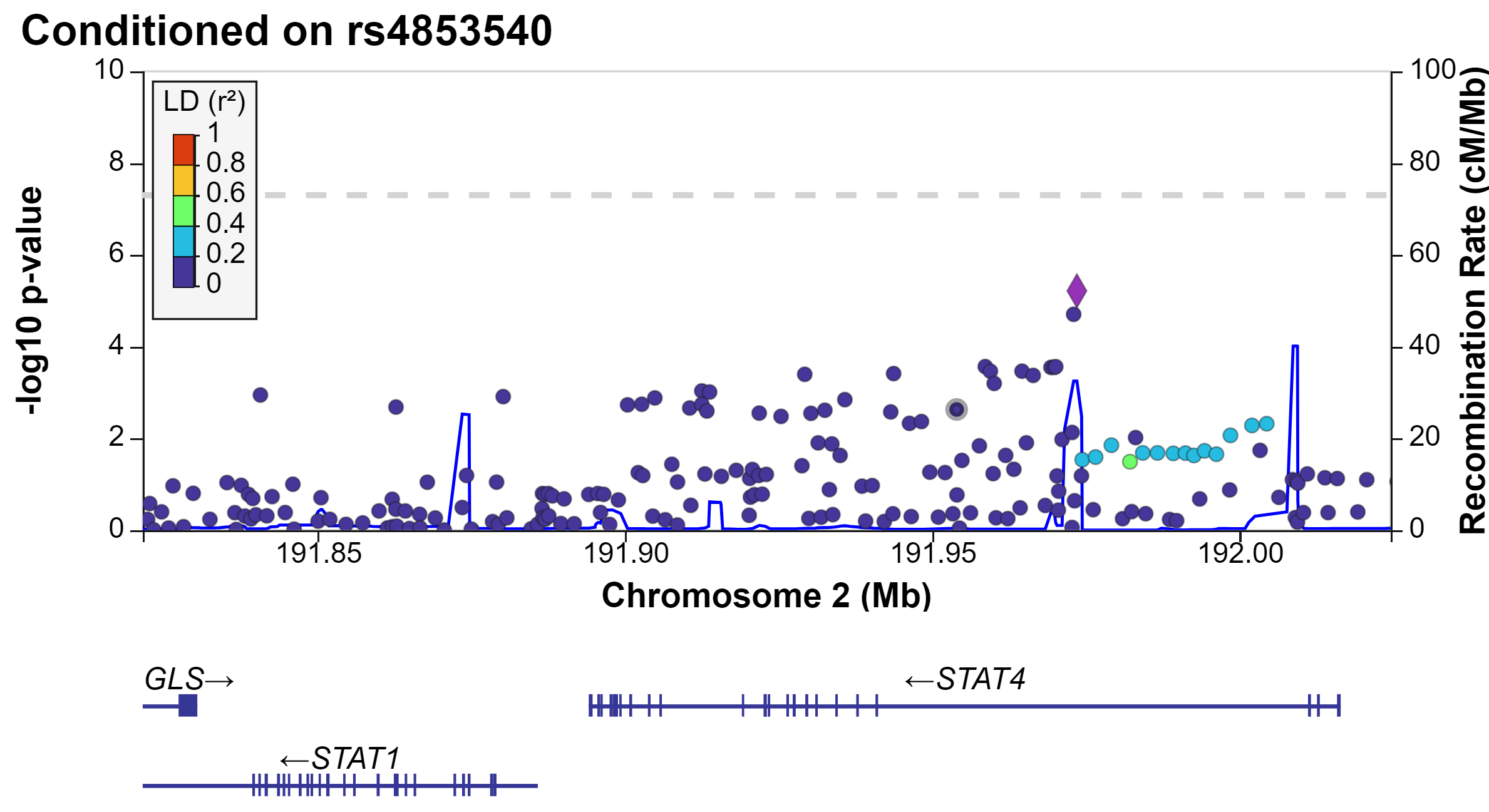


Supplementary Figure 7. Regional association plot of the STAT4 locus in IIM, after conditioning on rs4853540. The next most associated SNP in the region is identified with the purple diamond (rs6752770). SNPs are colour coded by the degree of linkage disequilibrium with the index SNP.


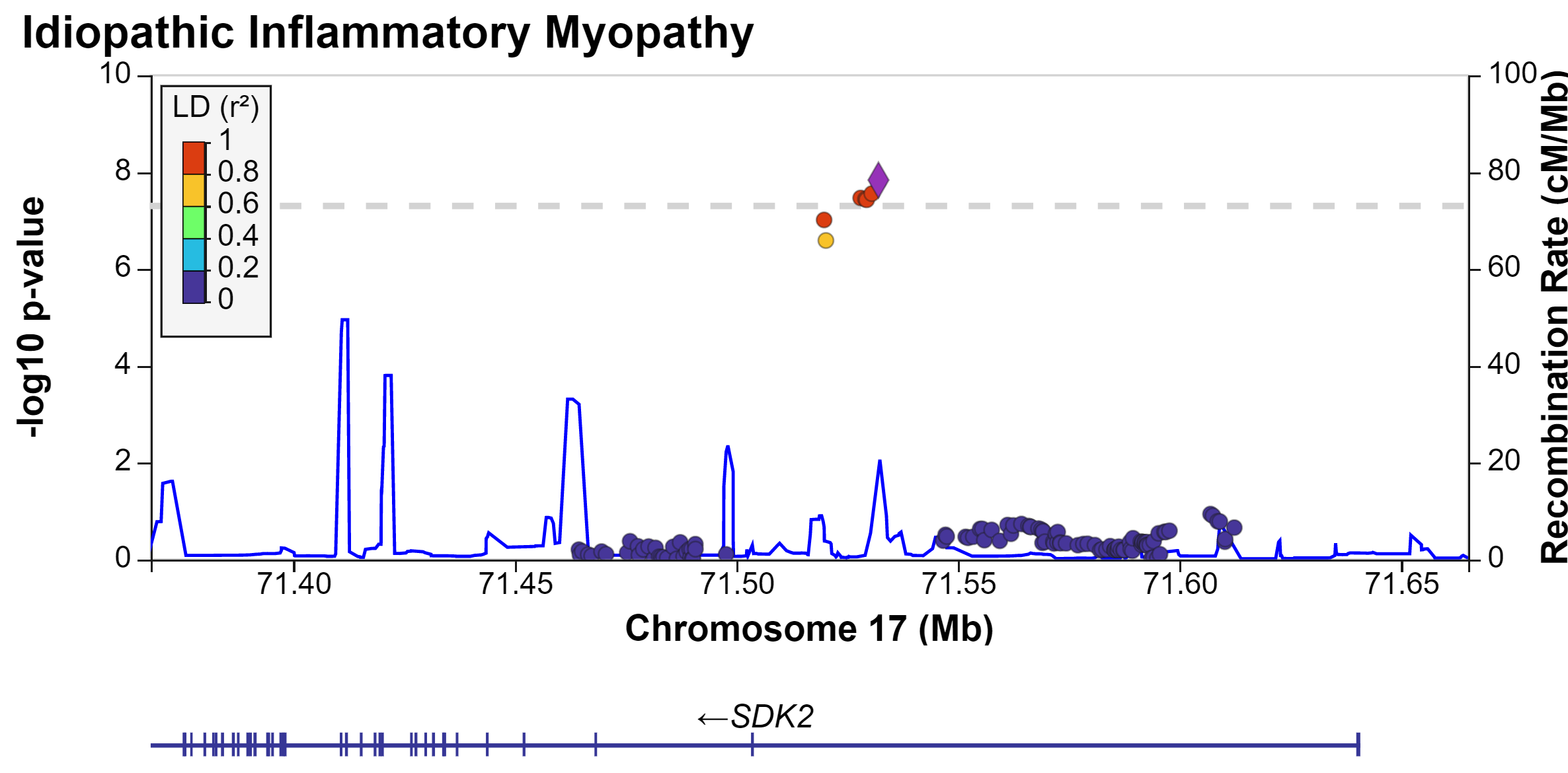


Supplementary Figure 8. Regional association plot of the SDK2 locus in IIM. The most associated SNP in the region is identified with the purple diamond (rs7209879). SNPs are colour coded by the degree of linkage disequilibrium with the index SNP.


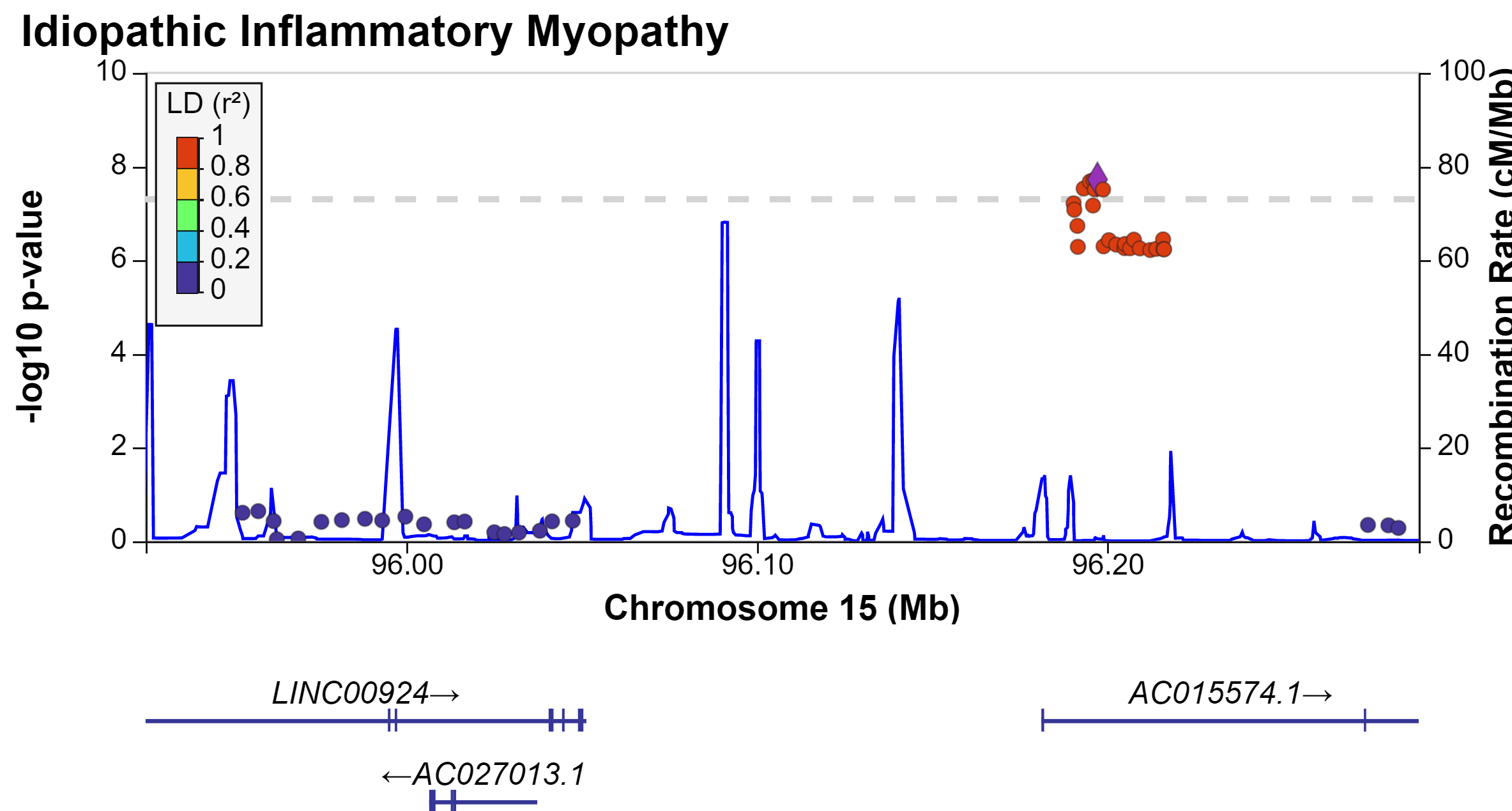


Supplementary Figure 9. Regional association plot of the LINC00924 locus in IIM. The most associated SNP in the region is identified with the purple diamond (rs8040452). SNPs are colour coded by the degree of linkage disequilibrium with the index SNP.


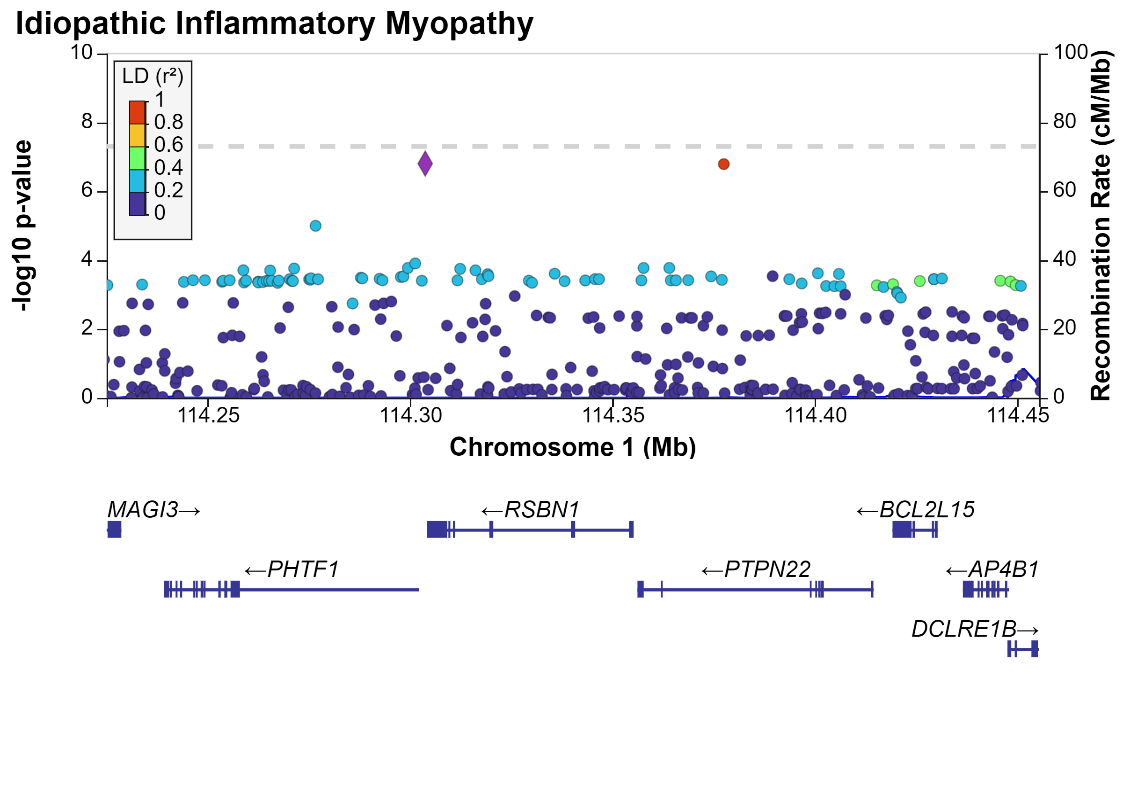


Supplementary Figure 10. Regional association plot of the PHTF1-PTPN22 locus in IIM. The most associated SNP in the region is identified with the purple diamond (rs6679677). SNPs are colour coded by the degree of linkage disequilibrium with the index SNP.


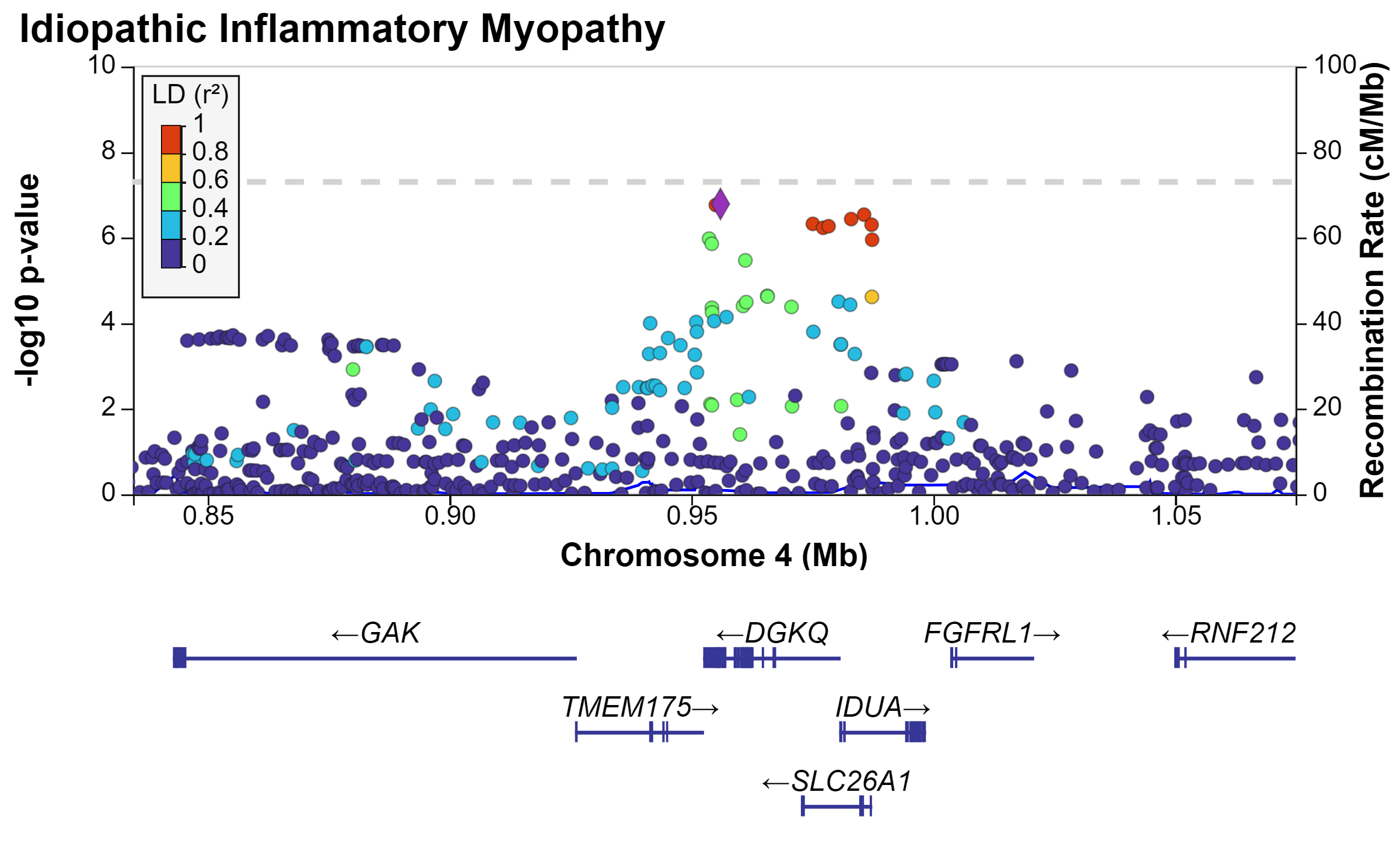


Supplementary Figure 11. Regional association plot of the DGKQ locus in IIM. The most associated SNP in the region is identified with the purple diamond (rs6599390). SNPs are colour coded by the degree of linkage disequilibrium with the index SNP.


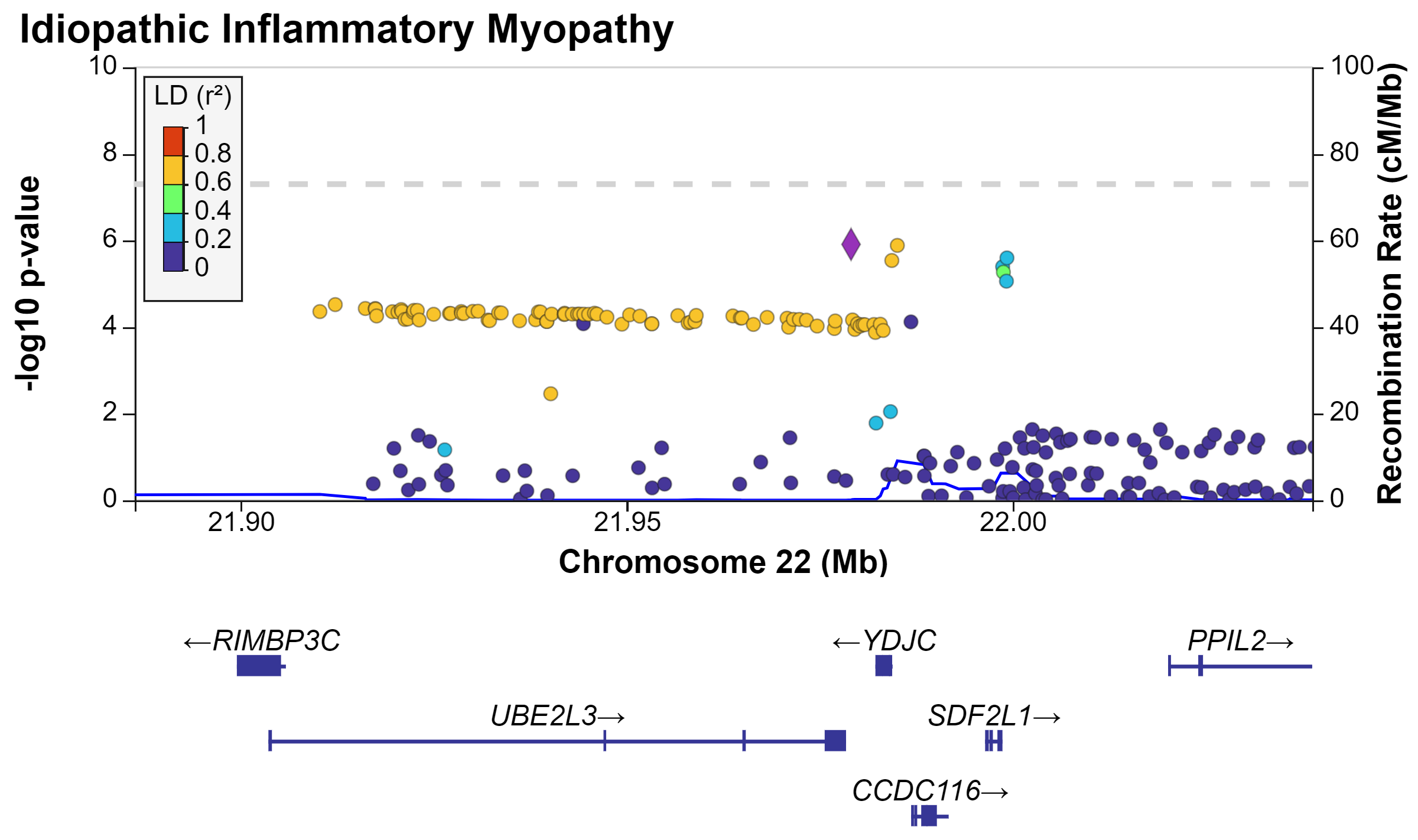


Supplementary Figure 12. Regional association plot of the UBE2L3-YDJC locus in IIM. The most associated SNP in the region is identified with the purple diamond (rs11089637). SNPs are colour coded by the degree of linkage disequilibrium with the index SNP.


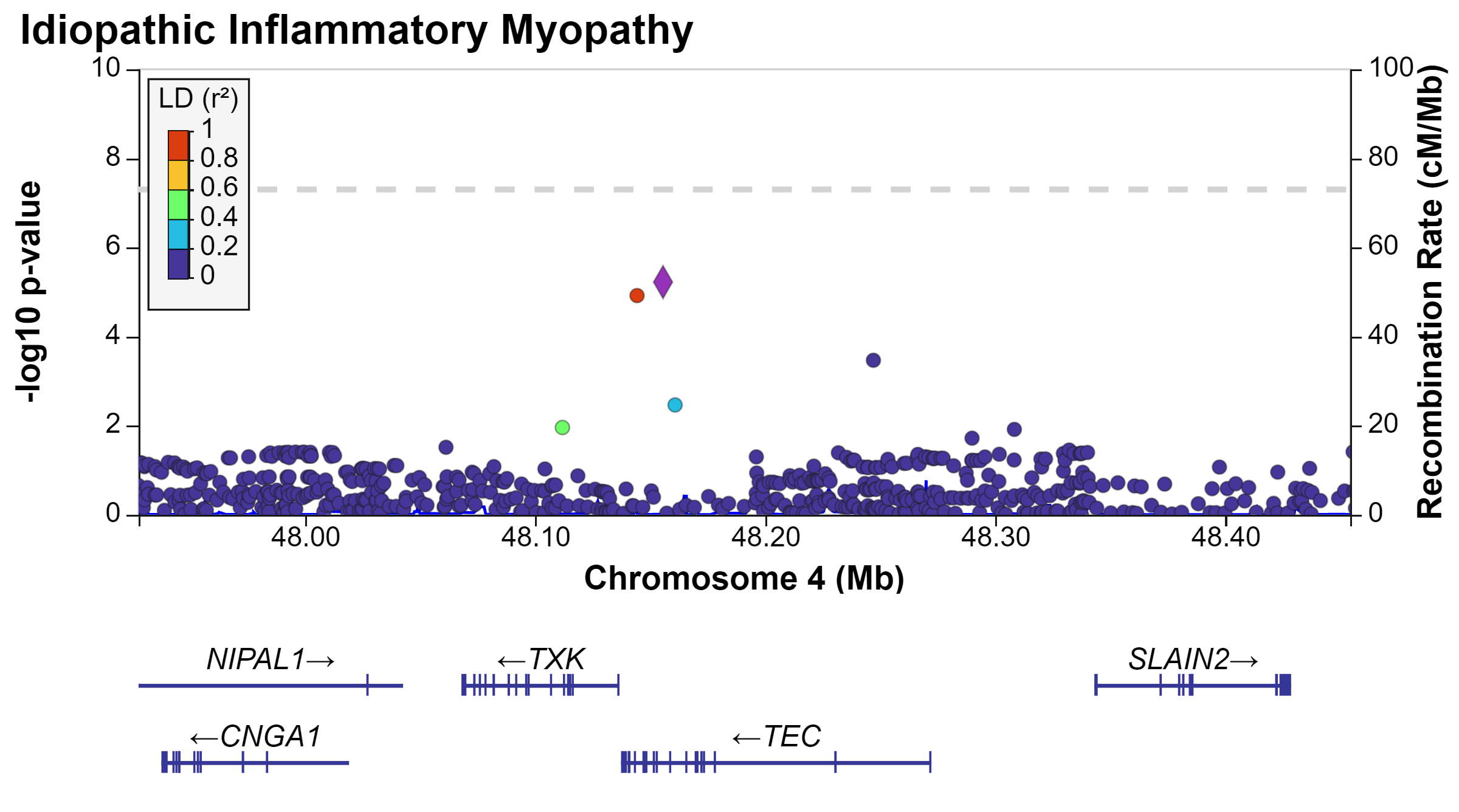


Supplementary Figure 13. Regional association plot of the TEC locus in IIM. The most associated SNP in the region is identified with the purple diamond (rs80105690). SNPs are colour coded by the degree of linkage disequilibrium with the index SNP.


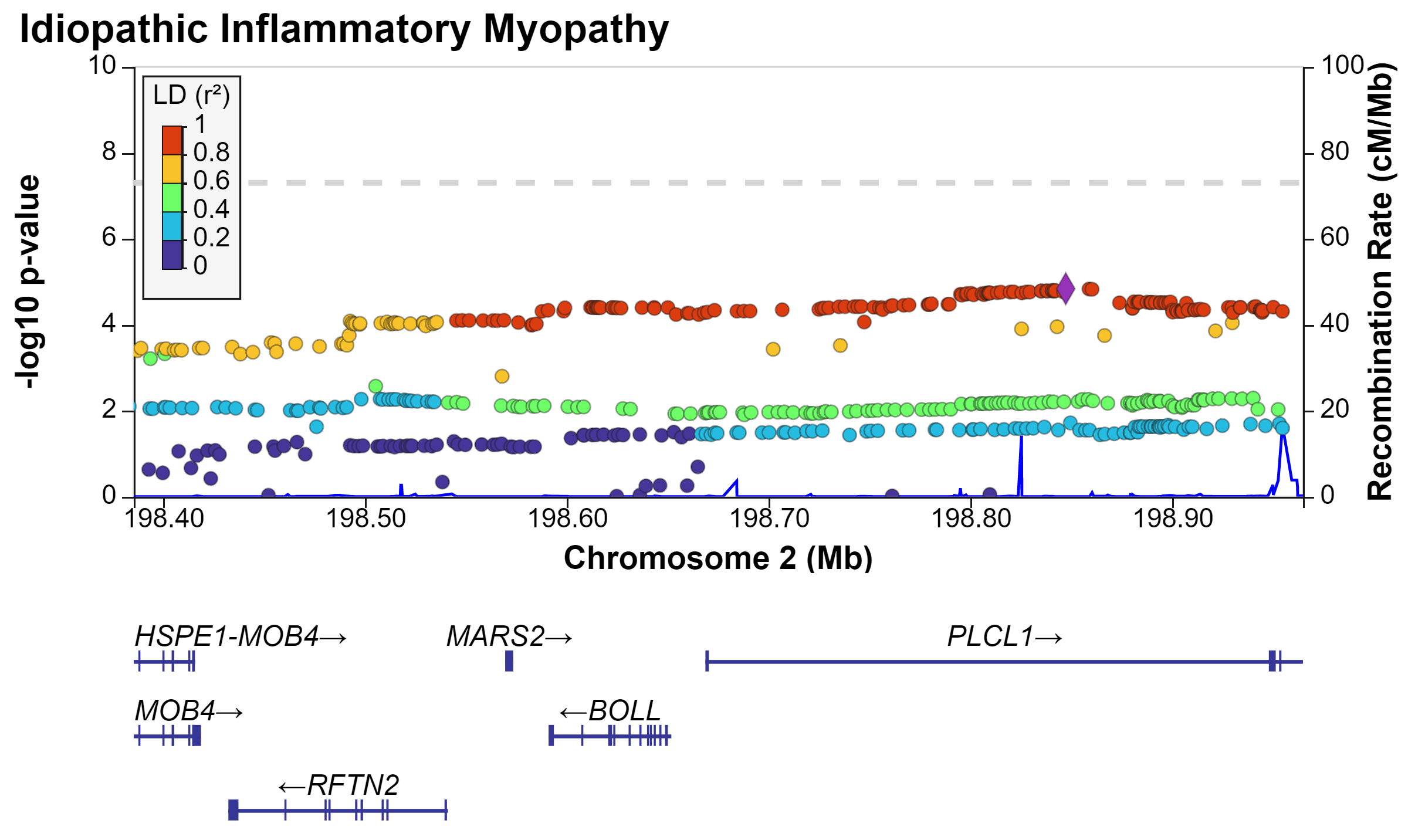


Supplementary Figure 14. Regional association plot of the PLCL1 locus in IIM. The most associated SNP in the region is identified with the purple diamond (rs1518359). SNPs are colour coded by the degree of linkage disequilibrium with the index SNP.


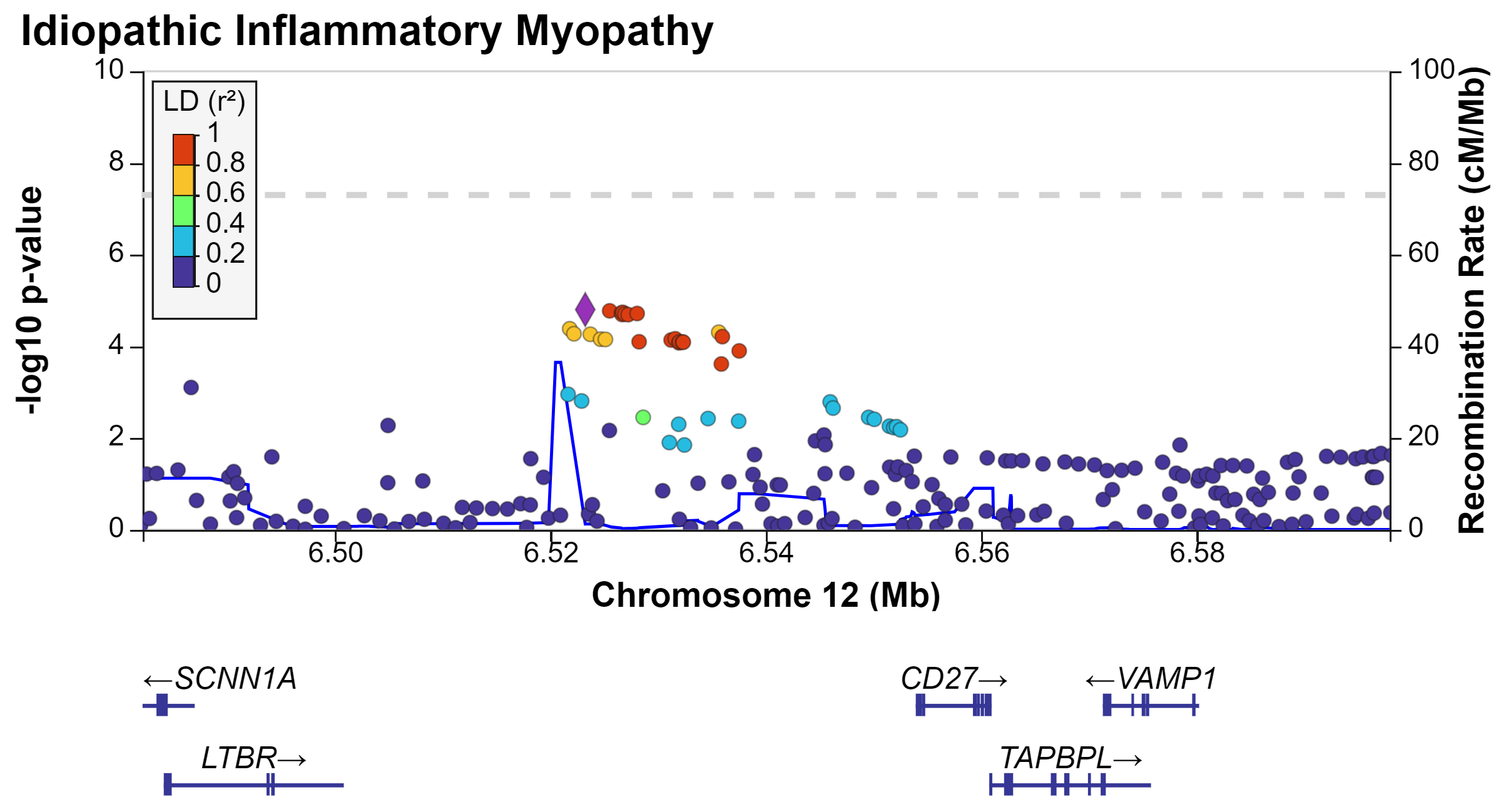


Supplementary Figure 15. Regional association plot of the LTBR locus in IIM. The most associated SNP in the region is identified with the purple diamond (rs11064180). SNPs are colour coded by the degree of linkage disequilibrium with the index SNP.


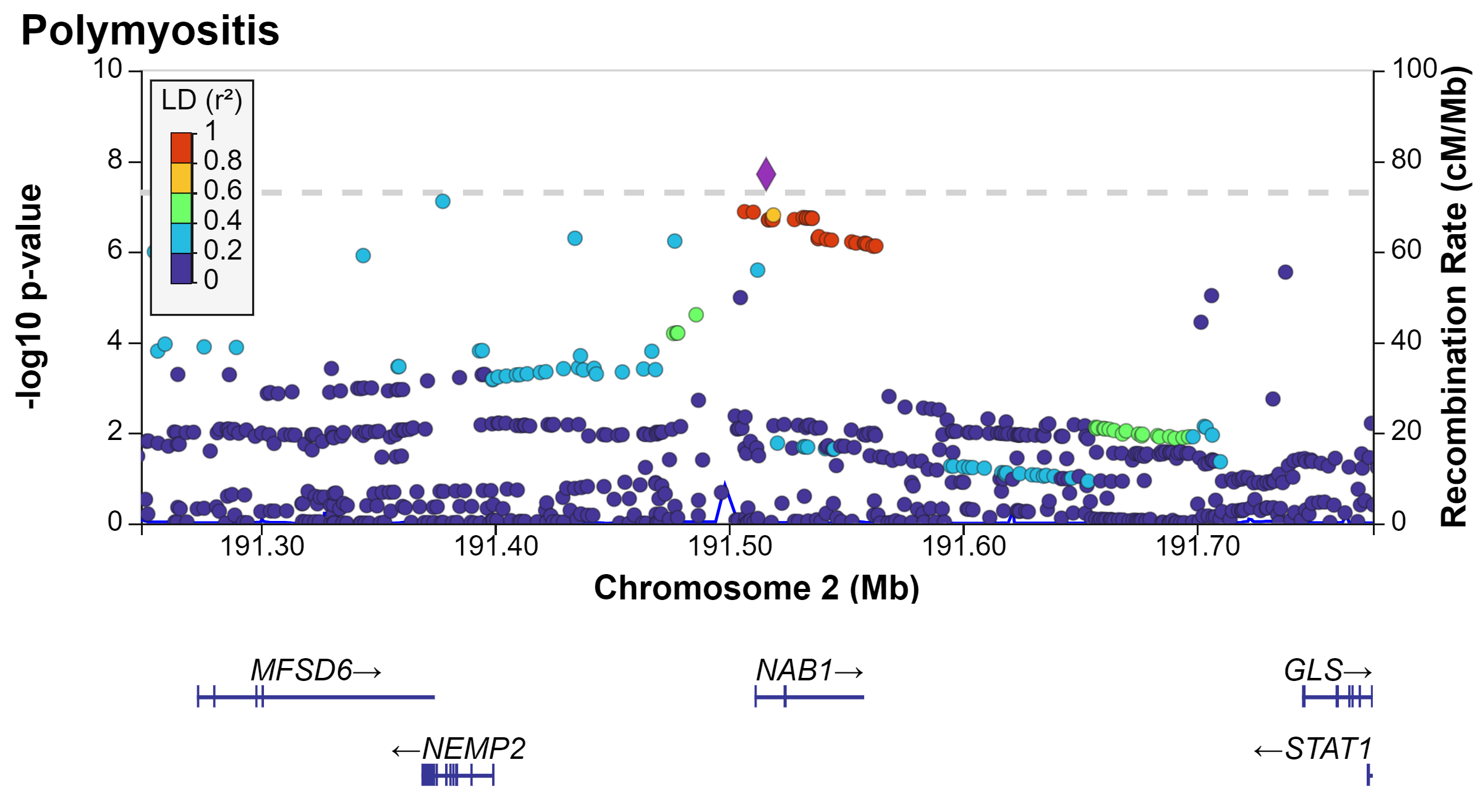


Supplementary Figure 16. Regional association plot of the NAB1 locus in PM. The most associated SNP in the region is identified with the purple diamond (rs6733720). SNPs are colour coded by the degree of linkage disequilibrium with the index SNP.


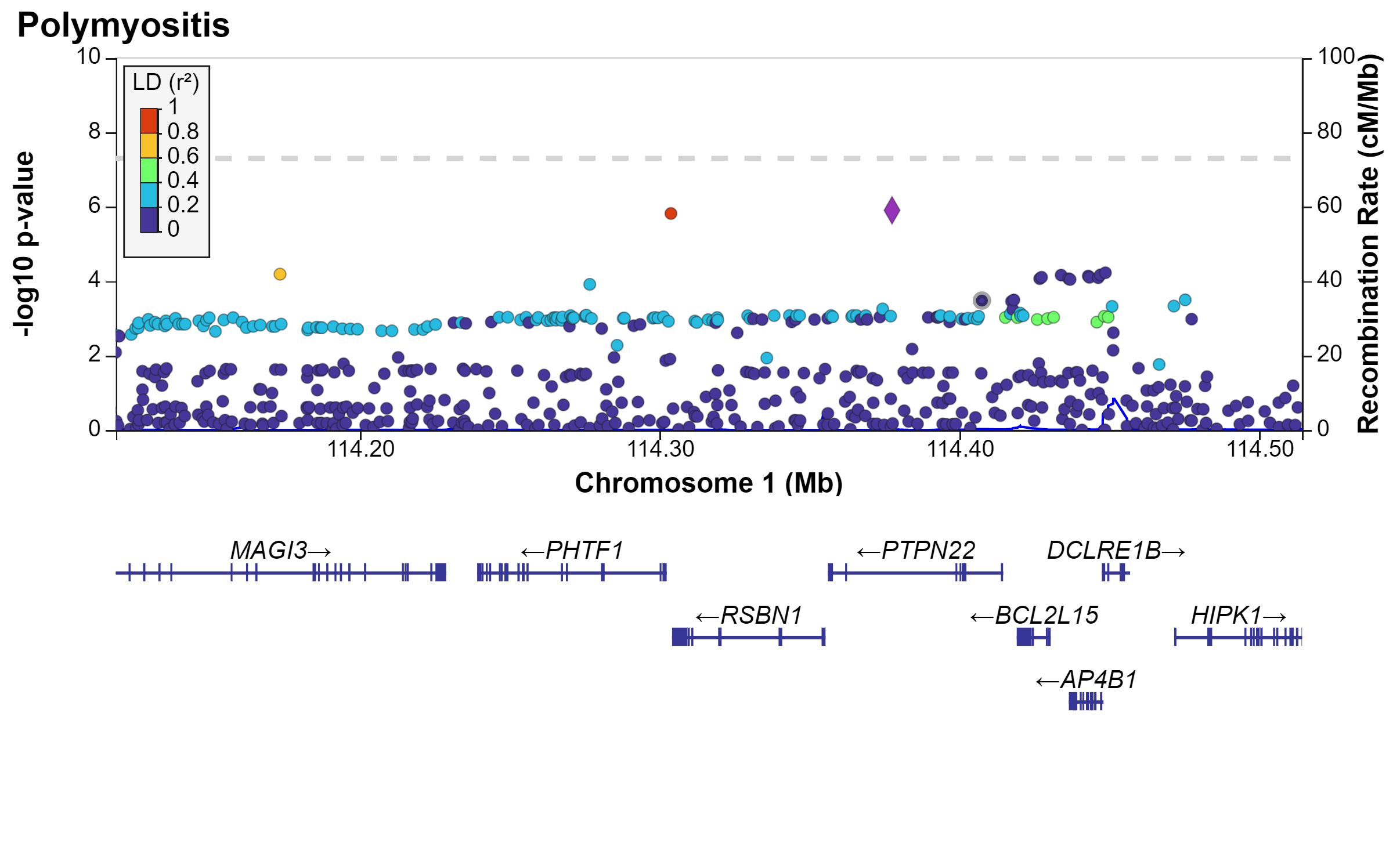


Supplementary Figure 17. Regional association plot of the PTPN22 locus in PM. The most associated SNP in the region is identified with the purple diamond (rs2476601). SNPs are colour coded by the degree of linkage disequilibrium with the index SNP.


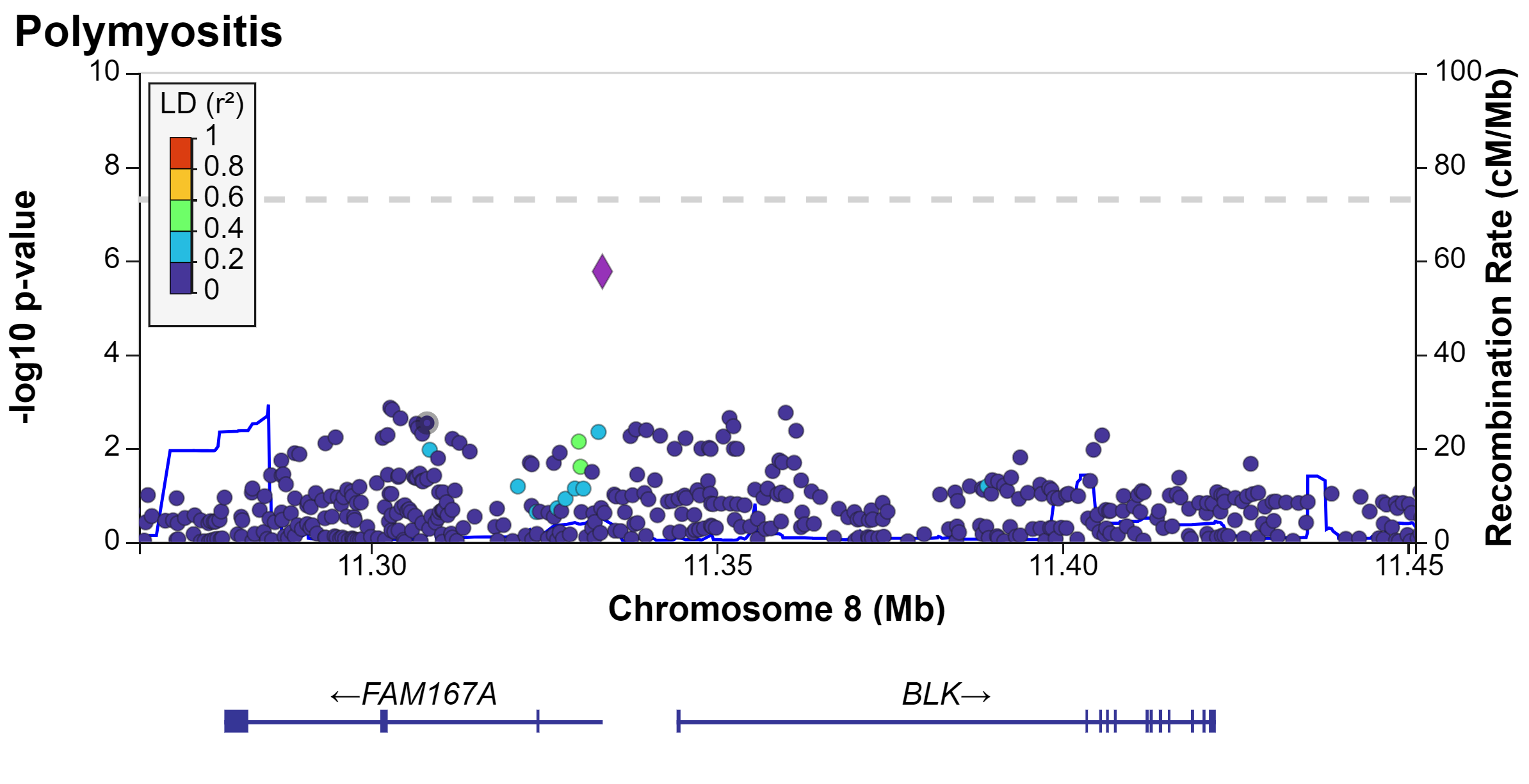


Supplementary Figure 18. Regional association plot of the FAM167A-BLK locus in PM. The most associated SNP in the region is identified with the purple diamond (rs17799348). SNPs are colour coded by the degree of linkage disequilibrium with the index SNP.


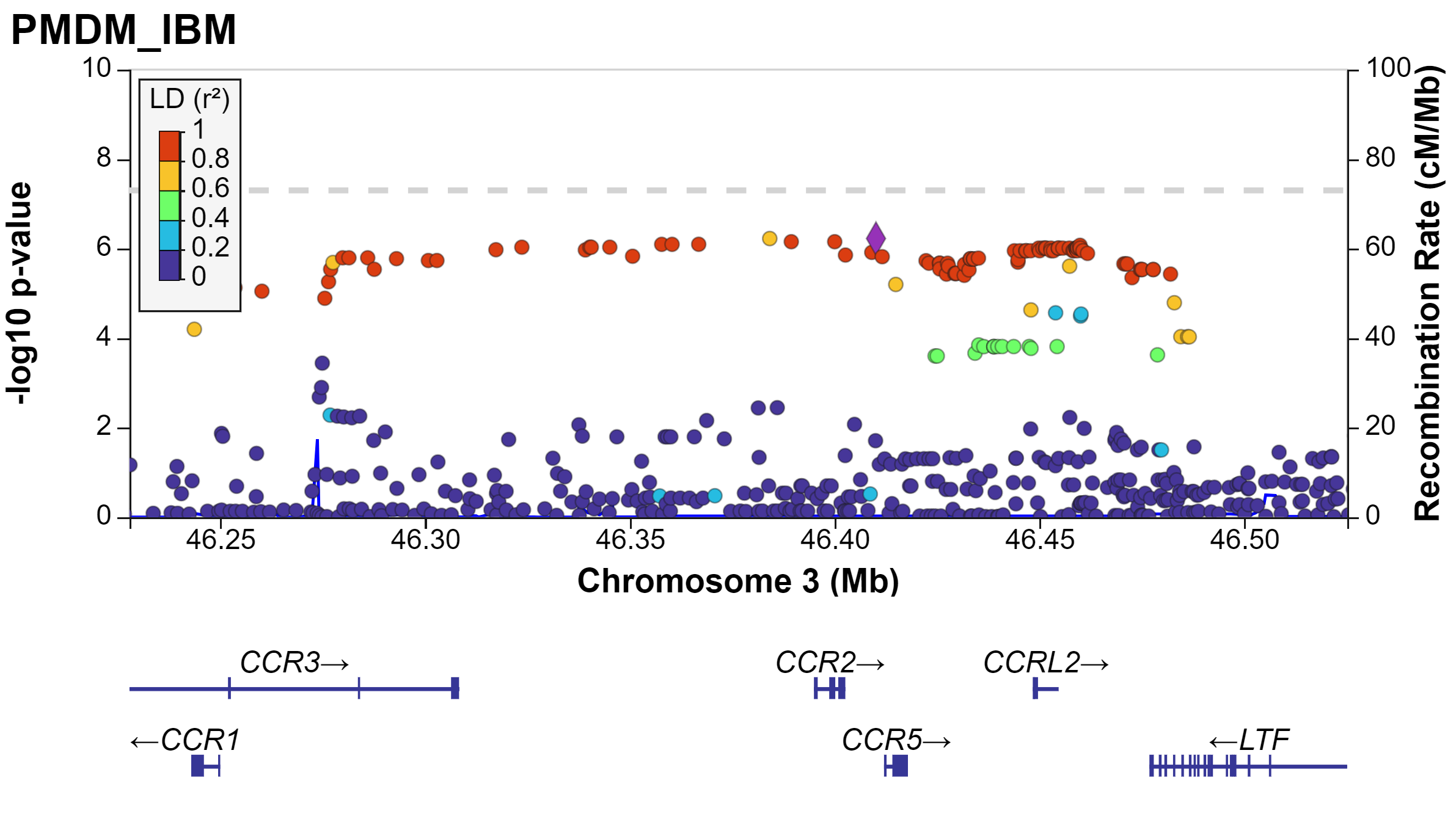


Supplementary Figure 19. Regional association plot of the CCR5 locus in IBM. The most associated SNP in the region is identified with the purple diamond (rs41490645). SNPs are colour coded by the degree of linkage disequilibrium with the index SNP.


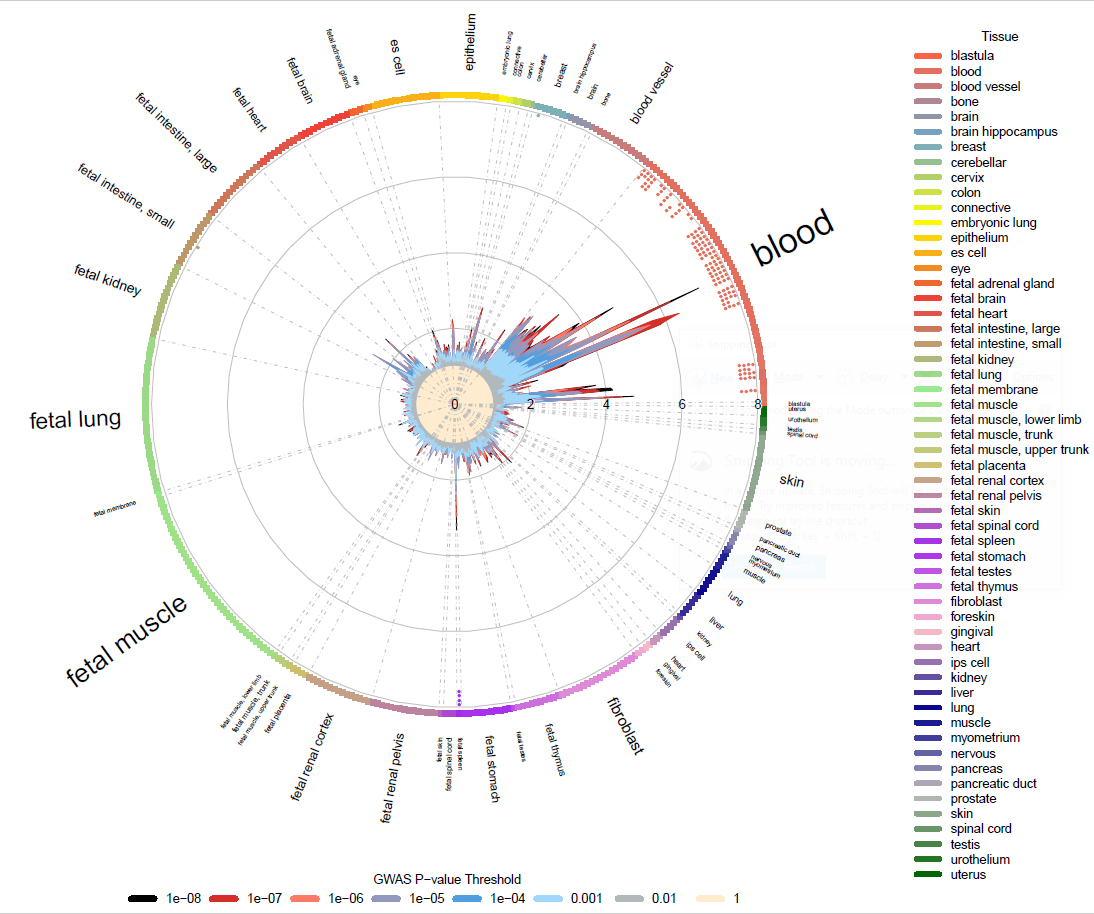


Supplementary Figure 20. GARFIELD analysis of IIM variants. Plot shows enrichment in DNaseI Hypersensitive sites for specific tissue/cell types. Radial plot shows the enrichment within each cell type for each GWAS significance threshold (bottom legend).
